# The Mental Representation of Social Connections: Generalizability Extended to Beijing Adults

**DOI:** 10.1371/journal.pone.0044065

**Published:** 2012-09-11

**Authors:** Louise C. Hawkley, Yuanyuan Gu, Yue-Jia Luo, John T. Cacioppo

**Affiliations:** 1 Department of Psychology & Center for Cognitive and Social Neuroscience, University of Chicago, Chicago, Illinois, United States of America; 2 National Key Laboratory of Cognitive Neuroscience & Learning, Beijing Normal University, Beijing, People's Republic of China; RAND Corporation, United States of America

## Abstract

Social connections are essential for the survival of a social species like humans. People differ in the degree to which they are sensitive to perceived deficits in their social connections, but evidence suggests that they nevertheless construe the nature of their social connections similarly. This construal can be thought of as a mental representation of a multi-faceted social experience. A three-dimensional mental representation has been identified with the UCLA Loneliness Scale and consists of Intimate, Relational, and Collective Connectedness reflecting beliefs about one's individual, dyadic, and collective (group) social value, respectively. Moreover, this mental representation has been replicated with other scales and validated across age, gender, and racial/ethnic lines in U.S. samples. The purpose of this study is to evaluate the extent to which this three-dimensional representation applies to people whose social lives are experienced in a collectivistic rather than individualistic culture. To that end, we used confirmatory factor analyses to assess the fit of the three-dimensional mental structure to data collected from Chinese people living in China. Two hundred sixty-seven young adults (16–25 yrs) and 250 older adults (50–65 yrs) in Beijing completed the revised UCLA Loneliness Scale and demographic and social activity questionnaires. Results revealed adequate fit of the structure to data from young and older Chinese adults. Moreover, the structure exhibited equivalent fit in young and older Chinese adults despite changes in the Chinese culture that exposed these two generations to different cultural experiences. Social activity variables that discriminated among the three dimensions in the Chinese samples corresponded well with variables that discriminated among the three dimensions in the U.S.-based samples, indicating cultural commonalities in the factors predicting dimensions of people's representations of their social connections. Equivalence of the three-dimensional structure is relevant for an understanding of cultural differences in the sources of loneliness and social connectedness.

## Introduction

Social bonds are an inevitable and indispensable part of human existence. The intense reliance of infants on their caregiver for their very survival gives way to a lifetime of connections with other family members, friends, a marital partner, children, and various structured and unstructured groups. Humans experience some of their greatest pleasure from their social relationships [1], and some of their most profound misery when they perceive a deficit in the quantity or quality of their relationships. The latter experience defines loneliness [2], a painful experience that motivates the formation and maintenance of social relationships and recovery of a sense of social connectedness [3].

Given the central role of social connections in human life, and our species' proclivity for making meaning of our existence, it is reasonable to expect that we hold organized mental representations of our social connections. This is, in fact, what we have found. In a large study of primarily Caucasian 18–25 year-old undergraduate students (*N* = 1,255) from a public university in Midwestern USA, an exploratory factor analysis of responses to the 20-item UCLA Loneliness Scale (an instrument that assesses degree of social connectedness) revealed a three-dimensional structure of people's mental representations of their experiences of social connectedness or loneliness [4]. Exploratory factor analyses are useful to explore the possible factors underlying relationships among responses to measured variables, but confirmatory factor analyses are necessary to validate an identified factor structure by statistically testing its fit in an independent data set. Confirmatory factor analyses of responses to the UCLA Loneliness Scale from an independent sample of 18–25 year-old young adults (*N* = 1,276) revealed a good fit of the three-dimensional structure and supported identification of the dimensions as corresponding to Intimate Connectedness (experiences of social value as an individual), Relational Connectedness (experiences of social value in dyadic friendship relationships), and Collective Connectedness (experiences of collective identity and belonging in a group). In addition, the structure did not differ between males and females. Moreover, the three-dimensional structure was not limited to the UCLA Loneliness Scale. Using conceptually related items drawn from a variety of other scales (e.g., Social & Emotional Loneliness Scale; Relational and Collective Interdependence Self-Construal scales; and the Collective Self-Esteem Scale), comparisons of one-, two-, three-, and four-factor models supported the superiority of a three-factor model, and the pattern of factor loadings conformed to the Intimate, Relational, and Collective Connectedness factors observed for the UCLA Loneliness Scale (see footnote 1, [4]).

A lifetime of diverse social experiences and changing values of various types of social relationships might be expected to alter this structure, but a second confirmatory factor analysis using data from a population-based sample of middle-aged and older adults (age = 50–68 yrs; *N* = 229) supported the same three-factor structure in Euro-American, Latino American, and African American men and women from an urban center in the state of Illinois, USA. Racial-ethnic group differences in individualism and collectivism in the U.S. [5] might be expected to influence mental representations of social connections given that these orientations have been experimentally shown to alter self-concept and relationality [6]. However, despite cultural differences in relational patterns and individualistic versus collectivistic beliefs, the three-dimensional structure of people's mental representations of their social world (Intimate, Relational, and Collective Connectedness) fit equally well to data from Euro-American, Latino American, and African American middle- and older age adults [4].

Additional data from the middle- and older-age sample allowed us to validate the identity we assigned the three dimensions. Intimate Connectedness, posited to represent deeply-held beliefs about our individual social value, was uniquely associated (independently of Relational and Collective Connectedness) with being married or living with a partner, consistent with the idea that the spousal relationship lends assurance about one's social worth [7]. Relational Connectedness, posited to represent feelings of closeness and support, was uniquely associated (independently of Intimate and Collective Connectedness), with the number of close friends and relatives with whom participants spoke regularly. Finally, Collective Connectedness, posited to represent feelings of group identification and cohesion, was uniquely associated (independently of Intimate and Relational Connectedness), with number of voluntary group memberships.

The evolutionary importance of valued social connections [8], the heritability of the perceived absence of these connections (i.e., loneliness) [9], and the robustness of the mental representation of these connections that generalized across age, gender, and ethnicity in young and older adult samples in the U.S. [4], prompted the question of the generalizability of this structure to cultural diversities. In the present project, we assessed the fit of the three-dimensional structure of social connectedness (Intimate, Relational, and Collective Connectedness) observed in the United States (U.S.) to data collected in China. For comparison, we note that research on the self and social identity has shown that all people carry a representation of a private, relational, and collective self, even though these selves may be differentially weighted in different cultures or countries and under differing situational circumstances [10].

There are at least two reasons why data from China may exhibit a poor fit to the U.S.-derived structure. First, considerable attitudinal and behavioral differences in the social practices of a collectivistic Chinese culture with more interdependent self-construals, relative to an individualistic American culture with more independent self-construals, are manifested in cultural differences in the prominence of independent versus interdependent self-representations [11], [12]. Despite known social representations of the self across cultures [10], differences in the representational organization of social connections between individualistic and collectivist cultures have not previously been investigated. Although the evidence is mixed, the effects on self-concept (i.e., private, relational, collective) of priming collectivism were smaller in Asian than European American adults [6], suggesting that, in Chinese adults, effects of the collective self on representations of social connections may be at ceiling levels. The factor structure in Chinese adults may therefore favor a dominant collective connectedness factor that is less distinguishable from the intimate and relational factors than has been observed in U.S.-based adults. A failure to replicate a three-factor structure in favor of a one- or two-factor structure (e.g., intimate and collective factors only), or higher correlations of the collective factor with the other factors in the Chinese than U.S.-based samples, would represent support for this hypothesis. Second, younger and older generations in China may differ in the degree of similarity of their mental representations of social connectedness to those of the U.S.-based samples. Generational differences in personal and social values between young and older adults in China are pronounced, thanks in large part to massive social, political, and economic changes over the last century [13]. Chinese young adults growing up in the Social Reform Era (1978 to present), after China's adoption of an “open door policy,” are seen as more individualistic, materialistic, hedonistic, and entrepreneurial than Chinese who grew up during the Consolidation Era (1950–1965) and the Cultural Revolution (1966–1976), when Communist China maintained a “closed door policy” [13]. In addition, whereas the older generation consists predominantly of adults who grew up with siblings, the younger generation consists of a growing proportion of adults who grew up in one-child families. Perhaps not surprisingly, contemporary Chinese young adults have more private and fewer collective self-descriptions and more self-focused memories than older Chinese adults [14]. These contrasting political, cultural, and familial experiences could contribute to a different mental representation of social connectedness in older than in young Chinese adults. Moreover, the three-dimensional structure (Intimate, Relational, and Collective Connectedness) may fit equally well the data from young adults in China as from U.S.-based young adults, but data from older Chinese adults may fit less well than that of younger Chinese adults and U.S.-based older adults. A failure to replicate a three-factor structure in favor of a one- or two-factor structure (e.g., intimate and collective factors only), or higher correlations of the collective factor with the other factors in the older than the younger Chinese and older and younger U.S-based samples, would represent support for this hypothesis.

Importantly, the three-dimensional factor structure (Intimate, Relational, and Collective Connectedness) may accurately capture how Chinese individuals represent their social world yet may nevertheless exhibit inadequate fit if, at the item level, responses are influenced by language differences in the kinds of words that can be used to express social concepts. For instance, the UCLA Loneliness Scale includes items that ask about “no one” (e.g., “There is no one I can turn to”) and others that ask about “people” (e.g., “There are people I can turn to”). The Chinese language does not readily distinguish between singular and plural others. The term, “no one,” is translated using the singular form of the word, “people.” Thus, the English items above become, in Chinese, singular positive (“there are people”) and negative (“no people”) versions of equivalent content. Only one item (“how often do you feel part of a group of friends”) on this scale takes the plural form of “people” that refers to a group. Linguistic differences have social cognitive consequences that could include differences in how social connections are mentally represented [15]. With its dearth of singular terms, we hypothesized that the Chinese language version of the UCLA Loneliness Scale may result in a structure that fails to distinguish between connectedness at the individual, dyadic, and group levels. In our investigation, we therefore distinguished between the generality of mental representations of social connections and cross-cultural linguistic differences.

Finally, knowing the structure of Chinese adults' mental representations of their social connections permits examination of the predictors of each dimension. We follow the factor analyses with tests of association that determine the unique predictors of each loneliness dimension from among a set of theoretically related demographic and social activity variables to determine whether the antecedents of specific dimensions of the mental representation generalize to a collectivist culture.

## Methods

### Participants and Procedure

#### Young Adults

Participants consisted of a convenience sample of 267 undergraduate students between the ages of 16 and 24 years (*M* = 20.4 yrs, *SD* = 1.4) at Beijing Normal University. Data were collected in the summer, after students had completed at least one year of studies. Participants completed questionnaires in groups of up to six individuals, and were compensated 10 yuàn for completing the survey. Each student completed demographic and social activity measures, as well as the R-UCLA Loneliness Scale.

#### Older Adults

Participants consisted of a convenience sample of 246 Chinese adults between the ages of 50 and 68 years (*M* = 58.2 yrs, *SD* = 5.8). Adults participated in groups of up to 20 individuals, and were compensated 50 yuàn for completing the survey. Each participant completed the R-UCLA Loneliness Scale and the same demographic and social activity questionnaires as used in the Chinese young adult sample, and several additional demographic questions about their objective social circumstances.

### Measures

#### R-UCLA Loneliness Scale

The revised UCLA Loneliness Scale is a 20-item questionnaire that assesses subjective feelings of social connection/social isolation [16]. We used version 3 of this scale [17] which differs from the version used in Hawkley et al. [4] by phrasing each item in terms of a question (“how often do you…?”) instead of a statement, and keeps the original response scale. In addition, version 3 alters item 12 by asking how often the participant feels that their social relationships are “not meaningful” as opposed to “superficial,” and alters item 17 by asking how often the participants feels “shy” as opposed to “unhappy being so withdrawn.” The UCLA Loneliness scale possesses high reliability, convergent and discriminant validity, and construct validity [16], [17]. Each item is rated on a four point scale of 1 (never), 2 (rarely), 3 (sometimes), and 4 (always), and responses are summed after reverse-coding appropriate items. The range of possible scores is 20 to 80, with higher scores indicating stronger feelings of loneliness.

The Chinese version of the UCLA loneliness scale was created by having an English major graduate student first translate the scale from English to Chinese, and then having another English major graduate student translate the Chinese version back into English. The two translators then modified the Chinese version to remove divergence between the back-translated English version and the original English version. Three pairs of content-similar items exhibited no distinction in Chinese between what, in English, are singular and plural terms; they did, however, retain a distinction between positively and negatively worded content. These items were 3 (“there is no one I can turn to”) and 20 (“there are people I can turn to”), 7 (“I am no longer close to anyone”) and 10 (“There are people I feel close to”), and 13 (“no one really knows me well”) and 16 (“there are people who really understand me”). The translation of item 12 did not use a negative modifier: “How often do you feel that your relationships with others are not meaningful?” was translated as “How often do you feel that your relationships with others are meaningless?”. A pilot test of the Chinese version was conducted in a young adult sample (*N* = 15) and an older adult sample (*N* = 15) to probe whether there were difficulties in understanding the items. Further modifications were unnecessary.

#### Social Activity

All participants were asked to report the number of close friends and relatives with whom they interacted at least every 2 weeks, and the number of voluntary group memberships (e.g., student unions, social organizations, clubs, civic groups, neighborhood organizations). For analyses, these variables were subjected to a natural log transform to normalize their negatively skewed distributions.

#### Demographics

Participants were asked to report age, gender, years of education, and household income (12 categories, from <35,000 yuàn to >135,000 yuàn). For analyses, household income categories were subjected to a natural log transform to normalize the negatively skewed distribution. Younger adults were also asked about the composition of their family. From the latter was extracted family size and whether they were an only child (1 = yes, 0 = no). Older adults were also asked about their marital status (1 = married or living with a partner; 0 = all others), the identity of family members, and which family members were living in the same household with the parent or parents. From the latter two pieces of information were extracted whether participants had children, number of children, whether they were a single parent, total family size, and household size.

### Data Analysis

We test the fit of the three-dimensional model in young and in middle-aged Chinese adults, with the goal of evaluating the generality of this structure across cultural differences between China and the United States and across generational differences within China. We next examine evidence for cultural differences in responses at the item level. In the Chinese relative to American samples, the absence of a language distinction between terms for singular and plural others may result in greater residual covariance between pairs of content-similar items. Residual covariances are useful to represent the influence of minor factors (e.g., language idiosyncrasies) on overall model fit [18]. If Chinese adults are more likely than U.S. adults to interpret connections with singular others as synonymous with connections with plural others (i.e., a group), then residual correlations among the three item pairs of interest should be positive and larger among Chinese than U.S. adults. If model fit is improved and satisfactory when residual covariances between these item pairs are estimated in the model, the three-factor structure can be interpreted as accurately characterizing how Chinese adults mentally represent their social connections.

The reference confirmatory factor analytic model was based on a sample of 1,276 primarily Caucasian undergraduate students (aged 18–25 years) as reported in Hawkley et al. [4]. In the present study, a confirmatory factor analysis was conducted to test the fit of the data to the three-factor model reported in Hawkley et al. [4]. Maximum likelihood estimation was used to analyze the covariance matrix in MPlus (version 6.1). Latent variable variances were fixed to 1.0, except in the multiple group models in which model identification was achieved by fixing the first loading on each latent variable to one [19]. Fit was assessed using the chi-square, and root-mean-square error of approximation (RMSEA), where an RMSEA of less than .05 is considered a close fit, and .05–.08 is considered reasonable fit [20]. The chi-square difference test and the 90 percent confidence intervals (CIs) around the RMSEA estimate were used to conduct statistical comparisons of model fit.

Regression analyses were conducted to determine the extent to which social activity variables (independent variables) were uniquely predictive of each loneliness dimension (dependent variables) adjusting for the influence of the associated remaining loneliness dimensions. Correlational analyses were conducted to determine associations between demographic variables and the loneliness dimensions.

## Results

### Young Adults

Table 1 presents sample characteristics and descriptive data for each of the measures. Correlations among the UCLA Loneliness Scale items are available in Table S1. All items exhibited significant intercorrelations, with the exception of item 17 which exhibited weak and, in some cases, nonsignificant correlations. Fit of the three-factor model was inadequate, χ^2^ (167) = 467.29, *p*<.001; CFI = 0.804; RMSEA = 0.084, 90% CI = 0.075–0.093. We therefore modified the model by freeing and estimating residual covariances between the three pairs of content-similar items (see Methods). The modified model fit the data well, χ^2^ (164) = 287.11, *p*<.001; CFI = 0.920; RMSEA = .053 (90% C.I.: 0.43, 0.63), and significantly better than the original structure as gauged by a significant chi-square difference test, χ^2^ (3) = 180.18, *p*<.001, and the lack of overlap in the RMSEA confidence intervals. Factor inter-correlations were substantial and exceeded |0.7| (Isolation-Relational = −0.83, Isolation-Collective = −0.76, Relational-Collective = 0.82). Factor loadings, factor intercorrelations, and standard errors from the confirmatory factor analysis are presented in Table 2. Residual variances of the paired items were significantly correlated: items 3 and 20 = −0.451 (*SE* = 0.057), items 7 and 10 = −0.370 (*SE* = 0.056), and items 13 and 16 = −0.597 (*SE* = 0.044). Item 17 exhibited a nonsignificant loading on factor one (0.048) and a large residual variance (standardized variance = 0.998). As noted above, this item, “How often do you feel shy?” was not present in the version of the UCLA Loneliness Scale which generated the three-dimensional reference model; perhaps not surprisingly, this item did not behave well in this context.

**Table 1:** Characteristics of the Beijing younger and older adult sample.

	Younger Adults (N = 267)		Older Adults (N = 246)	
Characteristic	Mean (SD)	Range	Mean (SD)	Range
Age (years)	20.4 (1.4)	16–24	58.2 (5.8)	50–68
Female (%)	56.2		50.0	
Education (years)	14.2 (0.8)	13–16	11.7 (2.8)	3–20
Household income (yuàn, categorical)^1^	4.2 (1.1)	2–12	3.7 (1.6)	1–12
Married/living with partner (%)			85.4	
Have children (%)			93.9	
Number of children (among parents)			1.2 (0.5)	1–4
Only child (%)	45.3			
Family size	3.7 (0.8)	2–6	3.0 (0.7)	1–6
# of close friends & relatives with regular contact	5.5 (3.6)	1–30	5.1 (4.4)	0–30
# of voluntary group memberships	1.4 (1.3)	0–7	0.9 (1.2)	0–10
R-UCLA loneliness score	42.4 (8.5)	23–67	35.2 (10.2)	20–62

1 Category 3 corresponds to 45–55,000 yuàn, and category 4 corresponds to 55–65,000 yuàn.

**Table 2:** Factor loadings in a confirmatory factor analysis of the Revised UCLA Loneliness Scale in Beijing younger and older adults.^1^

Item	Isolation		Relational Connectedness		Collective Connectedness	
	Younger	Older	Younger	Older	Younger	Older
**2. How often do you feel that you lack companionship?**	.545 (.049)	.570 (.048)				
3. How often do you feel that there is no one you can turn to?	.669 (.040)	.579 (.046)				
4. How often do you feel alone?	.571 (.047)	.734 (.034)				
7. How often do you feel that you are no longer close to anyone?	.504 (.051)	.606 (.047)				
8. How often do you feel that your interests and ideas are not shared by those around you?	.446 (.055)	.555 (.048)				
**11. How often do you feel left out?**	.547 (.049)	.728 (.034)				
12. How often do you feel that your relationships with others are meaningless?	.432 (.055)	.540 (.049)				
13. How often do you feel that no one really knows you well?	.612 (.044)	.616 (.043)				
**14. How often do you feel isolated from others?**	.662 (.041)	.721 (.035)				
**17. How often do you feel shy?**	.048 (.066)	.570 (.047)				
18. How often do you feel that people are around you but not with you?	.581 (.046)	.727 (.034)				
**10. How often do you feel close to people?**			.510 (.052)	.613 (.048)		
15. How often do you feel you can find companionship when you want it?			.680 (.041)	.669 (.042)		
**16. How often do you feel that there are people who really understand you?**			.588 (.048)	.751 (.037)		
**19. How often do you feel that there are people you can talk to?**			.611 (.046)	.610 (.047)		
**20. How often do you feel that there are people you can turn to?**			.681 (.042)	.668 (.042)		
**1. How often do you feel that you are “in tune” with the people around you?**					.470 (.059)	.597 (.056)
**5. How often do you feel part of a group of friends?**					.596 (.052)	.592 (.057)
**6. How often do you feel that you have a lot in common with the people around you?**					.527 (.057)	.655 (.052)
**9. How often do you feel outgoing and friendly?**					.522 (.057)	.292 (.069)
**Factor Intercorrelations**						
Isolation	1.00	1.00	−0.83	−0.73	−0.76	−0.68
Relational Connectedness			1.00	1.00	0.82	0.73

**Note**. Standard errors are in parentheses. Items in boldface are the four items chosen to form the subscales.

1 Residual covariances freed between items 3 and 20, 7 and 10, and 13 and 16.

We next tested the equivalence of the fit of the modified model in the Chinese and American young adult samples. A confirmatory factor model that constrained factor loadings and factor intercorrelations to equality across groups, and that allowed the three content-similar items to covary differentially in the two groups, did not fit well, χ^2^ (368) = 3129.05, *p*<.001; CFI = 0.815; RMSEA = 0.099 (90% CI: 0.095, 0.102). Examination of modification indices indicated that the residual covariance between items 19 and 20 should be freed to vary between groups. Doing so resulted in modest fit of the data to the model, χ^2^ (366) = 2269.49, *p*<.001; CFI = 0.873; RMSEA = 0.082, (90% C.I.: 0.079, 0.085), and a significant improvement over the original model as gauged by a significant chi-square difference test, χ^2^ (2) = 859.56, *p*<.001, and a lack of overlap in RMSEA confidence intervals. Factor intercorrelations exceeded |0.7| (Isolation-Relational = −0.83, Isolation-Collective = −0.79, Relational-Collective = 0.83). Residual covariances of the three item pairs that differed in singular versus plural linguistic intent were generally larger in the Beijing than the U.S. sample. The residual correlations (i.e., standardized covariances) for the Beijing and U.S. samples were, respectively, −0.475 (*SE* = 0.054) and −0.269 (*SE* = 0.029) for items 7 and 10, and −0.649 (*SE* = 0.042) and −0.286 (*SE* = 0.029) for items 13 and 16. For items 3 and 20, the pattern differed somewhat: in the Beijing sample, these items exhibited a small positive correlation (0.099, *SE* = 0.019), whereas in the U.S. sample, they exhibited a small negative correlation (−0.182, *SE* = 0.026). It is worth noting that item 3 and 20 are imperfectly translatable; in both cases, the verb, “turn to,” has an object, “for help,” that is not necessary in the English language. In addition, the residual correlation between items 19 and 20 was substantially larger in the Beijing sample (0.955, *SE* = 0.006) than in the U.S. sample (0.549, *SE* = 0.025). Group differences in the residual covariance between items 19 and 20 may have been attributable to limited variance in the U.S. sample: residual variances of these items in the Beijing sample exceeded 0.9 (standardized) but were less than 0.4 in the U.S. sample.

In sum, a constrained model (equality of factor loadings and factor intercorrelations across groups) revealed substantively and, to a modest degree, quantitatively comparable fit of the three-dimensional model in American and Chinese young adults. Chinese young adults, like American young adults, represent loneliness and its opposite, social connectedness, in terms of Intimate, Relational, and Collective facets. This conclusion is tempered by the fact that the pattern of residual covariances suggests an additional facet among Chinese young adults that likely reflects distinctive features of the Chinese language which are themselves potentially meaningful in understanding differences between individualistic and collectivistic cultures. This point is elaborated later.

#### Social activity variables as unique predictors of the loneliness subscales

Subscale scores were generated by summing responses to the same four items for each factor that were employed by Hawkley et al. [4]: Intimate Connectedness (items 2, 11, 14, and 17; reverse-coded to signify connectedness as opposed to loneliness), Relational Connectedness (items 10, 16, 19, and 20), and Collective Connectedness (items 1, 5, 6, and 9), respectively. These three subscales exhibited moderate internal consistency: Cronbach alphas for the intimate, relational, and collective subscales were .61, .71, and .61, respectively. The mean level of loneliness in this young adult sample was 42.4 (*SD* = 8.5; see Table 1). Mean levels of the Connectedness subscales were: *M*_Intimate Connectedness_ = 10.1 (*SD* = 2.3), *M*_Relational Connectedness_ = 13.3 (*SD* = 2.1), *M*_Collective Connectedness_ = 12.9 (*SD* = 1.8). Subsequent results did not differ substantively when the Intimate and Relational subscales were calculated using the four highest-loading items in this young adult Beijing sample instead of the four items used in Hawkley et al. [4].

We next conducted a series of regression analyses to test whether the objective social activity measures were uniquely associated with one over the other Connectedness subscales. Specifically, and as observed in Hawkley et al. [4], we posited that number of close friends and relatives would be uniquely associated with Relational Connectedness, and that number of group memberships would be uniquely associated with Collective Connectedness. Number of close contacts (ln-transform) predicted Relational Connectedness, *B* = 0.434, *SE* = 0.187, *p*<.05, after adjustment for Intimate and Collective Connectedness and number of group memberships. This effect retained significance when demographic variables were also held constant, *B* = 0.446, *SE* = 0.188, *p*<.05. Consistent with prior research (Hawkley et al., 2005), number of group memberships (ln-transform) did not predict Relational Connectedness and did predict Collective Connectedness, *B* = 0.341, *SE* = 0.170, *p*<.05, after adjustment for Intimate and Relational Connectedness and number of close contacts. These results were unchanged when demographic variables were also held constant, *B* = 0.393, *SE* = 0.183, *p*<.05. In addition, number of close contacts had a significant and negative association with Collective Connectedness, *B* = −0.415, *SE* = 0.159, *p*<.01, independent of the effect of Intimate and Relational Connectedness, number of group memberships, and demographic variables.

#### Ancillary analyses

Age, education, and household income were not associated with any of the subscales, *r*'s<.08, *p*'s>.2. Being an only child was associated with higher Intimate Connectedness scores, *p*<.05 (Table 3), and lower total loneliness scores tended to mirror this difference, *p*<.09. Consistent with this finding, family size was positively associated with loneliness, *r* = .11, although this correlation only approached significance, *p*<.09. Gender differences were also evident (Table 3): females were less lonely than males, *p*<.05, and this difference was echoed in higher Relational and Collective Connectedness scores among females than males, *p*'s<.05. Males and females were equally represented in the two family types (44% males in only child families vs. 45% males in multi-child families), χ^2^ = 0.251, *p*>.05, so gender composition in the different family types are not a plausible explanation for loneliness differences between family types.

**Table 3:** Group differences in loneliness and connectedness subscales (Beijing young adults).

	UCLA Loneliness	Intimate Connectedness	Relational Connectedness	Collective Connectedness
Gender				
Male	43.6 (8.7)*	10.0 (2.3)	13.0 (2.2)*	12.7 (1.9)*
Female	41.4 (8.3)	10.2 (2.3)	13.5 (2.0)	13.1 (1.7)
Only child				
Yes	41.4 (8.3)	10.4 (2.2)*	13.3 (2.1)	13.1 (1.8)
No	43.2 (8.6)	9.8 (2.4)	13.3 (2.1)	12.8 (1.8)

* significant at the 0.05 level (2-tailed).

** significant at the 0.01 level (2-tailed).

### Older Adults

Table 1 presents sample characteristics and descriptive data for each of the measures. Correlations among the UCLA Loneliness Scale items are available in Table S2. All items exhibited significant intercorrelations, with the exception of items 5 and 9 which exhibited weak and nonsignificant correlations with some items.

Fit of the three-factor model was inadequate, χ^2^ (167) = 506.64, *p*<.001; CFI = 0.824, RMSEA = 0.091 (90% CI = 0.082–0.100). The model was therefore modified as was done for the young adult sample, namely by freeing and estimating residual covariances between the three pairs of content-similar items (items 3 & 20, 7 & 10, 13 & 16). The modified model fit the data reasonably well, χ^2^ (164) = 356.78, *p*<.001; CFI = 0.900, RMSEA = .069 (90% C.I.: 0.59, 0.79) and significantly better than the original model as gauged by a significant chi-square difference test, χ^2^ (3) = 149.86, *p*<.001, and the lack of overlap in the RMSEA confidence intervals. Factor loadings, factor intercorrelations, and standard errors from the confirmatory factor analysis are presented in Table 2. Factor inter-correlations were substantial (|r|s≥.67). Residual variances of the paired items were significantly correlated: items 3 and 20 = −0.189 (*SE* = 0.068), items 7 and 10 = −0.339 (*SE* = 0.063), and items 13 and 16 = −0.707 (*SE* = 0.042).

We next tested the equivalence of the fit of the modified model to the Chinese and American older adult data. The Beijing older adult sample and the confirmatory sample of U.S. older adults (*N* = 228) tested in Hawkley et al. (2005) were used for this purpose. A confirmatory factor model that constrained factor loadings and factor intercorrelations to equality across groups, and that allowed the residuals of three items in question to covary differentially in the two groups, exhibited modest fit, χ^2^ (368) = 938.34, *p*<.001; CFI = 0.853, RMSEA = 0.081 (90% CI: 0.074, 0.087). Residual covariances of the three item pairs in question were larger in the Beijing than the U.S. sample. The residual correlations (i.e., standardized covariances) for the Beijing and U.S. samples were, respectively, −0.214 (*SE* = 0.066) and −0.075 (*SE* = 0.081) for items 3 and 20, −0.320 (*SE* = 0.064) and −0.077 (*SE* = 0.075) for items 7 and 10, and −0.710 (*SE* = 0.040) and −0.103 (*SE* = 0.074) for items 13 and 16. The fact that a relatively constrained model (equality of factor loadings and factor intercorrelations across groups) produced at least modest fit suggests that the three-dimensional mental representation of social connections does not differ substantively between American and Chinese older adults.

Importantly, we also tested the equivalence of the model fit across the younger and older generations in China. For this test, in addition to constraining factor loadings and intercorrelations to equality, residual covariances of the three item-pairs were also constrained to equality across the two groups. This test revealed modest fit, χ^2^ (371) = 945.53, *p*<.001; CFI = 0.836, RMSEA = 0.078 (90% CI: 0.072, 0.084). Freeing the residual covariances did not alter model fit, as would be expected given that the comparison of young and older Chinese was based on responses to the same Chinese translation of the UCLA Loneliness Scale.

#### Social activity variables as unique predictors of the loneliness subscales

Subscale scores were generated by summing responses to the same four items for each factor that were employed in Study 1 and by Hawkley et al. [4]. These three subscales exhibited adequate to good internal consistency: Cronbach alphas for the Intimate, Relational, and Collective subscales were .72, .77, and .58, respectively. The mean level of loneliness in this older adult sample was 35.2 (*SD* = 10.2; see Table 1). Mean levels of the Connectedness subscales were: *M*_Intimate Connectedness_ = 12.6 (*SD* = 2.8), *M*_Relational Connectedness_ = 13.4 (*SD* = 2.4), *M*_Collective Connectedness_ = 13.6 (*SD* = 2.0).

We posited that marital status would be uniquely associated with Intimate Connectedness, and this is what we found. Consistent with prior research in a U.S. older adult sample [4], being married predicted higher levels of Intimate Connectedness, *B* = 0.894, *SE* = 0.438, *p*<.05, after adjustment for Relational and Collective Connectedness, number of close contacts, and number of group memberships. This effect was retained when demographic variables (age, gender, education, income) were also held constant, *B* = 1.074, *SE* = 0.445, *p*<.05. Neither number of close contacts nor number of group memberships predicted Intimate Connectedness, *p*'s>.5. Likely because of the high intercorrelations among the subscales, Relational and Collective Connectedness predicted Intimate Connectedness over and above the effect of being married, *p*'s<.06.

Consistent with prior research [4] and with results observed for young adults in Beijing (Study 1), number of close friends and relatives (ln-transform) predicted Relational Connectedness, *B* = 0.504, *SE* = 0.199, *p*<.05, after adjustment for Intimate and Collective Connectedness, marital status, and number of group memberships. This effect remained significant when demographic variables were also held constant, *B* = 0.493, *SE* = 0.216, *p*<.05. Neither marital status nor number of group memberships predicted Relational Connectedness, *p*'s>.4. Intimate and Collective Connectedness predicted Relational Connectedness over and above the effect of being married, *p*'s<.01.

Number of group memberships (ln-transform) exhibited a modest bivariate association with Collective Connectedness, *B* = 0.422, *SE* = 0.248, *p* = .09, but did not predict Collective Connectedness after adjustment for Intimate and Relational Connectedness, marital status, and number of close contacts, *B* = 0.218, *SE* = 0.216, *p*>.3. This association was further diminished when demographic variables were also held constant. Group memberships of older Chinese adults may be of a different type than those in the U.S. or those of younger Chinese (e.g., differences in degree of social interaction within the groups), a possibility we did not have the data to address. Neither marital status nor number of close contacts predicted Collective Connectedness, *p*'s>.2. Intimate and Relational Connectedness continued to exhibit associations with Collective Connectedness in the fully adjusted model, *p*'s<.06.

#### Ancillary analyses

As shown in Table 4, age was inversely correlated with loneliness, *r* = −.16, *p*<.05, and this association with age was also evident for Relational Connectedness and, less reliably, for Intimate and Collective Connectedness. A significant gender difference in total loneliness scores (Table 5) revealed higher levels in males than females, *p*<.05, but this difference was not evident for any of the Connectedness subscales, *p*'s>.08. Years of education exhibited modest correlations with loneliness and each subscale (Table 4), but the only significant correlation was with Intimate Connectedness, *r* = .13, *p*<.05. Household income (ln-transformed) exhibited significant correlations with loneliness, *r* = −.17, *p*<.05, and with the Relational and Collective Connectedness subscales, *p*'s<.01. Household income was not associated with Intimate Connectedness, *p*>.1.

**Table 4:** Correlations of loneliness and connectedness subscales with demographic and social activity variables (Beijing older adults).

	UCLA Loneliness	Intimate Connectedness	Relational Connectedness	Collective Connectedness
Age	−.159*	.123	.170**	.123
Education	−.121	.127*	.113	.105
Household income^1^	−.170**	.091	.179**	.188**
Number of close contacts^1^	−.227**	.153*	.248**	.168**
Number of group memberships^1^	−.116	.060	.097	.102
Household size	−.094	.098	.066	−.033
Family size	−.205**	.136*	.217**	.124

1 Variable was subjected to a natural log transformation.

* significant at the 0.05 level (2-tailed).

** significant at the 0.01 level (2-tailed).

**Table 5:** Group differences in loneliness and connectedness subscales (Beijing older adults).

	UCLA Loneliness	Intimate Connectedness	Relational Connectedness	Collective Connectedness
Gender				
Male	36.6 (10.6)*	12.3 (2.9)	13.3 (2.4)	13.4 (2.1)
Female	33.9 (9.6)	12.9 (2.8)	13.4 (2.4)	13.8 (1.9)
Marital status				
Married	34.3 (9.9)**	12.8 (2.7)**	13.5 (2.3)*	13.7 (2.0)*
Unmarried	40.5 (10.0)	11.2 (2.9)	12.4 (2.8)	12.8 (2.1)
Have children				
Yes	35.0 10.0)	12.6 (2.8)	13.4 (2.4)	13.6 (2.0)
No	38.2 (12.5)	12.1 (3.2)	13.2 (2.0)	13.0 (2.4)
Have only one child				
Yes	35.7 (10.1)*	12.5 (2.8)	13.1 (2.5)**	13.6 (2.0)
No	32.4 (9.1)	12.9 (2.6)	14.4 (2.0)	13.9 (1.8)
Single parent				
Yes	39.6 (9.3)*	11.3 (2.5)*	12.4 (2.9)*	13.0 (2.1)
No	34.5 (10.0)	12.8 (2.8)	13.5 (2.4)	13.7 (1.9)
Religious affiliation				
Yes	40.4 (9.6)	11.7 (2.5)	12.3 (2.3)	12.7 (2.7)
No	35.0 (10.1)	12.6 (2.8)	13.4 (2.4)	13.6 (1.9)

* significant at the 0.05 level (2-tailed).

** significant at the 0.01 level (2-tailed).

Marriage had a pronounced association with loneliness such that each subscale showed the advantage of being married, *p*'s<.05. This like reflects the high intercorrelation among the subscales and the known importance of marriage for lowering feelings of loneliness. Total loneliness scores averaged 34.3 (*SD* = 9.9) for married participants and 40.5 (*SD* = 10.0) for unmarried participants. Connectedness subscale scores are presented as a function of marital status in Table 5. Males were more likely to be married than females, χ^2^ (267) = 4.686, *p*<.05, and the marriage difference in loneliness persisted after adjusting for gender. Most of the adults in this sample had children, and the few that had no children (*N* = 15, or 6.1% of the sample) did not differ significantly in loneliness and Connectedness subscale scores. Notably, however, the magnitude of the difference in total loneliness between the two subgroups was sizeable, nearly as large as the gender difference in loneliness (*M*_childless_ = 38.2, *SD* = 12.5; *M*_parents_ = 35.0, *SD* = 10.0), and this difference was reliably reflected in Intimate Connectedness and Collective Connectedness scores (Table 5). As would be expected given China's one-child policy in urban areas, most of the parents in this sample had only one child (185 of 231 parents, or 80% of parents). One-child parents were more lonely (*M* = 35.7, *SD* = 10.1) than their multi-child counterparts (*M* = 32.4, *SD* = 9.1), *p*<.05, and this effect was reflected in significantly lower levels of Relational Connectedness among one-child parents than multi-child parents (Table 5). Similarly, family size was inversely associated with loneliness (*r* = −.21, *p*<.05) and reliably positively associated with each of the Connectedness subscales (Table 4).

Among parents, 23 participants (approximately 10 percent of parents) were the sole parent in the household. These single parents were significantly lonelier (*M* = 39.6, *SD* = 9.3) than their counterparts in dual-parent households (*M* = 34.5, *SD* = 10.0). This effect was evident in significantly lower levels of Intimate Connectedness and Relational Connectedness among one-parent households than dual-parent households (Table 5). Household size, however, was unrelated to loneliness and the Connectedness subscales, *p*>.1.

### Supplementary Analyses

To lend support to our assertion that three factors corresponding to Intimate, Relational, and Collective Connectedness capture people's mental representations of their social relationships in the U.S. and China, we conducted multidimensional scaling (MDS) analyses to determine the robustness of the factor analytic structure to differences in analytic methods and to verify and assist in interpretation of the dimensions. The U.S. young adult sample [4] was used to examine the plausibility of a two or three-dimensional structure against which the three remaining samples could be compared for consistency (Figure S1). Treating responses on an ordinal scale, the stress index for the two-dimensional structure was 0.112, and for the three-dimensional structure was 0.068, where stress values can range from 0 to 1 and the smaller the stress function, the better the representation of the data. Also, relative to the two-dimensional solution, the three-dimensional solution proved conceptually informative. The first dimension varied in degree of supportive interpersonal relationships, ranging from their presence (e.g., “feel close to people”) to their absence (e.g., “feel isolated”). This dimension corresponded well to the factor analytic dimension of Relational Connectedness. The second dimension varied in degree of authenticity and ranged from absence of authentic others (e.g., “lack companionship”) to presence of inauthentic others (“social relationships are superficial”). This dimension was informative in refining our interpretation of the Intimate Connectedness factor. Specifically, the sense of enhanced self-worth that accompanies genuine acceptance and valuation by others differentiates those high versus low in Intimate Connectedness. The third dimension varied in degree of social breadth and ranged from the individual or dyad (e.g., “no longer close to anyone”) to the collective (e.g., “have a lot in common with the people around me”). This dimension corresponded well to the factor analytic distinction between Relational and Collective Connectedness.

In sum, the three-dimensional structure replicated the three-factor solution and extended it by revealing that the Intimate Connectedness factor reflects differences in degree of relational authenticity. Subsequent MDS analyses in the U.S. older adult sample and the Beijing young and older adult samples revealed essentially equivalent dimensions and interpretations. These analyses are available in Figures S2, S3, S4.

## Discussion

Humans are a meaning-making species born to the longest period of dependency of any species and are dependent on conspecifics across the lifespan to survive and prosper. We have posited that the social reward of feeling connected to others and the social pain of feeling disconnected serve an adaptive function, namely to motivate the formation, maintenance, and nurturing of social relationships that promote survival [3]. Moreover, we have posited that each dimension of the phenotypic expression of loneliness/social connectedness—Intimate, Relational, and Collective Connectedness—serves a unique adaptive function [21]. Our evolutionary model implies that the multi-dimensional loneliness phenotype has a universal structure that holds across gender, age, and cultural lines. Environmental (e.g., cultural) influences may result in differential experiential weighting of the three dimensions [22], such that between- and within-country differences in independent/interdependent or individualistic/collectivistic orientations may result in greater sensitivity of Intimate, Relational, and Collective Connectedness to some and not other aspects of the social context. Despite these differences, the three-dimensional structure should adequately represent the mental representations of social connections across contexts.

Prior research has shown that a three-dimensional model of people's mental representations of their social connections fits equally well in males and females, and in young (18–25 yr-old) and older (50–68 yr-old) ethnically diverse adults in the U.S. [4]. The present confirmatory analyses indicate that the three-dimensional model also applies to young and older adults in China, thus providing the best evidence to date for the existence of a universal mental representation of one's social world that consists of individual, relational, and collective aspects. In addition, the subscales that represented the three dimensions exhibited a pattern of associations with social activities in both young and older Chinese adults that corresponded to associations observed in older American adults [4]. Specifically, when the remaining two subscales were held constant, Intimate Connectedness was uniquely associated with being married and not with number of close contacts or group memberships, Relational Connectedness was uniquely associated with number of close friends and relatives and not with marital status or number of group memberships, and Collective Connectedness was uniquely associated with number of group memberships (in young but not significantly in older adults) and not marital status or number of close contacts.

One important caveat regarding the factor structure in the Beijing samples is that the translated loneliness questionnaire introduced unavoidable idiosyncrasies that generated an apparent minor factor reflecting linguistic characteristics of the written Chinese language. Specifically, the Chinese language does not easily permit distinguishing between singular and plural forms of terms for social others [24]. For instance, in the English language, the term “people” is distinct from the term “one” (e.g., in anyone, no one); “people” connotes a plurality of individuals, not a group per se. In the Chinese language, a plural version of “people” in Chinese requires the addition of a plural modifier (群) that connotes a single group of people (e.g., UCLA item #18); in contrast, the appropriate Chinese term for “people” (人) (e.g., UCLA items 10, 16, and 20) is identical to the term for the singular “one” (人) (e.g., UCLA items 3, 7, and 13). A linguistic study is needed to shed light on whether the absence of a distinction between characters for people and individual persons reflects ancient cultural beliefs and values that favor the collective over the individual.

### Correlates of connectedness/loneliness and its factors

Demonstration of a comparable factor structure does not mean that Chinese exhibit the same associations of social and demographic variables with loneliness and each of the Connectedness subscales as are evident in U.S. samples. In the U.S., age tends to be inversely correlated with loneliness, at least in middle age cohorts [25], [26]. The same finding was true in the middle age cohort from Beijing, suggesting that similar age-related changes in cognitive, affective, and behavioral processes may be functioning. For instance, mainland Chinese, like American adults [27], exhibit a preference for emotionally close social interaction partners over less familiar social partners when perceptions of time are constrained by experimental and naturalistic manipulations [28], [29]. This finding is consistent with socioemotional selectivity theory [30] and suggests that age-related shifts in social goals may help to explain age-related decrements in the intensity of loneliness in the Chinese as they do in the U.S. This conjecture is further supported by our finding that age was significantly associated only with the Relational and not the Intimate or Collective Connectedness subscales. To the extent that Relational Connectedness reflects a preference for and choice of close social interaction partners over less familiar partners, this is the dimension of Chinese adults' social world that is the most robustly associated with the age-related decline in loneliness.

Gender differences in loneliness are inconsistently observed in the U.S., and the direction of the difference is also inconsistent [25]. In the present Chinese samples, loneliness was significantly higher in young and older adult males than in their age-matched female counterparts. No single dimension of Connectedness appears responsible for the gender difference in loneliness. Little research has been conducted in this area among Chinese samples, and of this work, loneliness has been shown to be less prevalent among males than females in rural-to-urban migrants in Shanghai [31]. Relatedly, marital satisfaction and life satisfaction were higher in Chinese Malaysian men than women [32]; loneliness differences would be expected to follow a similar pattern. Thus, existing research shows the same kind of inconsistency in gender differences in loneliness as has been observed in U.S. samples.

Income and education were not associated with loneliness or any of the Connectedness subscales in the Beijing young adult sample, but did exhibit correlations in the older adult sample. Consistent with prior research in U.S. older adults [25], household income was inversely associated with loneliness. Socioeconomic status is posited to influence loneliness by affecting opportunities for social interactions with those outside the immediate family [25]. This should be evident in a more robust association between income and Relational or Collective Connectedness than with Intimate Connectedness, and that is the pattern of results we observed. For education, on the other hand, correlations were more modest, and only Intimate Connectedness had a significant inverse association with education.

### Age differences in loneliness: Beijing young adults are most afflicted

Loneliness levels among the Beijing young adults averaged 42.4, substantially higher than the mean of 37.0 (*SD* = 11.0) in U.S. young adults [4], 35.0 (*SD* = 9.8) in U.S. older adults [33], and 35.2 in Beijing older adults in the present study. The fact that young Beijing adults differed in loneliness from their older Beijing counterparts argues against linguistic differences (i.e., translation of loneliness items to Chinese) in interpretation and ratings of items and suggests instead that the experience of loneliness is itself more intense in Beijing young adults.

This is not the first study to find higher levels of loneliness in Chinese young adults. For instance, Anderson [34] found that young college students in Shanghai, China, were significantly lonelier than their counterparts in a Midwestern city in the USA. However, our U.S. young adult sample was tested in 1999, whereas the Beijing young adults were tested in 2009. To rule out a period effect as an explanation for differences in loneliness intensity, we compared loneliness levels of the Beijing young adults with the loneliness levels of U.S. young adults tested at approximately the same time. However, data collected in undergraduate male students at the Ohio State University in 2010 revealed a similar mean level of loneliness, 36.6 (*SD* = 9.2, *N* = 55; unpublished data), as had been observed in the 1990's, substantially lower than levels seen in the Beijing young adults. Wang et al. [35] found even higher levels of loneliness (*M* = 45.98, *SD* = 8.67) in Chinese high school students in Hong Kong than we found in the present study. Thus, our results are consistent with what has been observed in prior research. Additional research is needed to determine the source of this phenomenon (i.e., why are urban Chinese young adults lonelier than their U.S.-based counterparts), its breadth (e.g., are rural Chinese young adults lonelier than rural U.S. young adults?), and its duration (i.e., what factors contribute to the alleviation of loneliness with age and time?).

Adjustment to the university context is known to affect loneliness in U.S. students [36] and undoubtedly also affects Chinese students. Precisely because the first year at university is unsettling, we required that students in both the U.S. and Beijing samples had completed at least a year of studies. By the end of the first year of studies, most students have established new social connections in their university community and will have become accustomed to life away from their family. For some U.S. students, however, a year is insufficient time to recover from the social shock of leaving home and attending university [36]. Additional research is needed to determine the duration of the adjustment period in Beijing students and whether loneliness eventually decreases to levels that more closely resemble levels in U.S. students or older adults in Beijing.

What is unique in the Chinese young adults that might account for their high levels of loneliness? Higher rates of one-child families may contribute to differences in the level of loneliness in the young adult population, but the direction of the effect of family type could go either way. The absence of siblings could spell a more solitary childhood, perhaps deprived of social opportunities and social skill learning that help minimize risk for loneliness. On the other hand, an only child likely receives relatively undivided attention from the parents, and interactions between parent and child may be conducted on a more sophisticated level than might be possible when competing with siblings for the parents' attention. Moreover, the only child may be offered more opportunities to interact with peers of their choice (vs. obligatory interactions with siblings), a circumstance that is likely to maximize their ability to form good quality social relationships and minimize their loneliness. In the only relevant American study to date, loneliness in females increased as a function of the number of siblings [37]. What our Chinese data showed was that being an only child, relative to having siblings, was associated with lower loneliness. The one-child policy has been more successfully implemented in urban than in rural areas of China, and loneliness is higher in rural than in urban areas of China [38], so one caveat to this finding is that if the 55 percent of the young adults in our sample that came from multi-child families were born in rural areas, the loneliness difference between family types may be attributable to an urban-rural difference rather than a family type difference. Additional data are needed to test this possibility. Notably, however, the loneliness difference between family types was small in magnitude; even only children had high levels of loneliness relative to U.S. young adults.

Intimate but not Relational or Collective Connectedness echoed the loneliness difference between only-child and multi-child family types. Intimate Connectedness has been associated with having a spouse or live-in partner [4], as was also evident in the older Beijing adult sample studied here, raising the possibility that young adult only children were more likely than those with siblings to have a significant romantic partner. Another possibility is that Intimate Connectedness may be higher to the extent that China's increasingly child-centered society encourages channeling of parental and grandparental resources to the development and care of the only child, an orientation that maximizes the probability that the only child will feel their core self-affirmed and their belief in their individual social value strengthened more so than those with siblings. Consistent with this conjecture, loneliness levels were higher, and Intimate Connectedness levels lower, in young adults from larger size families.

Family size means something quite different to parents, however. Replicating and extending prior research among older Chinese adults [39], in our sample of older adults from Beijing, those with larger families (and multi-child families relative to only-child families) had lower levels of loneliness and higher levels of Intimate as well as Relational and Collective Connectedness. Possibly, a larger family provides a greater sense of personal fulfilment and identity for older parents (Intimate Connectedness), more relationship opportunities through a larger network of friends or family members (Relational Connectedness), and a greater sense of fit in a society in which the family, although shrinking in size, is still the most important collective (Collective Connectedness) [40]. Additional research is needed to address these possibilities.

#### Limitations

Research to date on the generalizability of mental representations of loneliness is far from exhaustive, and additional research is needed to evaluate whether the three-dimensional structure holds up across a variety of other diversities. For instance, does the three-dimensional structure fit the Chinese in the U.S. as well as it does the Chinese in China and Americans in the U.S.? Does it fit as well among rural as among urban adults in China and the U.S.? In addition, to what extent do subcultures within a country (e.g., Goth, Amish) differ in their mental representations of their social connections?

The exponential growth in online social networking adds a layer of complexity to people's social relationships. Whether social networking activity has changed or will change people's mental representations of their social connections is an open question. It is likely, however, that the influence of social networking on mental representations will depend at least in part on how people use virtual social networks. For instance, future research could examine differences in the mental representations of social connections among people whose virtual networks are integrated with and a subset of their “real” networks versus those whose virtual networks are distinct from their real networks.

Even if the structure of the mental representation of social connections generalizes across a broad range of diversities, the primacy given one over other dimensions of the social experience may change over the life course, such that feelings of loneliness may be more strongly influenced by, for instance, Relational than Collective Connectedness at some stages of the life course, and vice versa for other groups and at other life stages. These are questions that will require longitudinal data to address.

#### Conclusion

Urban Chinese and urban American young and older adults inhabit distinct cultural worlds but the mental representation of their social worlds is quite similar. The particulars of life in each culture and in each generation give rise to some differences in the sources of social fulfilment in the intimate, relational, and collective aspects of social life, but the same three dimensions are relevant across cultures and across generations within the Chinese culture. This study provides the first evidence of the generality of people's mental representations of their social connections across two diverse societies. Our common humanity consists in large part of the intimate, relational, and collective social identities we each hold, and it seems reasonable to expect that a three-dimensional mental representation of our social connections will extend to other cultures and societies both now and in the future.

## Supporting Information

Figure S1 Multi-dimensional scaling analysis of Revised UCLA Loneliness Scale items in U.S. young adults (N = 135).
(PDF)

Figure S2 Multi-dimensional scaling analysis of Revised UCLA Loneliness Scale items in U.S. older adults (N = 229).
(PDF)

Figure S3 Multi-dimensional scaling analysis of Revised UCLA Loneliness Scale items in Beijing young adults (N = 267).
(PDF)

Figure S4 Multi-dimensional scaling analysis of Revised UCLA Loneliness Scale items in Beijing older adults (N = 246).
(PDF)

Table S1 Correlation matrix of Revised UCLA Loneliness Scale items in Beijing young adults (N = 266).
(DOCX)

Table S2 Correlation matrix of Revised UCLA Loneliness Scale items in Chinese middle-aged adults (N = 246).
(DOCX)
