# Supplementary material for: The Mental Representation of Social Connections: Generalizability Extended to Beijing Adults
Source: PLoS One. 2012 Sep 11;7(9):e44065. doi: 10.1371/journal.pone.0044065 (PMC3442957; doi:10.1371/journal.pone.0044065)
Supplement: Figure S1 — Multi-dimensional scaling analysis of Revised UCLA Loneliness Scale items in U.S. young adults (N = 135). (PDF) [file pone.0044065.s001.pdf]

Notes

Output Created		13-Feb-2012 16:23:08
Comments		
Input	Data	C:\Users\Louise Hawkley\Documents\lhawkley\MyPubs\Beijing Collaboration_UCLA Factor Analyses\UCLA_US Younger Adults.sav
	Active Dataset	DataSet3
	Filter	<none>
	Weight	<none>
	Split File	<none>
	N of Rows in Working Data File	135
Syntax		ALSCAL<br><br>/MATRIX=IN('C:\Users\LOUISE~1\AppData\Local\Temp\spss376\spssalsc.tmp')<br>/LEVEL=ORDINAL<br>/CONDITION=MATRIX<br>/MODEL=EUCLID<br>/CRITERIA=CONVERGE(0.001) STRESSMIN(0.005) ITER(30) CUTOFF(0) DIMENS(2,3)<br>/PLOT=DEFAULT ALL<br>/PRINT=HEADER.
Resources	Processor Time	00 00:00:02.169
	Elapsed Time	00 00:00:02.121

Derived Stimulus Configuration

Euclidean distance model

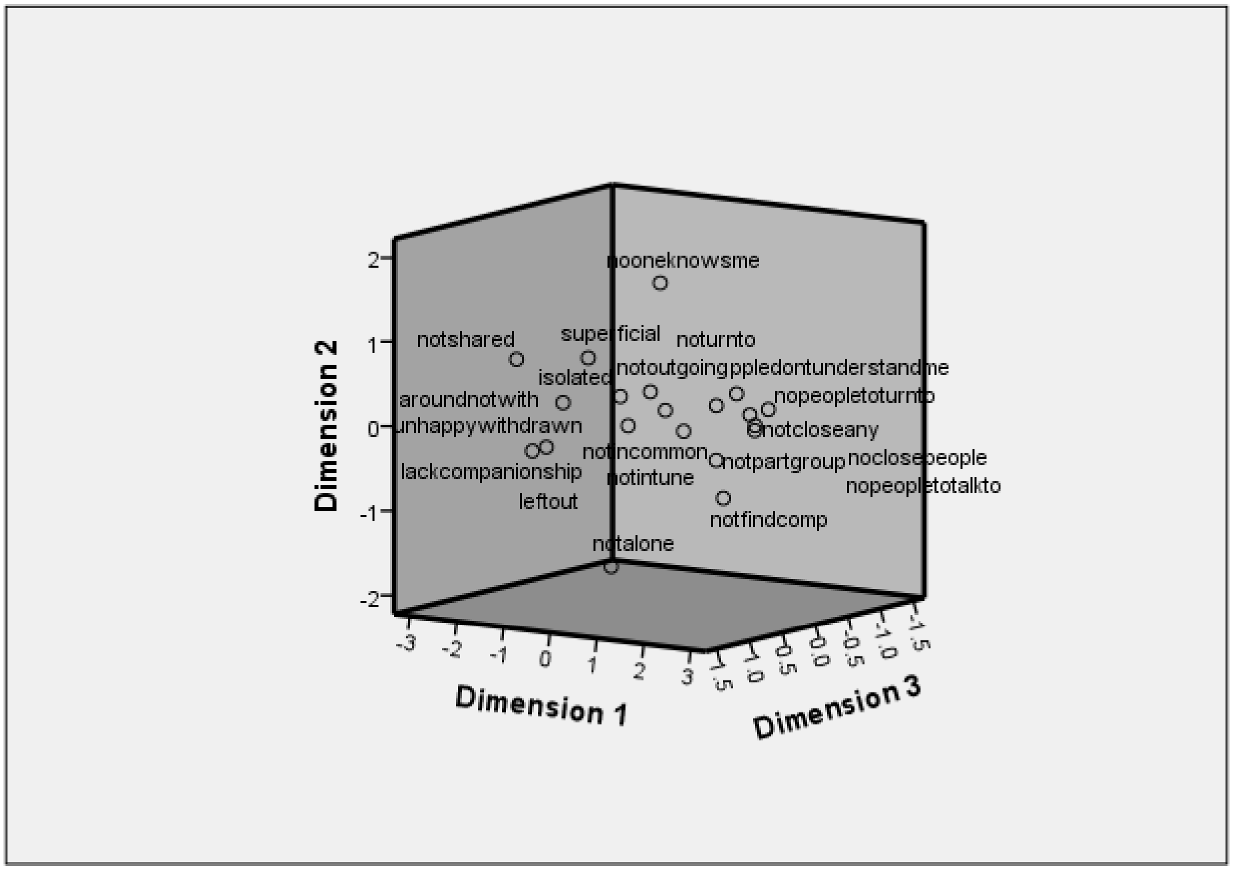

Derived Stimulus Configuration

Euclidean distance model

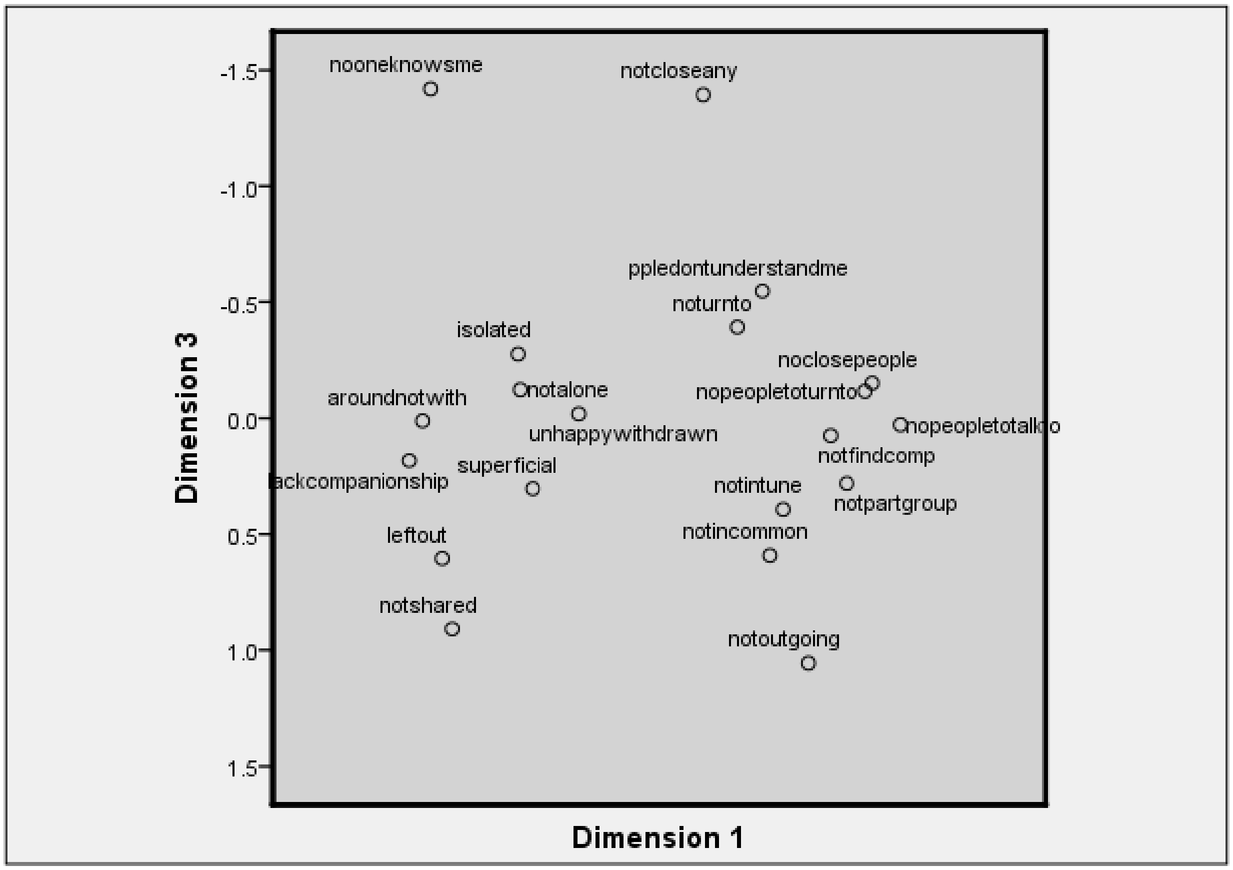

Derived Stimulus Configuration

Euclidean distance model

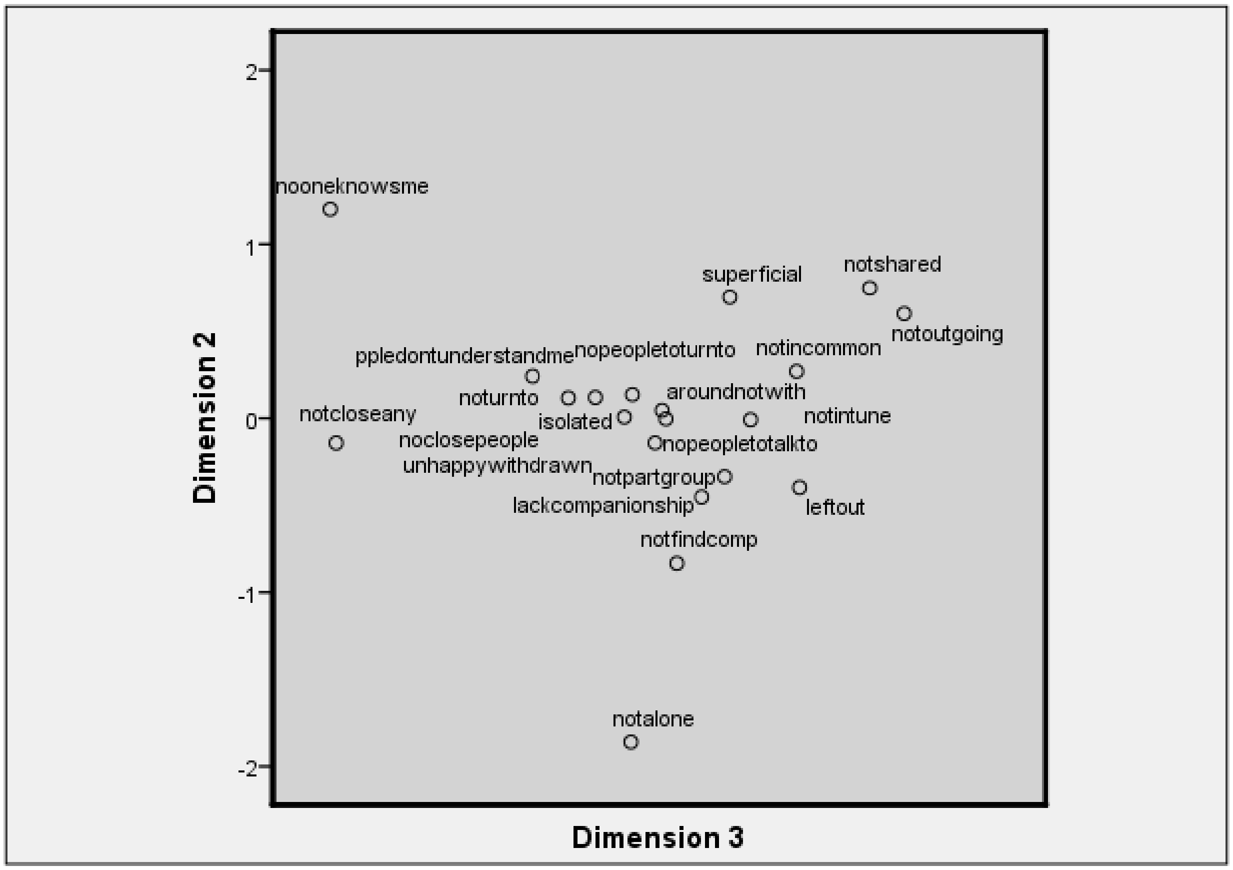

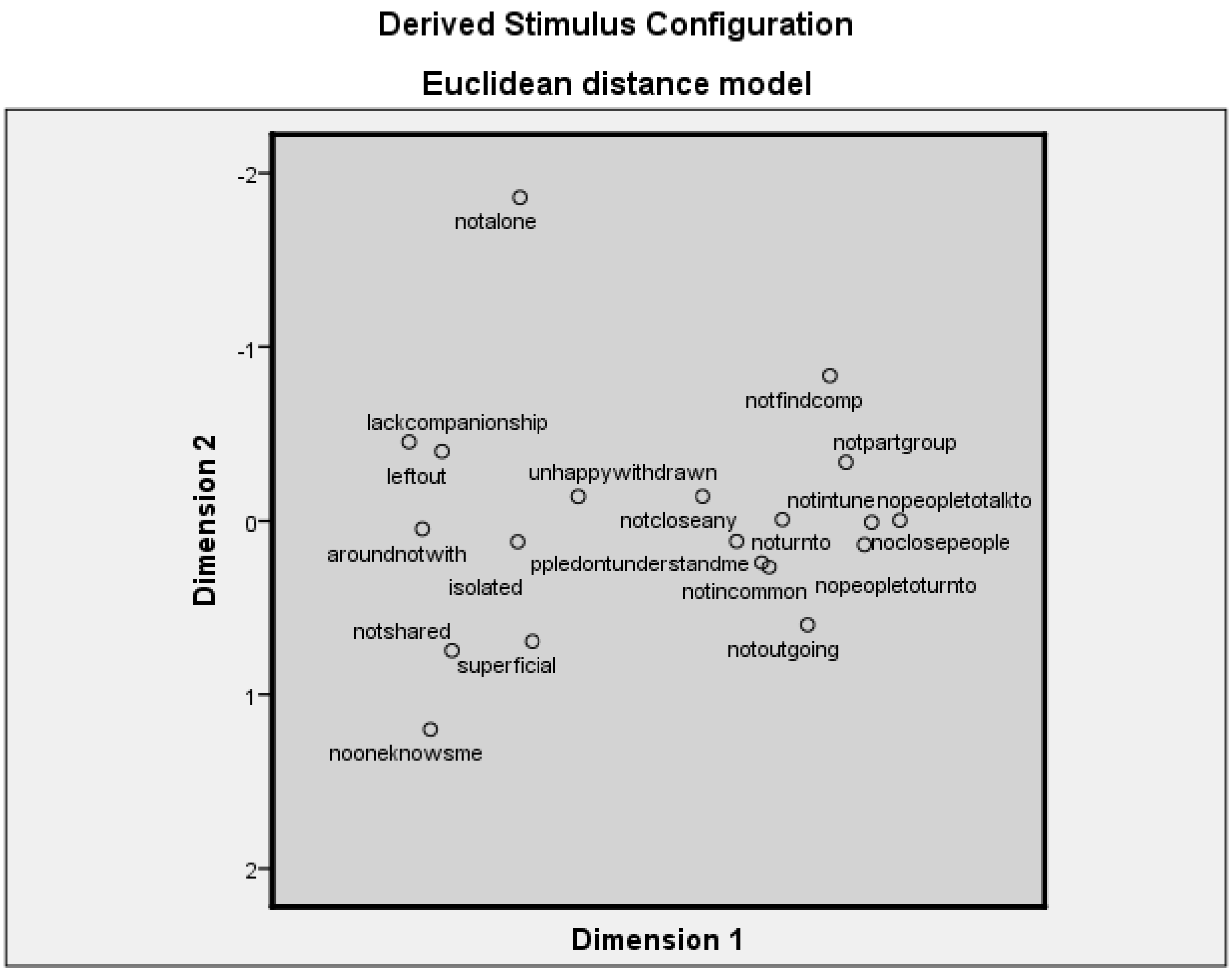
Derived Stimulus Configuration
Euclidean distance model
Dimension 2
Dimension 1
-2
-1
0
1
2
notalone
notfindcomp
lackcompanionship
notpartgroup
leftout
unhappywithdrawn
notintune
nopeopletotalkto
notcloseany
aroundnotwith
noturnto
noclosepeople
ppledontunderstandme
isolated
notincommon
nopeopletoturnto
notshared
notoutgoing
superficial
nooneknowsme

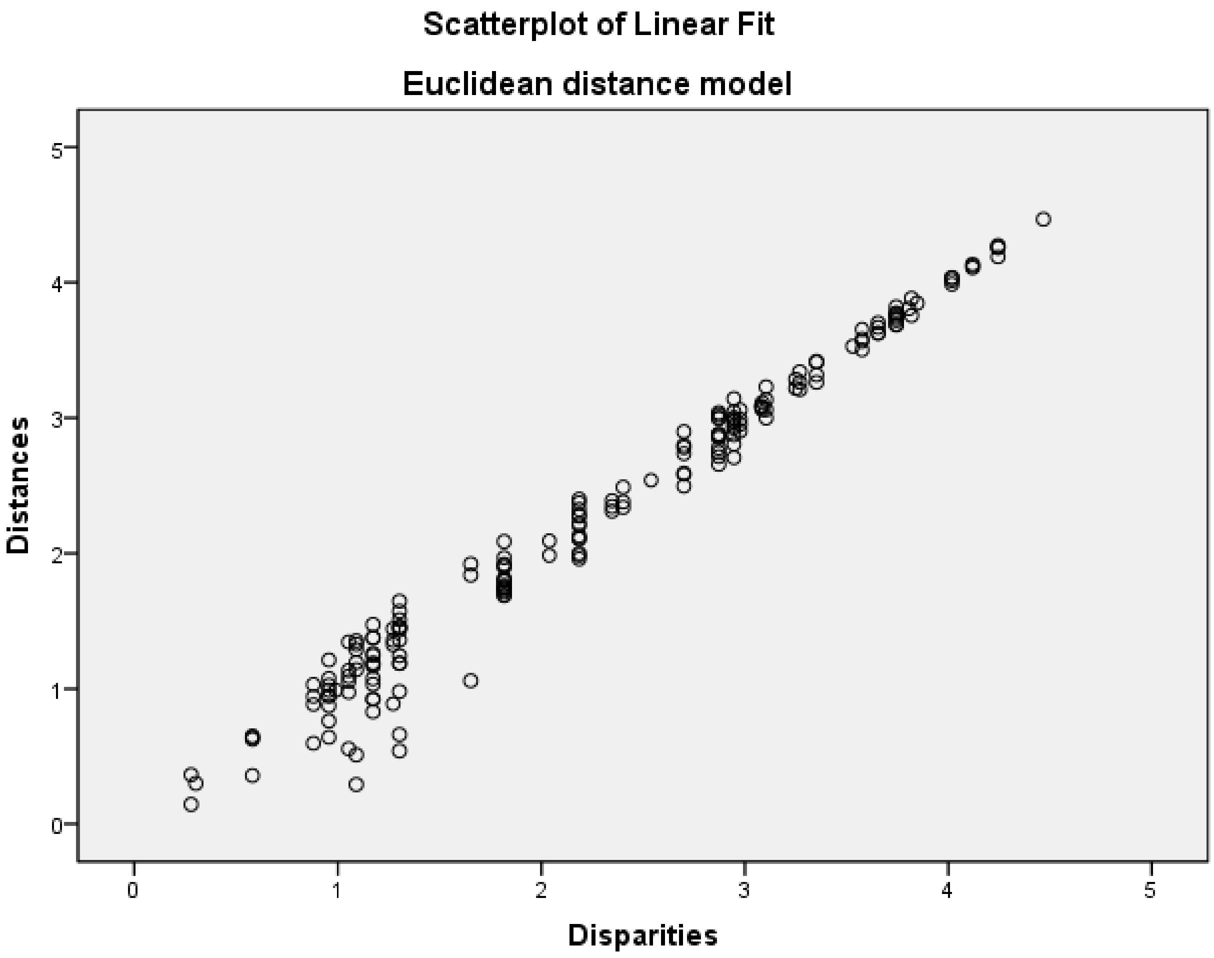

Scatterplot of Linear Fit
Euclidean distance model
Distances
Disparities
0
1
2
3
4
5

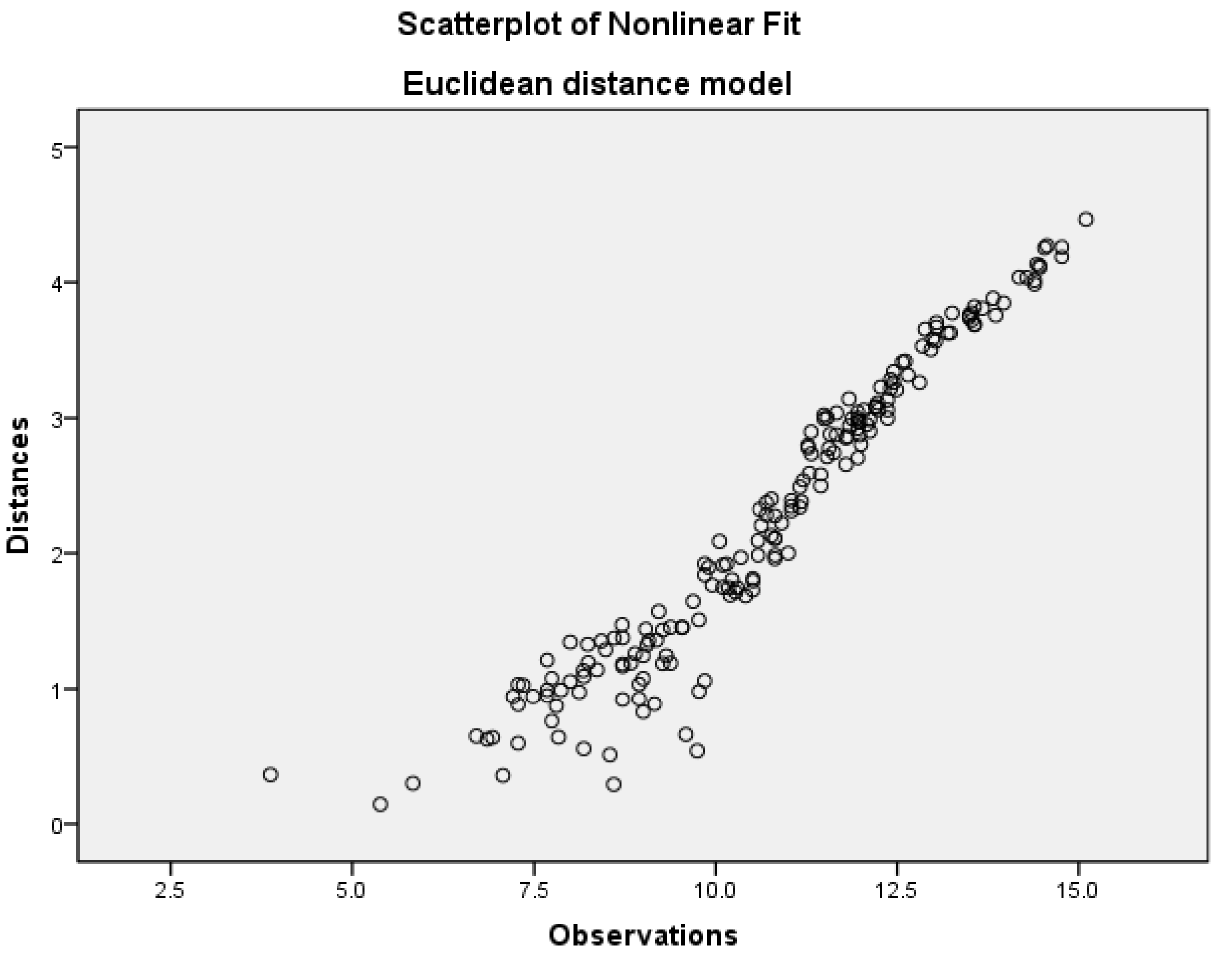
Scatterplot of Nonlinear Fit
Euclidean distance model
Distances
0
1
2
3
4
5
2.5
5.0
7.5
10.0
12.5
15.0
Observations

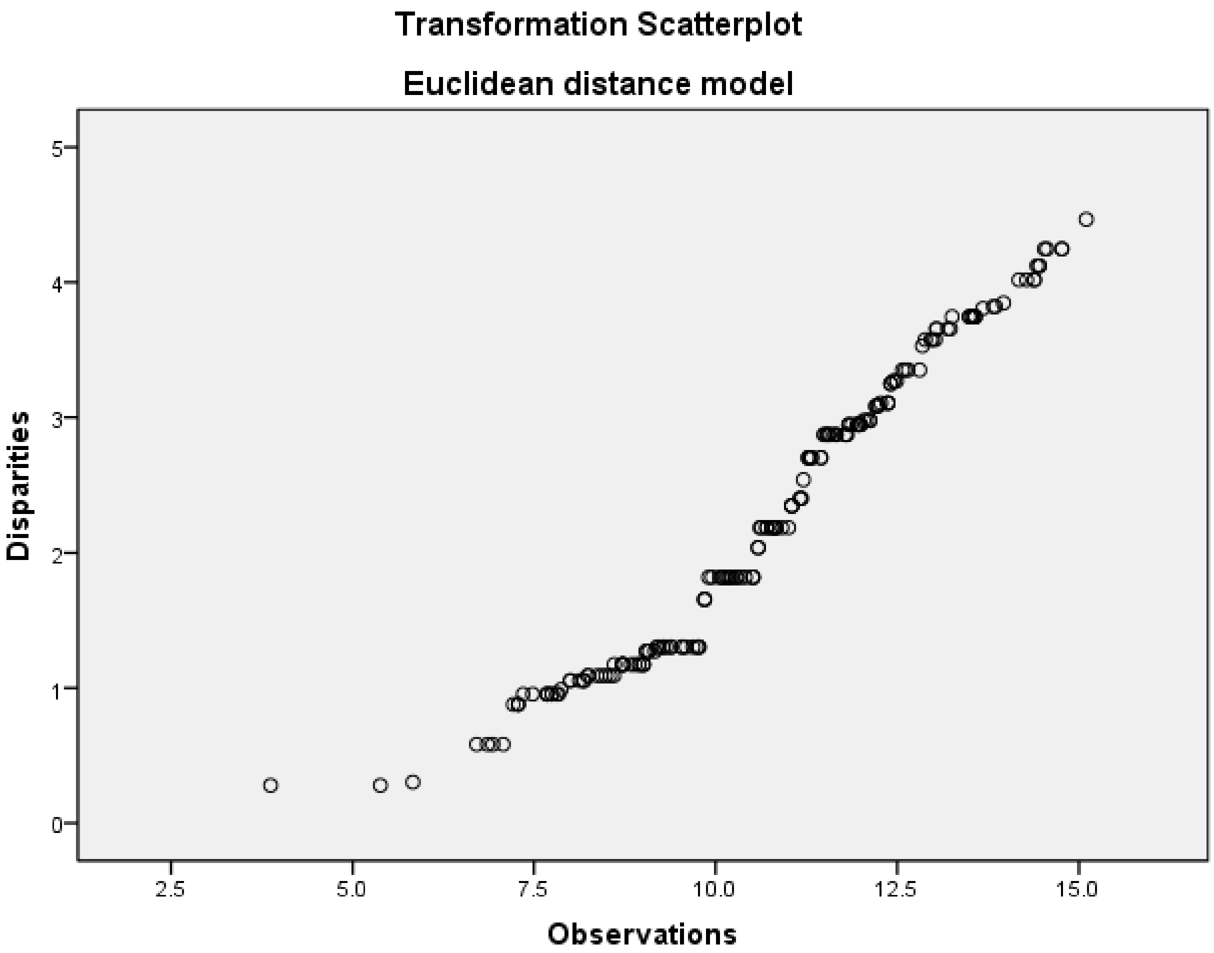
Transformation Scatterplot
Euclidean distance model
Disparities
Observations
0
1
2
3
4
5
2.5
5.0
7.5
10.0
12.5
15.0

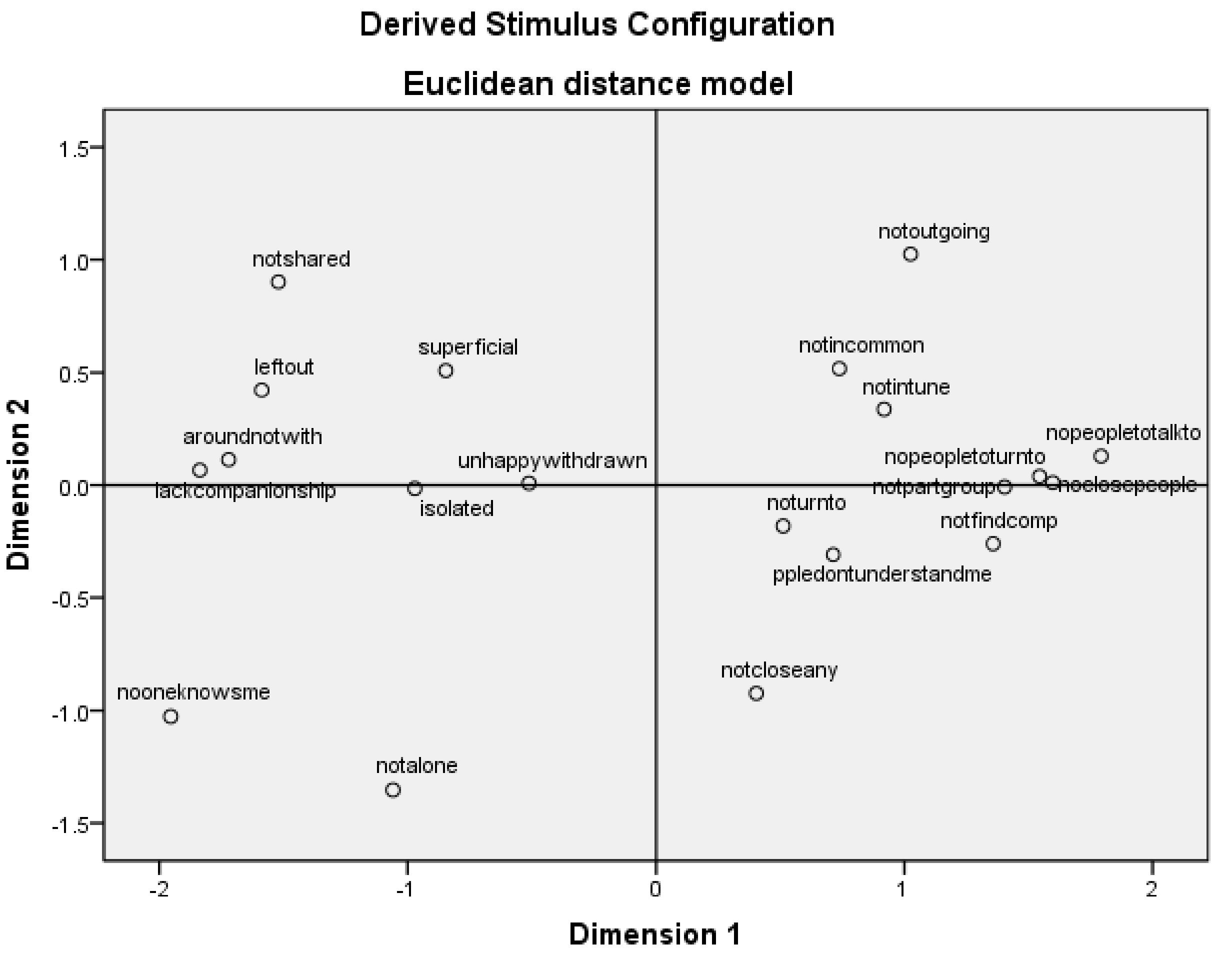

Derived Stimulus Configuration
Euclidean distance model
Dimension 2
Dimension 1
1.5
1.0
0.5
0.0
-0.5
-1.0
-1.5
-2
-1
0
1
2
notshared
superficial
leftout
aroundnotwith
lackcompanionship
isolated
unhappywithdrawn
nooneknowsme
notalone
notoutgoing
notincommon
notintune
nopeopletotalkto
nopeopletoturnto
notpartgroup
nocloseppeople
noturnto
notfindcomp
ppledontunderstandme
notcloseany

Scatterplot of Linear Fit

Euclidean distance model

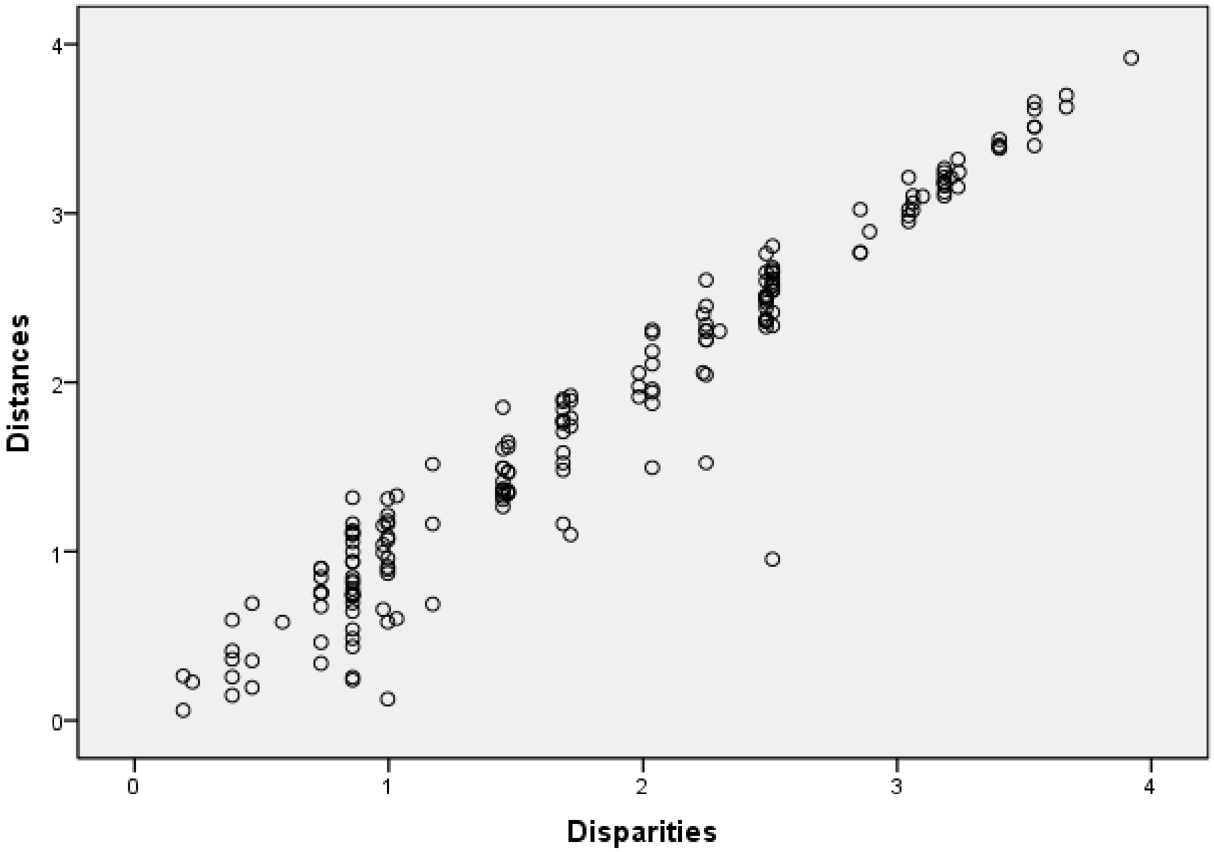

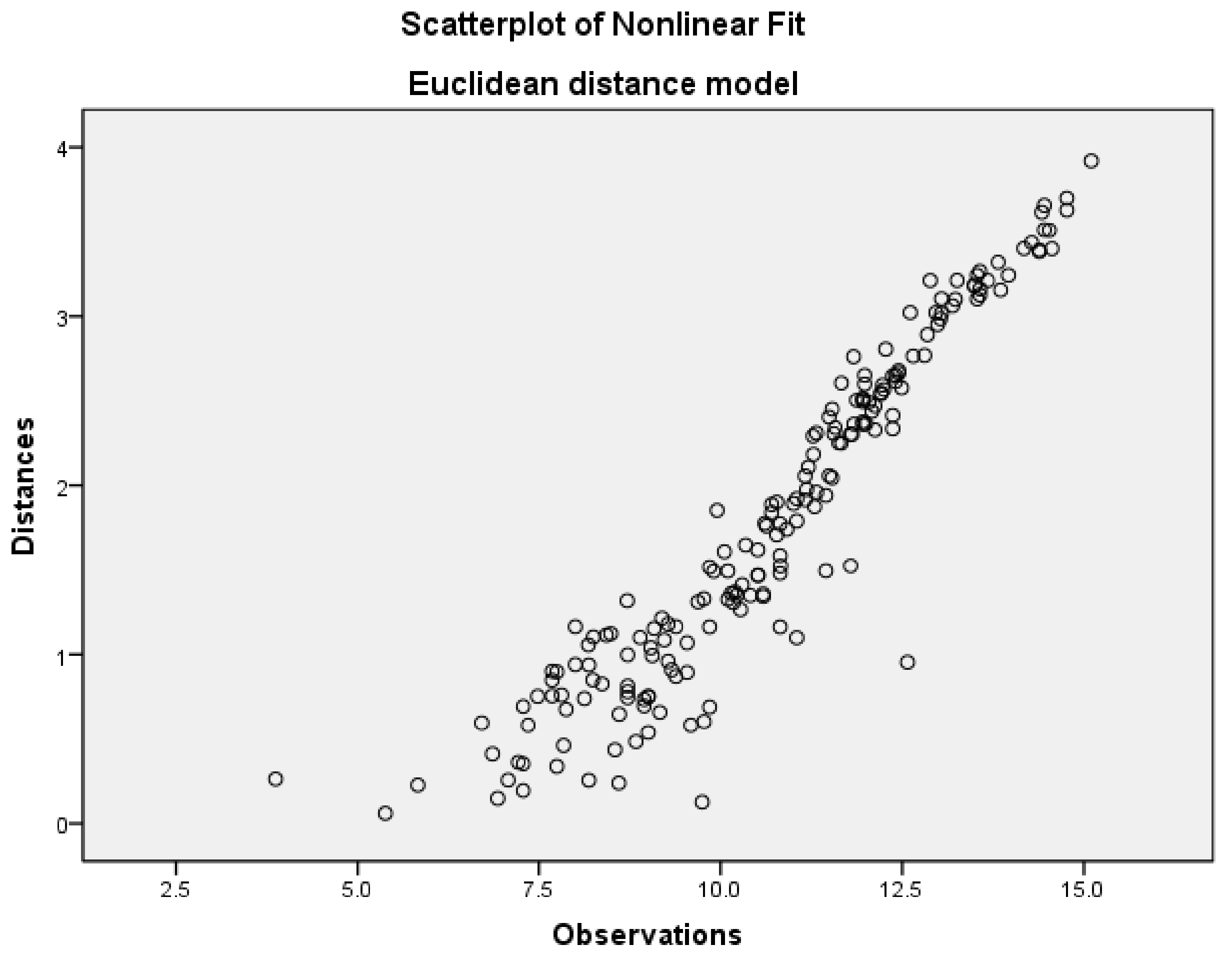

Scatterplot of Nonlinear Fit
Euclidean distance model
Distances
Observations
0
1
2
3
4
2.5
5.0
7.5
10.0
12.5
15.0

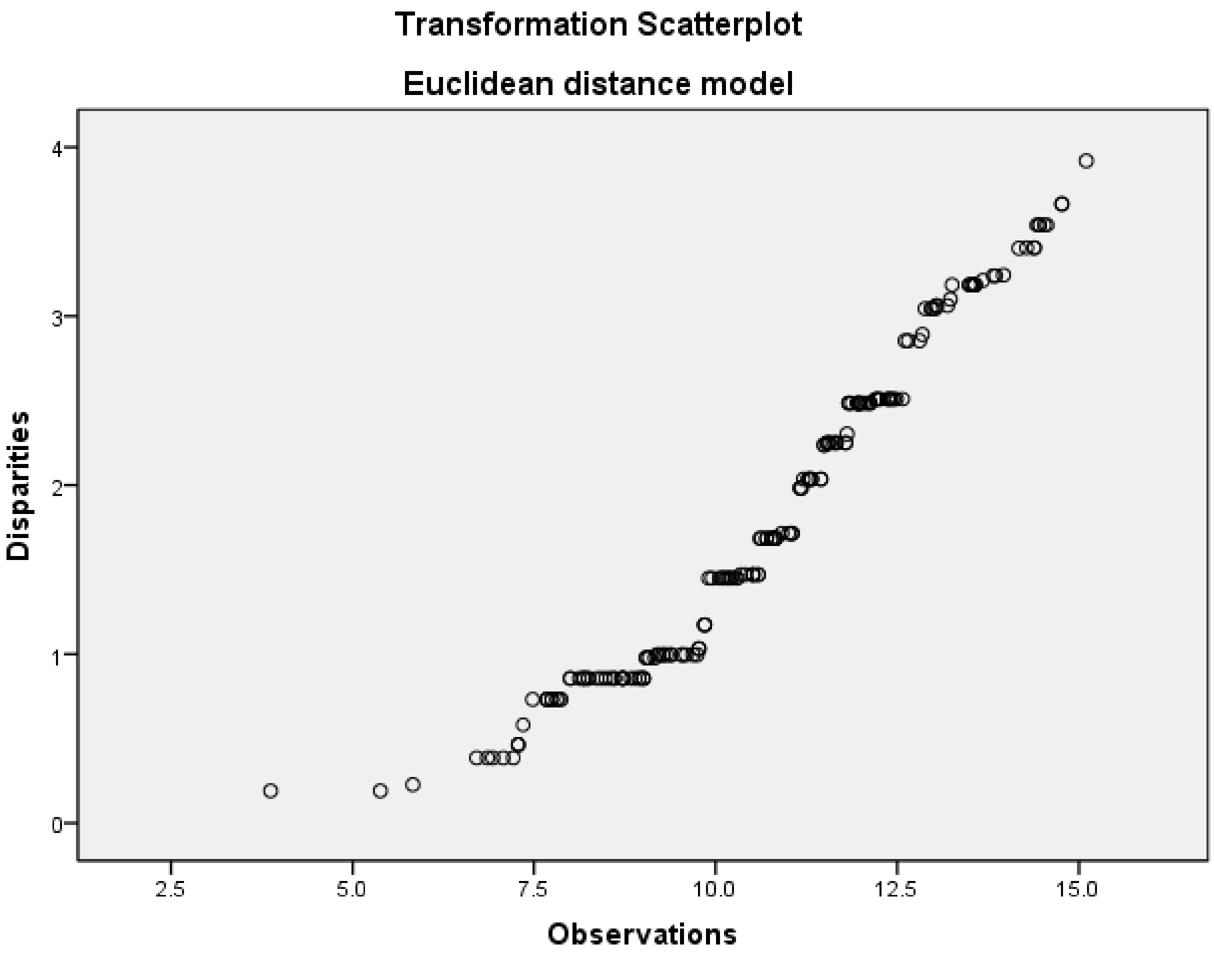
Transformation Scatterplot
Euclidean distance model
Disparities
0
1
2
3
4
2.5
5.0
7.5
10.0
12.5
15.0
Observations

Alscal Procedure Options

Data Options-

Number of Rows (Observations/Matrix).	20
Number of Columns (Variables) . . .	20
Number of Matrices	1
Measurement Level	Ordinal
Data Matrix Shape	Symmetric
Type	Dissimilarity
Approach to Ties	Leave Tied
Conditionality	Matrix
Data Cutoff at	.000000

Model Options-

Model	Euclid
Maximum Dimensionality	3
Minimum Dimensionality	2
Negative Weights	Not Permitted

Output Options-

Job Option Header	Printed
Data Matrices	Not Printed
Configurations and Transformations .	Plotted
Output Dataset	Not Created
Initial Stimulus Coordinates . . .	Computed

Algorithmic Options-

Maximum Iterations	30
Convergence Criterion	.00100
Minimum S-stress	.00500
Missing Data Estimated by	Ulbounds
Tiestore	190

Iteration history for the 3 dimensional solution (in squared distances)

Young's S-stress formula 1 is used.

Iteration	S-stress	Improvement
1	.09984	
2	.06869	.03116
3	.06336	.00533
4	.06173	.00163
5	.06115	.00058

Iterations stopped because
S-stress improvement is less than .001000

Stress and squared correlation (RSQ) in distances

RSQ values are the proportion of variance of the scaled data (disparities)
in the partition (row, matrix, or entire data) which
is accounted for by their corresponding distances.
Stress values are Kruskal's stress formula 1.

For matrix
Stress = .06764 RSQ = .97607

Configuration derived in 3 dimensions
Stimulus Coordinates

		Dimension		
Stimulus Number	Stimulus Name	1	2	3
1	notintun	1.0677	-.0083	.3938
2	lackcomp	-2.1559	-.4537	.1830
3	noturnto	.6736	.1164	-.3920
4	notalone	-1.2009	-1.8603	-.1226
5	notpartg	1.6158	-.3372	.2820
6	notincom	.9535	.2688	.5914
7	notclose	.3799	-.1414	-1.3942
8	notshare	-1.7859	.7481	.9082
9	notoutgo	1.2850	.6005	1.0564
10	noclosep	1.8376	.0072	-.1510
11	leftout	-1.8722	-.3994	.6045
12	superfic	-1.0928	.6955	.3045
13	noonekno	-1.9713	1.2009	-1.4196
14	isolated	-1.2197	.1192	-.2759

15	notfindc	1.4769	-.8339	.0748
16	ppledont	.8891	.2412	-.5472
17	unhappyw	-.6944	-.1409	-.0187
18	aroundno	-2.0411	.0454	.0111
19	nopeople	2.0802	-.0036	.0276
20	nopeop_1	1.7751	.1356	-.1162

Iteration history for the 2 dimensional solution (in squared distances)

Young's S-stress formula 1 is used.

Iteration	S-stress	Improvement
1	.18812	
2	.13030	.05781
3	.12515	.00515
4	.12365	.00150
5	.12294	.00071

Iterations stopped because
S-stress improvement is less than .001000

Stress and squared correlation (RSQ) in distances

RSQ values are the proportion of variance of the scaled data (disparities)
in the partition (row, matrix, or entire data) which
is accounted for by their corresponding distances.
Stress values are Kruskal's stress formula 1.

For matrix
Stress = .11252 RSQ = .94475

Configuration derived in 2 dimensions
Stimulus Coordinates
Dimension

Stimulus Number	Stimulus Name	1	2
1	notintun	.9185	.3358
2	lackcomp	-1.8375	.0661
3	noturnto	.5114	-.1808
4	notalone	-1.0584	-1.3536
5	notpartg	1.4041	-.0082
6	notincom	.7383	.5176

7	notclose	.4037	-.9238
8	notshare	-1.5201	.9013
9	notoutgo	1.0247	1.0241
10	noclosep	1.5974	.0107
11	leftout	-1.5869	.4220
12	superfic	-.8464	.5089
13	noonekno	-1.9541	-1.0262
14	isolated	-.9706	-.0148
15	notfindc	1.3574	-.2593
16	ppledont	.7129	-.3079
17	unhappyw	-.5104	.0084
18	aroundno	-1.7198	.1125
19	nopeople	1.7913	.1281
20	nopeop_1	1.5445	.0389

Abbreviated Name	Extended Name
aroundno	aroundnotwith
lackcomp	lackcompanionship
noclosep	noclosepeople
noonekno	nooneknowsme
nopeop_1	nopeopletoturnto
nopeople	nopeopletotalkto
notclose	notcloseany
notfindc	notfindcomp
notincom	notincommon
notintun	notintune
notoutgo	notoutgoing
notpartg	notpartgroup
notshare	notshared
ppledont	ppledontunderstandme
superfic	superficial
unhappyw	unhappywithdrawn
