# Supplementary material for: The Mental Representation of Social Connections: Generalizability Extended to Beijing Adults
Source: PLoS One. 2012 Sep 11;7(9):e44065. doi: 10.1371/journal.pone.0044065 (PMC3442957; doi:10.1371/journal.pone.0044065)
Supplement: Figure S2 — Multi-dimensional scaling analysis of Revised UCLA Loneliness Scale items in U.S. older adults (N = 229). (PDF) [file pone.0044065.s002.pdf]

**Notes**

| | | |
|---|---|---|
| Output Created | | 13-Feb-2012 16:24:43 |
| Comments | | |
| Input | Data | C:\Users\Louise Hawkley\Documents\lhawkley\MyPubs\Beijing Collaboration_UCLA Factor Analyses\UCLA_US Older Adults_reverse-coded.sav |
| | Active Dataset | DataSet4 |
| | Filter | <none> |
| | Weight | <none> |
| | Split File | <none> |
| | N of Rows in Working Data File | 229 |
| Syntax | | ALSCAL<br><br>/MATRIX=IN('C:\Users\LOUISE~1\AppData\Local\Temp\spss376\spssalsc.tmp')<br>/LEVEL=ORDINAL<br>/CONDITION=MATRIX<br>/MODEL=EUCLID<br>/CRITERIA=CONVERGE(0.001) STRESSMIN(0.005) ITER(30) CUTOFF(0) DIMENS(2,3)<br>/PLOT=DEFAULT ALL<br>/PRINT=DATA HEADER. |
| Resources | Processor Time | 00 00:00:02.106 |
| | Elapsed Time | 00 00:00:02.090 |

## Derived Stimulus Configuration

## Euclidean distance model

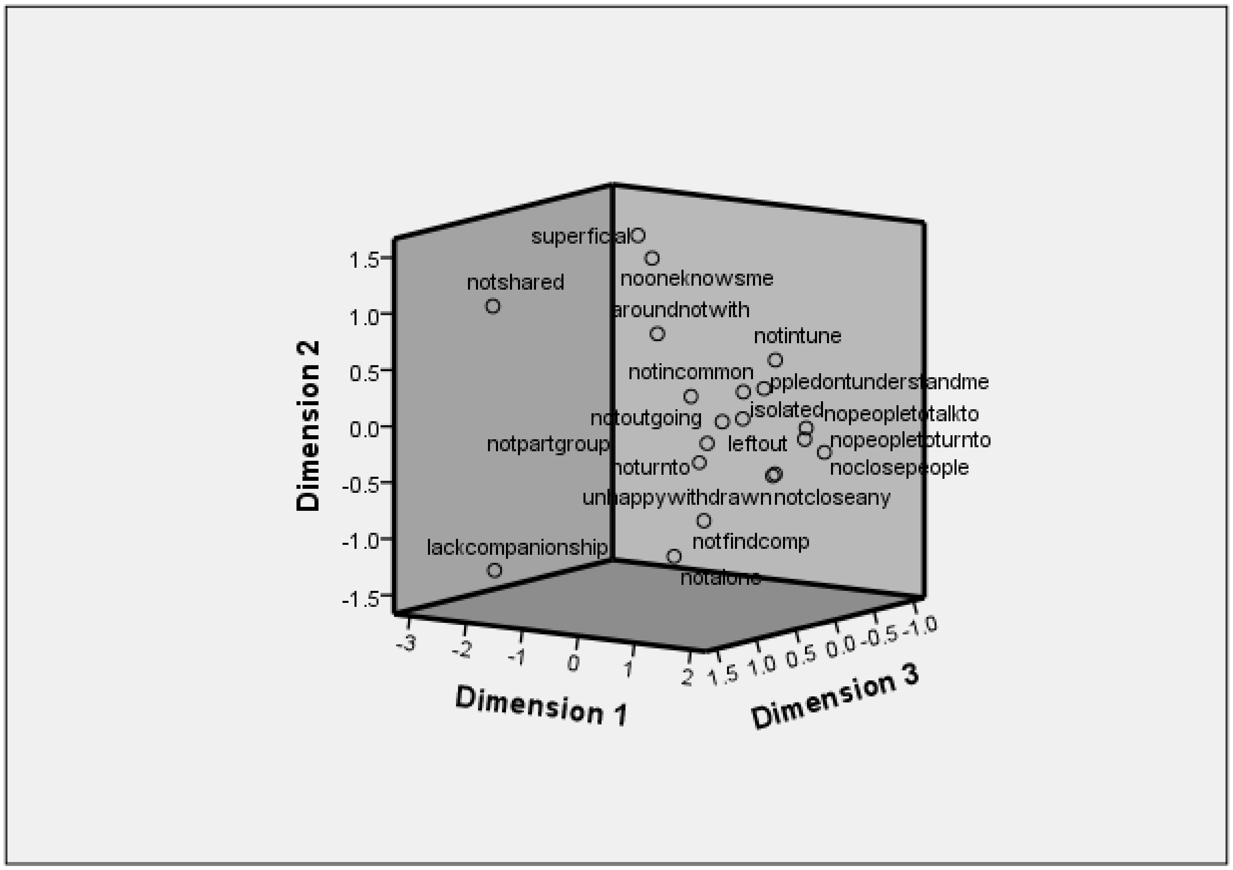

## Derived Stimulus Configuration

## Euclidean distance model

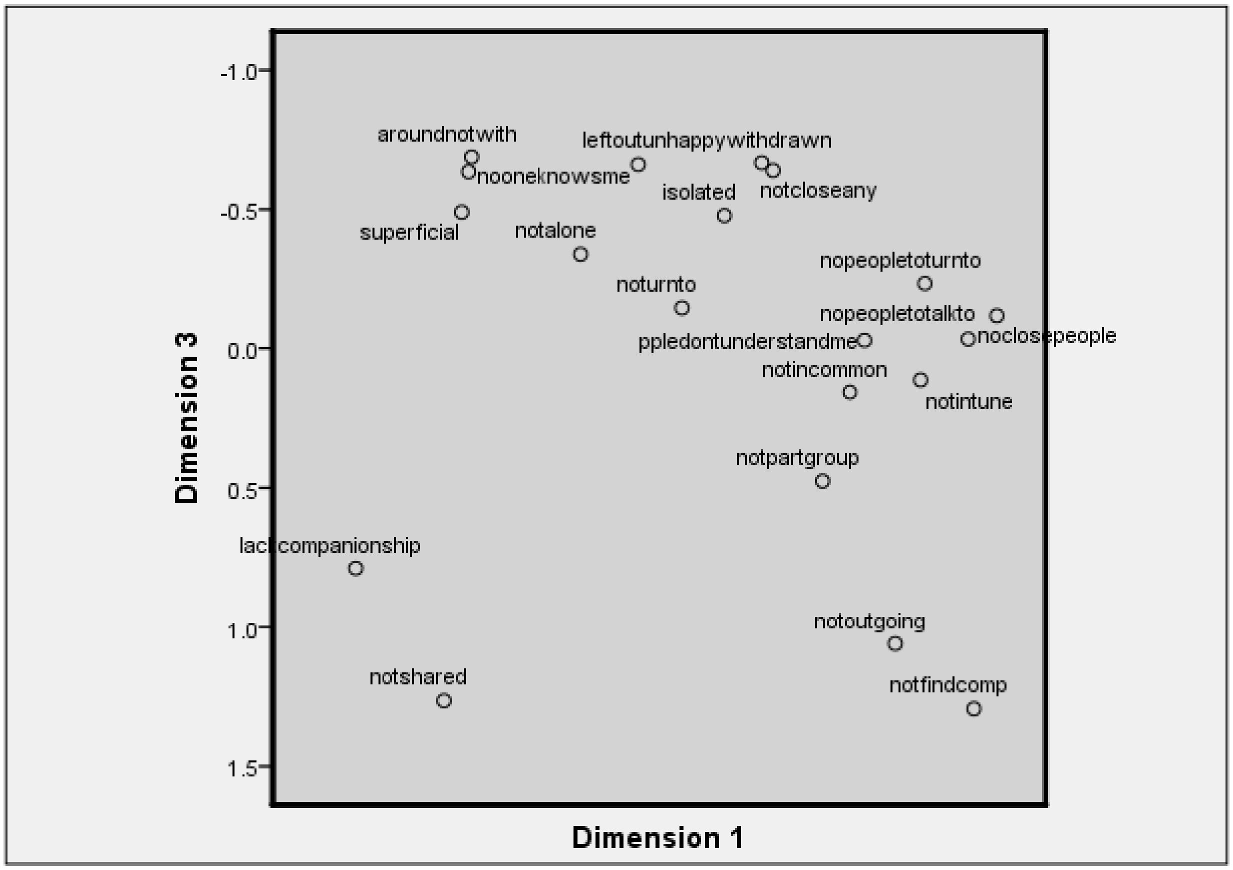

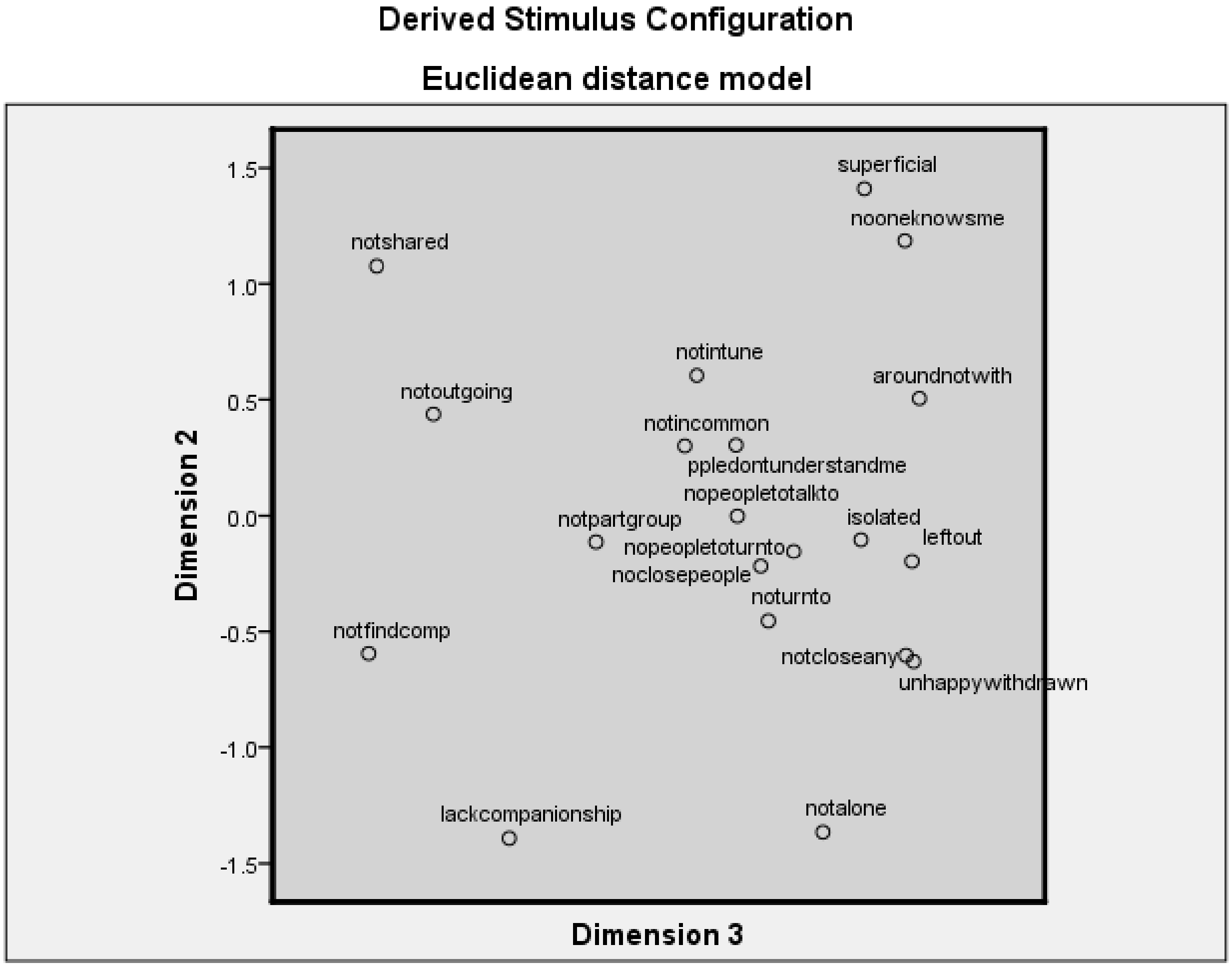
Derived Stimulus Configuration
Euclidean distance model
Dimension 2
Dimension 3
1.5
1.0
0.5
0.0
-0.5
-1.0
-1.5
superficial
nooneknowsme
notshared
notintune
aroundnotwith
notoutgoing
notincommon
ppledontunderstandme
nopeopletotalkto
notpartgroup
isolated
leftout
nopeopletoturnto
noclosepeople
noturnto
notfindcomp
notcloseany
unhappywithdrawn
lackcompanionship
notalone

**Derived Stimulus Configuration**

**Euclidean distance model**

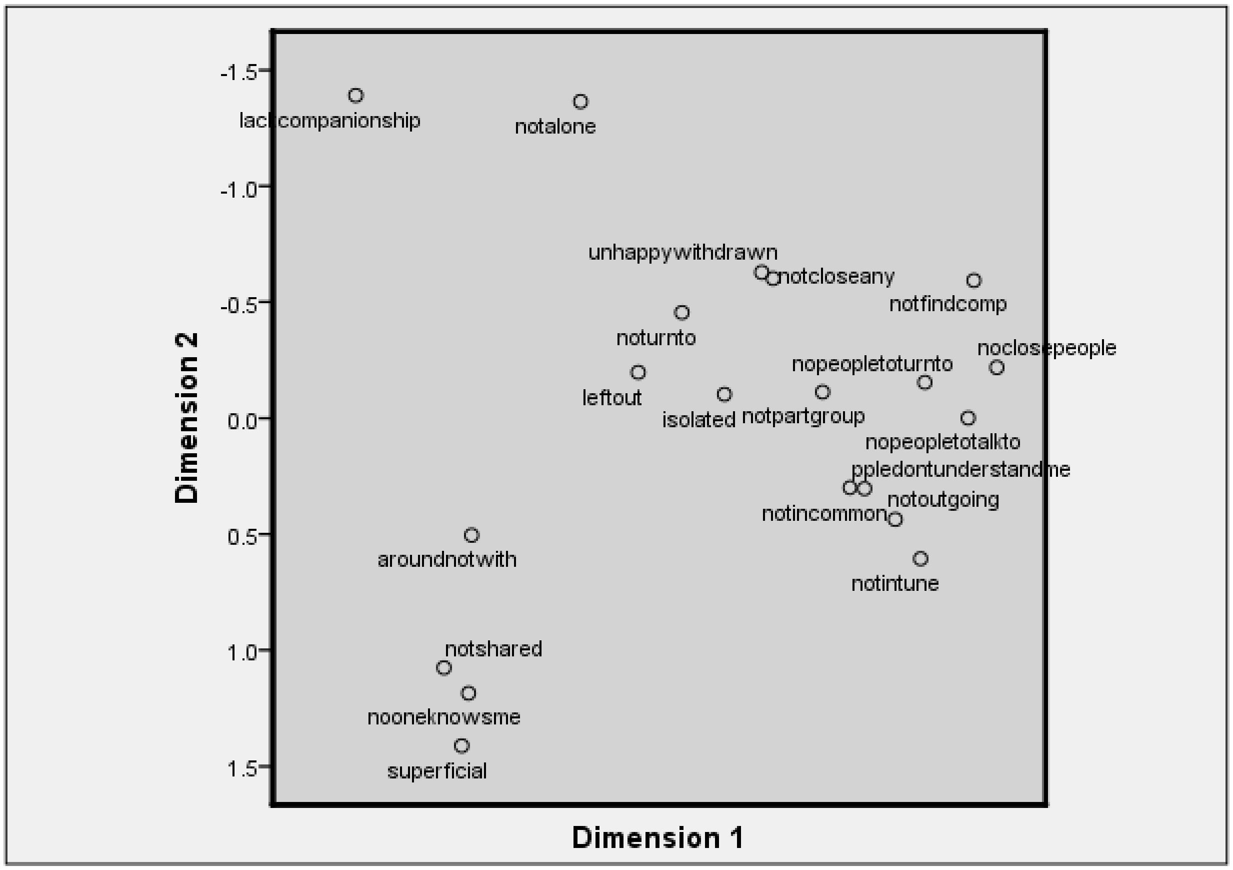

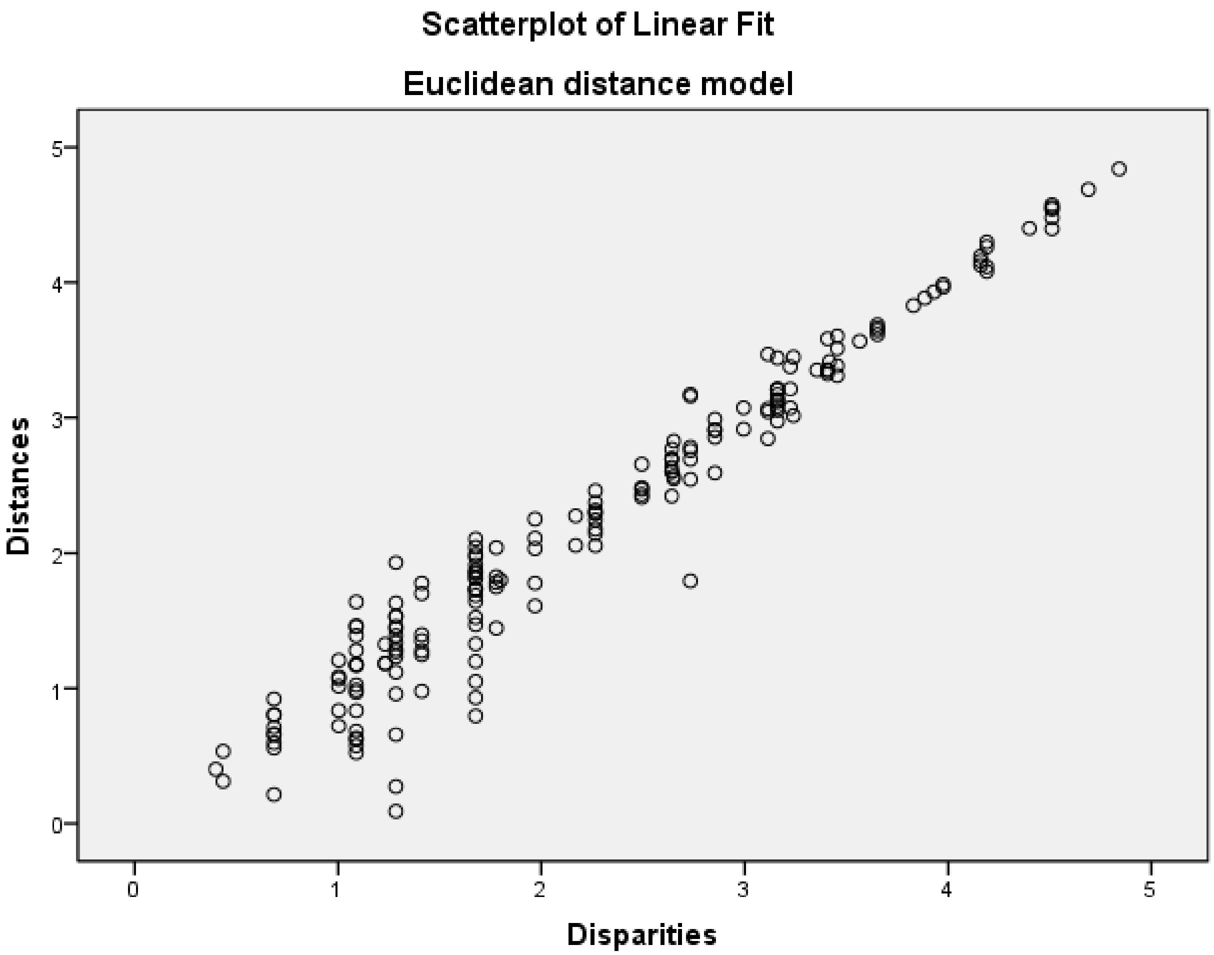
Scatterplot of Linear Fit
Euclidean distance model
Distances
Disparities
0
1
2
3
4
5
0
1
2
3
4
5

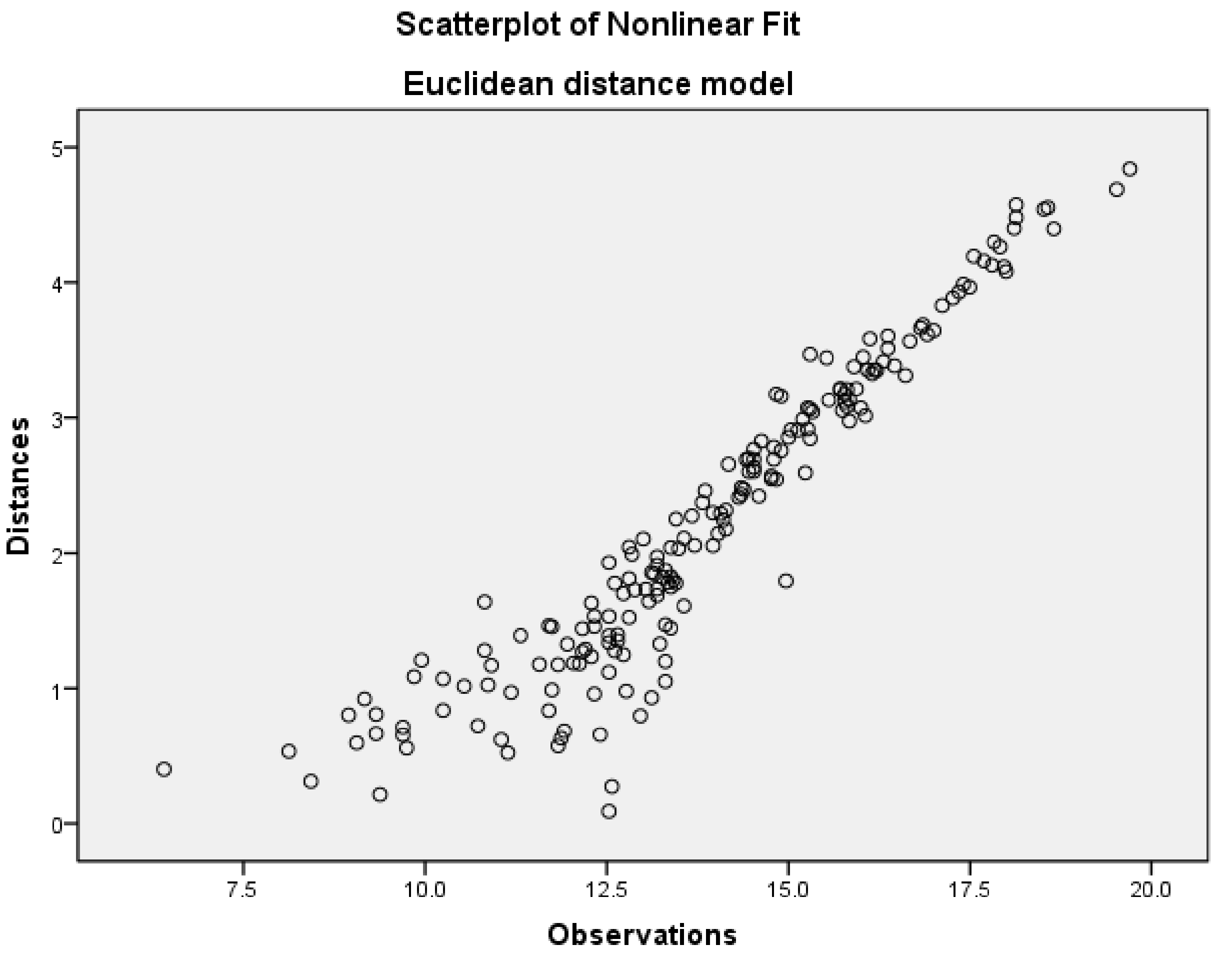
Scatterplot of Nonlinear Fit
Euclidean distance model
Distances
Observations
0
1
2
3
4
5
7.5
10.0
12.5
15.0
17.5
20.0

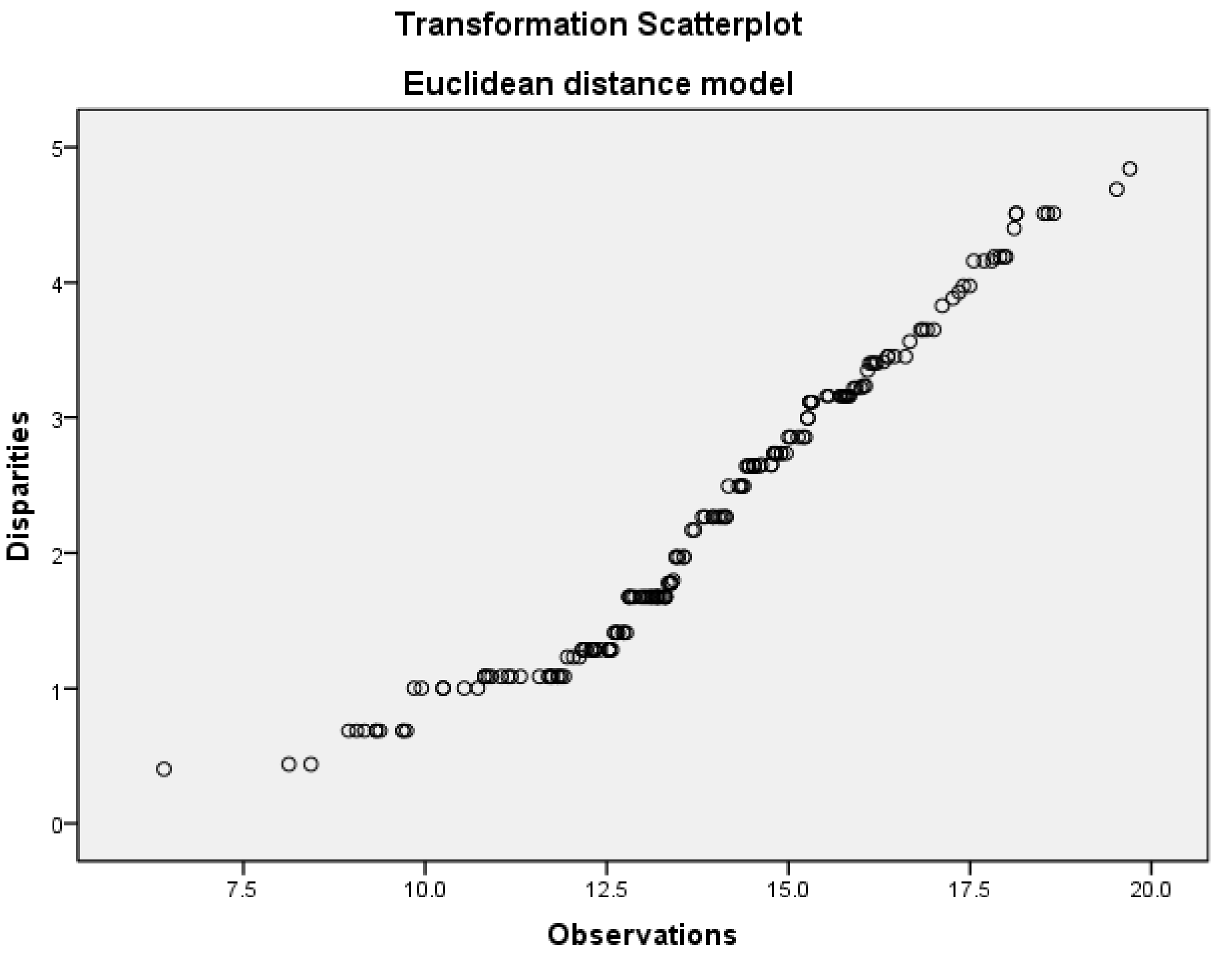
Transformation Scatterplot
Euclidean distance model
Disparities
0
1
2
3
4
5
7.5
10.0
12.5
15.0
17.5
20.0
Observations

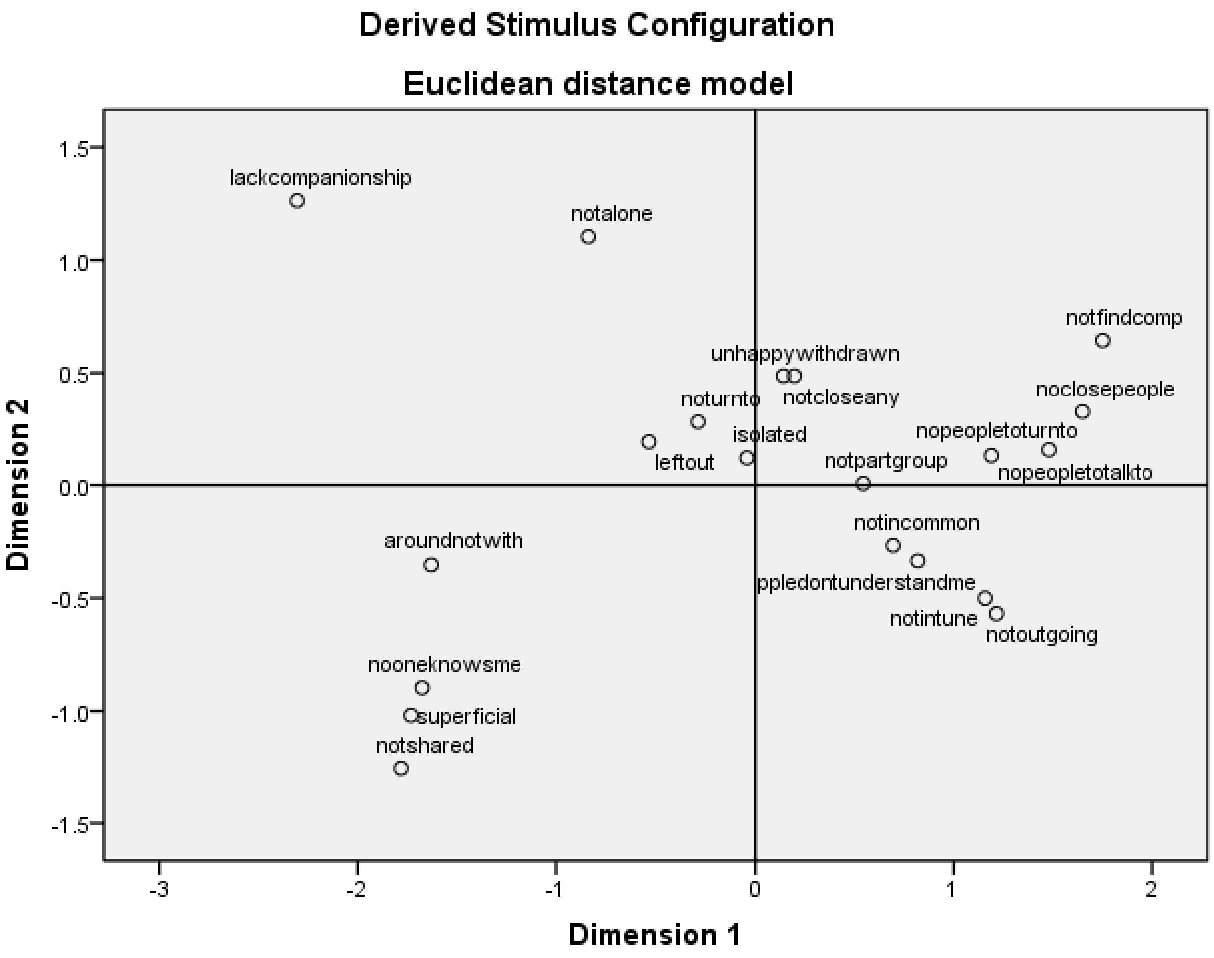

Derived Stimulus Configuration
Euclidean distance model
lackcompanionship
notalone
notfindcomp
unhappywithdrawn
notcloseany
noturnto
noclosepeople
isolated
nopeopletoturnto
leftout
notpartgroup
nopeopletotalkto
notincommon
aroundnotwith
ppledontunderstandme
notintune
notoutgoing
nooneknowsme
superficial
notshared
1.5
1.0
0.5
0.0
-0.5
-1.0
-1.5
-3
-2
-1
0
1
2
Dimension 1
Dimension 2

## Scatterplot of Linear Fit

## Euclidean distance model

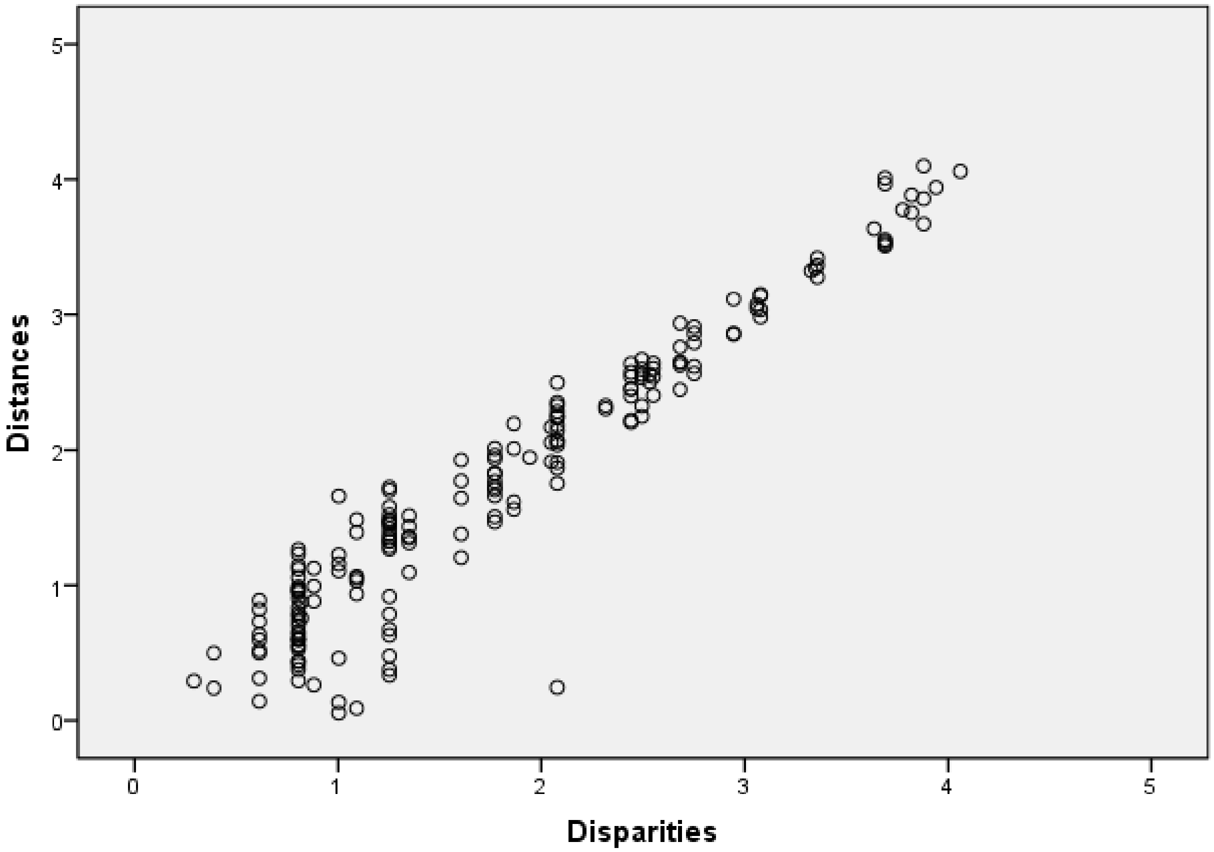

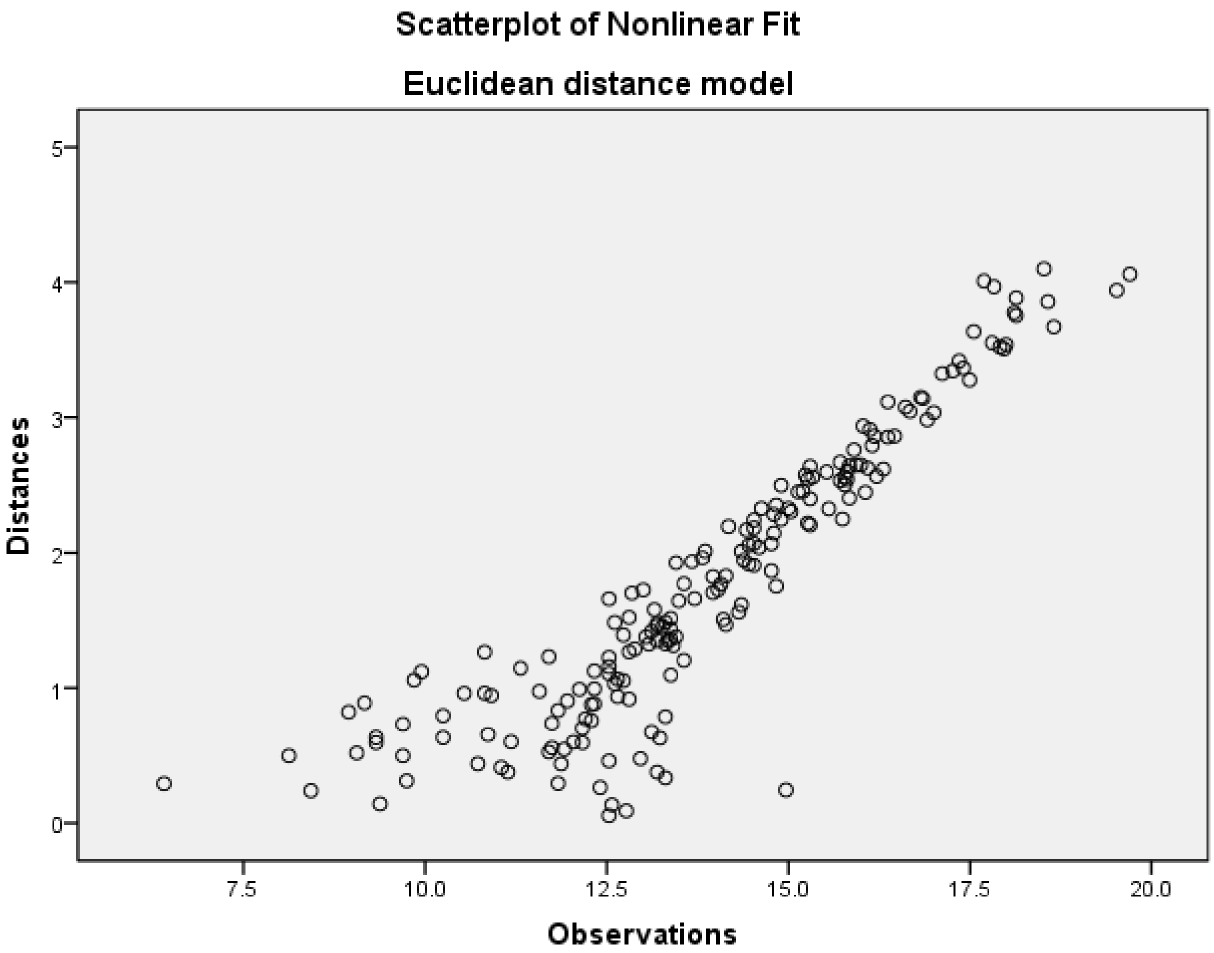
Scatterplot of Nonlinear Fit
Euclidean distance model
Distances
0
1
2
3
4
5
7.5
10.0
12.5
15.0
17.5
20.0
Observations

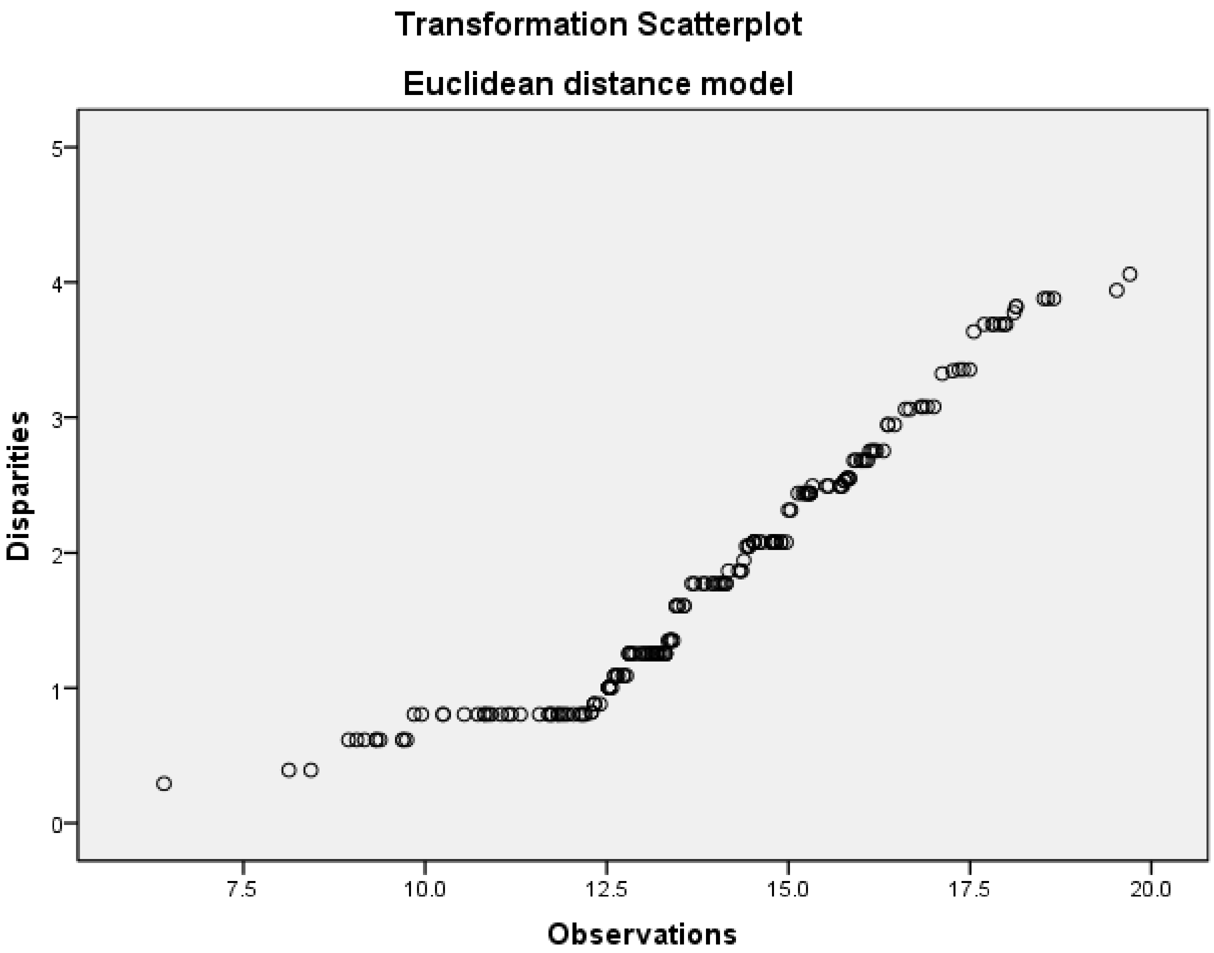
Transformation Scatterplot
Euclidean distance model
Disparities
Observations
0
1
2
3
4
5
7.5
10.0
12.5
15.0
17.5
20.0

Alscal Procedure Options

Data Options-

Number of Rows (Observations/Matrix). 20
Number of Columns (Variables) . . . . 20
Number of Matrices . . . . . . . 1
Measurement Level . . . . . . . . Ordinal
Data Matrix Shape . . . . . . . . Symmetric
Type . . . . . . . . . . . Dissimilarity
Approach to Ties . . . . . . . . Leave Tied
Conditionality . . . . . . . . Matrix
Data Cutoff at . . . . . . . . .000000

Model Options-

Model . . . . . . . . . . . Euclid
Maximum Dimensionality . . . . . 3
Minimum Dimensionality . . . . . 2
Negative Weights . . . . . . . Not Permitted

Output Options-

Job Option Header . . . . . . . Printed
Data Matrices . . . . . . . . Printed
Configurations and Transformations . Plotted
Output Dataset . . . . . . . . Not Created
Initial Stimulus Coordinates . . . Computed

Algorithmic Options-

Maximum Iterations . . . . . . 30
Convergence Criterion . . . . . .00100
Minimum S-stress . . . . . . . .00500
Missing Data Estimated by . . . . Ulbounds
Tiestore . . . . . . . . . . 190

Raw (unscaled) Data for Subject 1

| | 1 | 2 | 3 | 4 | 5 | 6 | 7 | 8 | 9 | 10 |
|---|---|---|---|---|---|---|---|---|---|---|
| 1 | .000 | | | | | | | | | |
| 2 | 18.138 | .000 | | | | | | | | |
| 3 | 13.491 | 14.526 | .000 | | | | | | | |
| 4 | 15.780 | 13.191 | 12.124 | .000 | | | | | | |
| 5 | 10.247 | 16.371 | 12.288 | 14.071 | .000 | | | | | |
| 6 | 9.055 | 17.407 | 12.329 | 14.457 | 9.747 | .000 | | | | |
| 7 | 13.454 | 16.310 | 11.705 | 13.565 | 12.166 | 11.958 | .000 | | | |
| 8 | 17.000 | 15.232 | 14.799 | 15.811 | 16.000 | 15.716 | 15.297 | .000 | | |
| 9 | 12.767 | 17.833 | 14.036 | 15.937 | 12.329 | 11.180 | 14.142 | 16.613 | .000 | |
| 10 | 10.536 | 19.698 | 13.675 | 15.811 | 11.314 | 9.950 | 13.191 | 18.111 | 12.329 | .000 |
| 11 | 14.142 | 14.526 | 12.410 | 10.817 | 13.379 | 13.416 | 13.304 | 14.457 | 14.526 | 14.526 |
| 12 | 16.031 | 14.832 | 14.387 | 15.033 | 14.900 | 15.264 | 16.062 | 14.967 | 16.912 | 17.550 |
| 13 | 16.462 | 15.748 | 13.964 | 14.422 | 15.297 | 15.199 | 14.629 | 13.191 | 16.125 | 18.000 |
| 14 | 13.191 | 15.716 | 11.832 | 10.817 | 12.042 | 11.832 | 11.874 | 15.264 | 13.379 | 12.845 |
| 15 | 12.884 | 18.520 | 14.765 | 16.093 | 13.379 | 12.728 | 14.318 | 17.692 | 13.304 | 13.304 |
| 16 | 11.136 | 17.972 | 12.806 | 14.177 | 10.724 | 9.381 | 12.610 | 15.906 | 12.530 | 9.849 |
| 17 | 13.115 | 16.217 | 12.961 | 12.530 | 13.229 | 12.649 | 12.530 | 15.524 | 14.107 | 13.379 |
| 18 | 16.155 | 14.832 | 13.304 | 13.711 | 15.297 | 15.000 | 14.353 | 12.806 | 16.371 | 17.263 |
| 19 | 9.695 | 19.519 | 13.565 | 15.780 | 10.909 | 9.165 | 13.077 | 17.804 | 12.207 | 8.426 |
| 20 | 10.247 | 18.655 | 12.610 | 14.900 | 10.863 | 9.327 | 12.728 | 17.493 | 12.166 | 8.124 |

| | 11 | 12 | 13 | 14 | 15 | 16 | 17 | 18 | 19 | 20 |
|---|---|---|---|---|---|---|---|---|---|---|
| 11 | .000 | | | | | | | | | |
| 12 | 13.964 | .000 | | | | | | | | |
| 13 | 13.153 | 12.570 | .000 | | | | | | | |
| 14 | 9.695 | 14.595 | 13.454 | .000 | | | | | | |
| 15 | 15.556 | 18.574 | 18.138 | 14.765 | .000 | | | | | |
| 16 | 13.266 | 15.843 | 15.330 | 11.576 | 13.342 | .000 | | | | |
| 17 | 11.747 | 15.843 | 14.799 | 11.045 | 14.353 | 12.649 | .000 | | | |
| 18 | 12.530 | 13.115 | 11.916 | 12.530 | 17.916 | 15.133 | 13.820 | .000 | | |
| 19 | 13.856 | 17.349 | 17.117 | 12.806 | 11.747 | 8.944 | 13.038 | 16.823 | .000 | |
| 20 | 13.000 | 16.852 | 16.673 | 11.705 | 12.288 | 9.327 | 12.530 | 16.186 | 6.403 | .000 |

Iteration history for the 3 dimensional solution (in squared distances)

Young's S-stress formula 1 is used.

| Iteration | S-stress | Improvement |
|---|---|---|

| | | |
|---|---|---|
| 1 | .16566 | |
| 2 | .11121 | .05445 |
| 3 | .10146 | .00975 |
| 4 | .09909 | .00237 |
| 5 | .09825 | .00084 |

Iterations stopped because
S-stress improvement is less than .001000

Stress and squared correlation (RSQ) in distances

RSQ values are the proportion of variance of the scaled data (disparities)
in the partition (row, matrix, or entire data) which
is accounted for by their corresponding distances.
Stress values are Kruskal's stress formula 1.

For matrix
Stress = .10131 RSQ = .95019

Configuration derived in 3 dimensions

Stimulus Coordinates

| Stimulus Number | Stimulus Name | Dimension 1 | 2 | 3 |
|---|---|---|---|---|
| 1 | notintun | 1.3782 | .6056 | .1135 |
| 2 | lackcomp | -2.6835 | -1.3917 | .7885 |
| 3 | noturnto | -.3386 | -.4544 | -.1442 |
| 4 | notalone | -1.0653 | -1.3651 | -.3396 |
| 5 | notpartg | .6723 | -.1127 | .4757 |
| 6 | notincom | .8696 | .3005 | .1574 |
| 7 | notclose | .3175 | -.6026 | -.6396 |
| 8 | notshare | -2.0473 | 1.0761 | 1.2653 |
| 9 | notoutgo | 1.1965 | .4368 | 1.0605 |
| 10 | noclosep | 1.9243 | -.2186 | -.1166 |
| 11 | leftout | -.6521 | -.1974 | -.6609 |

| | | | | |
|---|---|---|---|---|
| 12 | superfic | -1.9203 | 1.4111 | -.4902 |
| 13 | noonekno | -1.8715 | 1.1855 | -.6352 |
| 14 | isolated | -.0313 | -.1032 | -.4778 |
| 15 | notfindc | 1.7602 | -.5944 | 1.2941 |
| 16 | ppledont | .9763 | .3041 | -.0281 |
| 17 | unhappyw | .2363 | -.6290 | -.6670 |
| 18 | aroundno | -1.8495 | .5046 | -.6876 |
| 19 | nopeople | 1.7188 | -.0010 | -.0330 |
| 20 | nopeop_1 | 1.4092 | -.1542 | -.2351 |

Optimally scaled data (disparities) for subject 1

| | 1 | 2 | 3 | 4 | 5 | 6 | 7 | 8 | 9 | 10 |
|---|---|---|---|---|---|---|---|---|---|---|
| 1 | .000 | | | | | | | | | |
| 2 | 4.511 | .000 | | | | | | | | |
| 3 | 1.970 | 2.640 | .000 | | | | | | | |
| 4 | 3.160 | 1.676 | 1.232 | .000 | | | | | | |
| 5 | 1.003 | 3.456 | 1.286 | 2.266 | .000 | | | | | |
| 6 | .685 | 3.975 | 1.286 | 2.640 | .685 | .000 | | | | |
| 7 | 1.970 | 3.416 | 1.088 | 1.970 | 1.286 | 1.232 | .000 | | | |
| 8 | 3.653 | 2.854 | 2.734 | 3.160 | 3.223 | 3.160 | 3.114 | .000 | | |
| 9 | 1.412 | 4.190 | 2.266 | 3.223 | 1.286 | 1.088 | 2.266 | 3.456 | .000 | |
| 10 | 1.003 | 4.840 | 2.169 | 3.160 | 1.088 | 1.003 | 1.676 | 4.400 | 1.286 | .000 |
| 11 | 2.266 | 2.640 | 1.286 | 1.088 | 1.777 | 1.798 | 1.676 | 2.640 | 2.640 | 2.640 |
| 12 | 3.239 | 2.734 | 2.493 | 2.854 | 2.734 | 2.994 | 3.239 | 2.734 | 3.653 | 4.159 |
| 13 | 3.456 | 3.160 | 2.266 | 2.640 | 3.114 | 2.854 | 2.650 | 1.676 | 3.406 | 4.190 |
| 14 | 1.676 | 3.160 | 1.088 | 1.088 | 1.232 | 1.088 | 1.088 | 2.994 | 1.777 | 1.676 |
| 15 | 1.676 | 4.511 | 2.650 | 3.354 | 1.777 | 1.412 | 2.493 | 4.159 | 1.676 | 1.676 |
| 16 | 1.088 | 4.190 | 1.676 | 2.493 | 1.003 | .685 | 1.412 | 3.223 | 1.286 | 1.003 |
| 17 | 1.676 | 3.406 | 1.676 | 1.286 | 1.676 | 1.412 | 1.286 | 3.160 | 2.266 | 1.777 |
| 18 | 3.406 | 2.734 | 1.676 | 2.169 | 3.114 | 2.854 | 2.493 | 1.676 | 3.456 | 3.885 |
| 19 | .685 | 4.689 | 1.970 | 3.160 | 1.088 | .685 | 1.676 | 4.159 | 1.286 | .436 |
| 20 | 1.003 | 4.511 | 1.412 | 2.734 | 1.088 | .685 | 1.412 | 3.975 | 1.286 | .436 |

| | 11 | 12 | 13 | 14 | 15 | 16 | 17 | 18 | 19 | 20 |
|---|---|---|---|---|---|---|---|---|---|---|
| 11 | .000 | | | | | | | | | |
| 12 | 2.266 | .000 | | | | | | | | |
| 13 | 1.676 | 1.286 | .000 | | | | | | | |
| 14 | .685 | 2.640 | 1.970 | .000 | | | | | | |
| 15 | 3.160 | 4.511 | 4.511 | 2.650 | .000 | | | | | |
| 16 | 1.676 | 3.160 | 3.114 | 1.088 | 1.777 | .000 | | | | |

| | | | | | | | | | | |
|---|---|---|---|---|---|---|---|---|---|---|
| 17 | 1.088 | 3.160 | 2.734 | 1.088 | 2.493 | 1.412 | .000 | | | |
| 18 | 1.286 | 1.676 | 1.088 | 1.286 | 4.190 | 2.854 | 2.266 | .000 | | |
| 19 | 2.266 | 3.930 | 3.829 | 1.676 | 1.088 | .685 | 1.676 | 3.653 | .000 | |
| 20 | 1.676 | 3.653 | 3.566 | 1.088 | 1.286 | .685 | 1.286 | 3.406 | .400 | .000 |

Iteration history for the 2 dimensional solution (in squared distances)

Young's S-stress formula 1 is used.

| Iteration | S-stress | Improvement |
|---|---|---|
| 1 | .21273 | |
| 2 | .14270 | .07003 |
| 3 | .13325 | .00945 |
| 4 | .13126 | .00199 |
| 5 | .13046 | .00079 |

Iterations stopped because
S-stress improvement is less than .001000

Stress and squared correlation (RSQ) in distances

RSQ values are the proportion of variance of the scaled data (disparities)
in the partition (row, matrix, or entire data) which
is accounted for by their corresponding distances.
Stress values are Kruskal's stress formula 1.

For matrix
Stress = .14536 RSQ = .91648

Configuration derived in 2 dimensions

Stimulus Coordinates

| | | Dimension | |
|---|---|---|---|
| Stimulus Number | Stimulus Name | 1 | 2 |
| 1 | notintun | 1.1570 | -.5006 |

| | | | |
|---|---|---|---|
| 2 | lackcomp | -2.3038 | 1.2632 |
| 3 | noturnto | -.2874 | .2810 |
| 4 | notalone | -.8379 | 1.1039 |
| 5 | notpartg | .5455 | .0065 |
| 6 | notincom | .6957 | -.2667 |
| 7 | notclose | .1961 | .4868 |
| 8 | notshare | -1.7823 | -1.2578 |
| 9 | notoutgo | 1.2144 | -.5693 |
| 10 | noclosep | 1.6456 | .3263 |
| 11 | leftout | -.5340 | .1920 |
| 12 | superfic | -1.7327 | -1.0192 |
| 13 | noonekno | -1.6768 | -.8969 |
| 14 | isolated | -.0422 | .1205 |
| 15 | notfindc | 1.7472 | .6440 |
| 16 | ppledont | .8193 | -.3352 |
| 17 | unhappyw | .1411 | .4870 |
| 18 | aroundno | -1.6308 | -.3532 |
| 19 | nopeople | 1.4777 | .1567 |
| 20 | nopeop_1 | 1.1882 | .1311 |

Optimally scaled data (disparities) for subject 1

| | 1 | 2 | 3 | 4 | 5 | 6 | 7 | 8 | 9 | 10 |
|---|---|---|---|---|---|---|---|---|---|---|
| 1 | .000 | | | | | | | | | |
| 2 | 3.820 | .000 | | | | | | | | |
| 3 | 1.605 | 2.079 | .000 | | | | | | | |
| 4 | 2.531 | 1.253 | .805 | .000 | | | | | | |
| 5 | .805 | 2.945 | .820 | 1.770 | .000 | | | | | |
| 6 | .613 | 3.356 | .881 | 2.049 | .613 | .000 | | | | |
| 7 | 1.605 | 2.752 | .805 | 1.605 | .805 | .805 | .000 | | | |
| 8 | 3.077 | 2.440 | 2.079 | 2.550 | 2.682 | 2.493 | 2.440 | .000 | | |
| 9 | 1.091 | 3.690 | 1.770 | 2.682 | .881 | .805 | 1.770 | 3.059 | .000 | |
| 10 | .805 | 4.059 | 1.770 | 2.550 | .805 | .805 | 1.253 | 3.776 | .881 | .000 |
| 11 | 1.770 | 2.079 | .881 | .805 | 1.350 | 1.350 | 1.253 | 2.049 | 2.079 | 2.079 |
| 12 | 2.682 | 2.079 | 1.944 | 2.316 | 2.079 | 2.440 | 2.682 | 2.079 | 3.077 | 3.636 |
| 13 | 2.945 | 2.493 | 1.770 | 2.049 | 2.440 | 2.440 | 2.079 | 1.253 | 2.752 | 3.690 |
| 14 | 1.253 | 2.493 | .805 | .805 | .805 | .805 | .805 | 2.440 | 1.350 | 1.253 |
| 15 | 1.253 | 3.879 | 2.079 | 2.682 | 1.350 | 1.091 | 1.864 | 3.690 | 1.253 | 1.253 |
| 16 | .805 | 3.690 | 1.253 | 1.864 | .805 | .613 | 1.091 | 2.682 | 1.003 | .805 |
| 17 | 1.253 | 2.752 | 1.253 | 1.003 | 1.253 | 1.091 | 1.003 | 2.493 | 1.770 | 1.350 |
| 18 | 2.752 | 2.079 | 1.253 | 1.770 | 2.440 | 2.316 | 1.864 | 1.253 | 2.945 | 3.346 |
| 19 | .613 | 3.940 | 1.605 | 2.531 | .805 | .613 | 1.253 | 3.690 | .805 | .390 |

| | | | | | | | | | | |
|---|---|---|---|---|---|---|---|---|---|---|
| 20 | .805 | 3.879 | 1.091 | 2.079 | .805 | .613 | 1.091 | 3.356 | .805 | .390 |

| | 11 | 12 | 13 | 14 | 15 | 16 | 17 | 18 | 19 | 20 |
|---|---|---|---|---|---|---|---|---|---|---|
| 11 | .000 | | | | | | | | | |
| 12 | 1.770 | .000 | | | | | | | | |
| 13 | 1.253 | 1.003 | .000 | | | | | | | |
| 14 | .613 | 2.079 | 1.605 | .000 | | | | | | |
| 15 | 2.493 | 3.879 | 3.820 | 2.079 | .000 | | | | | |
| 16 | 1.253 | 2.550 | 2.493 | .805 | 1.349 | .000 | | | | |
| 17 | .805 | 2.550 | 2.079 | .805 | 1.864 | 1.091 | .000 | | | |
| 18 | 1.003 | 1.253 | .805 | 1.003 | 3.690 | 2.440 | 1.770 | .000 | | |
| 19 | 1.770 | 3.356 | 3.326 | 1.253 | .805 | .613 | 1.253 | 3.077 | .000 | |
| 20 | 1.253 | 3.077 | 3.059 | .805 | .820 | .613 | 1.003 | 2.752 | .291 | .000 |

| Abbreviated Name | Extended Name |
|---|---|
| aroundno | aroundnotwith |
| lackcomp | lackcompanionship |
| noclosep | noclosepeople |
| noonekno | nooneknowsme |
| nopeop_1 | nopeopletoturnto |
| nopeople | nopeopletotalkto |
| notclose | notcloseany |
| notfindc | notfindcomp |
| notincom | notincommon |
| notintun | notintune |
| notoutgo | notoutgoing |
| notpartg | notpartgroup |
| notshare | notshared |
| ppledont | ppledontunderstandme |
| superfic | superficial |
| unhappyw | unhappywithdrawn |
