# Supplementary material for: The Mental Representation of Social Connections: Generalizability Extended to Beijing Adults
Source: PLoS One. 2012 Sep 11;7(9):e44065. doi: 10.1371/journal.pone.0044065 (PMC3442957; doi:10.1371/journal.pone.0044065)
Supplement: Figure S3 — Multi-dimensional scaling analysis of Revised UCLA Loneliness Scale items in Beijing young adults (N = 267). (PDF) [file pone.0044065.s003.pdf]

Notes

Output Created		13-Feb-2012 16:06:17
Comments		
Input	Data	C:\Users\Louise Hawkley\Documents\lhawkley\MyPubs\Beijing Collaboration_UCLA Factor Analyses\UCLA_Beijing Younger Adults.sav
	Active Dataset	DataSet1
	Filter	<none>
	Weight	<none>
	Split File	<none>
	N of Rows in Working Data File	267
Syntax		ALSCAL<br>/MATRIX=IN('C:\Users\LOUISE~1\AppData\Local\Temp\spss376\spssalsc.tmp')<br>/LEVEL=ORDINAL<br>/CONDITION=MATRIX<br>/MODEL=EUCLID<br>/CRITERIA=CONVERGE(0.001) STRESSMIN(0.005) ITER(30) CUTOFF(0) DIMENS(2,3)<br>/PLOT=DEFAULT ALL<br>/PRINT=DATA HEADER.
Resources	Processor Time	00 00:00:04.009
	Elapsed Time	00 00:00:03.088

Derived Stimulus Configuration

Euclidean distance model

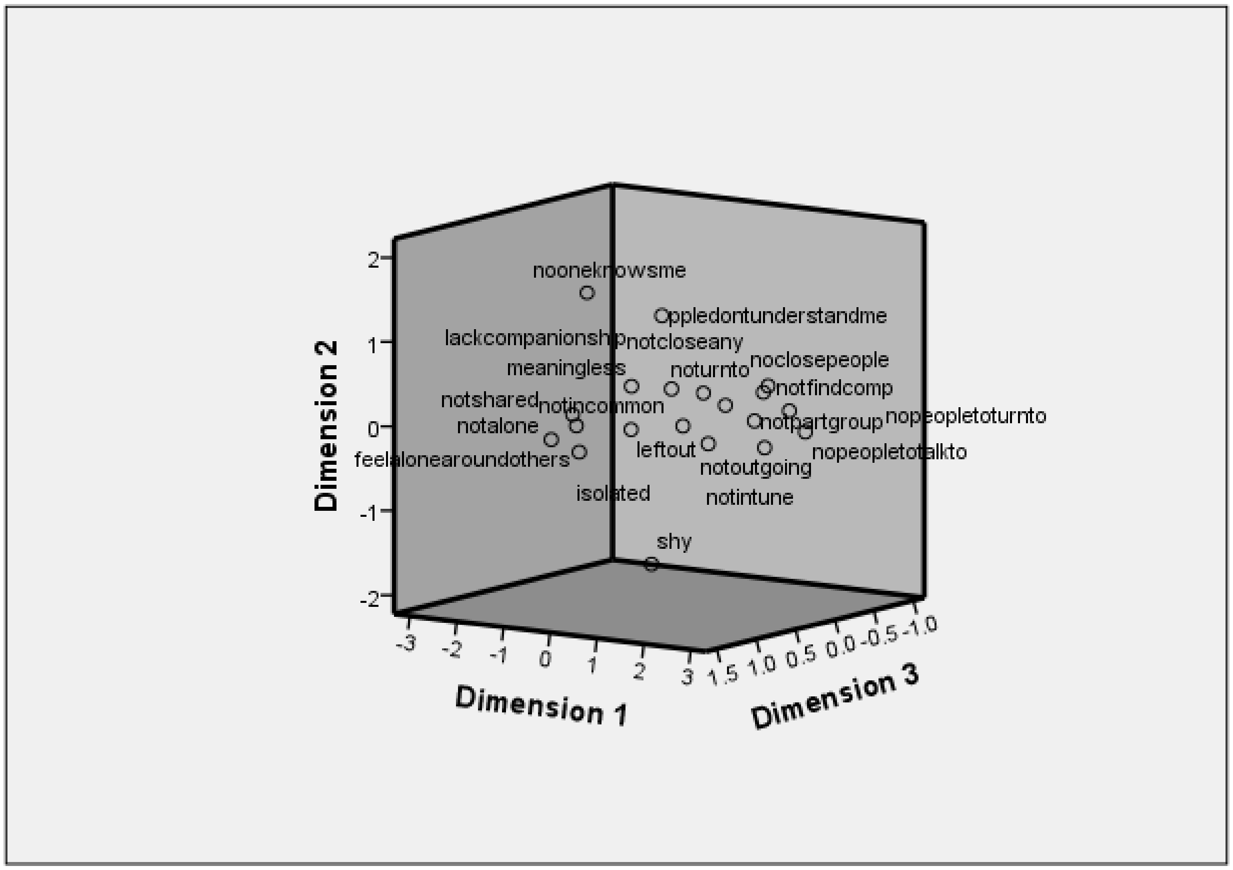

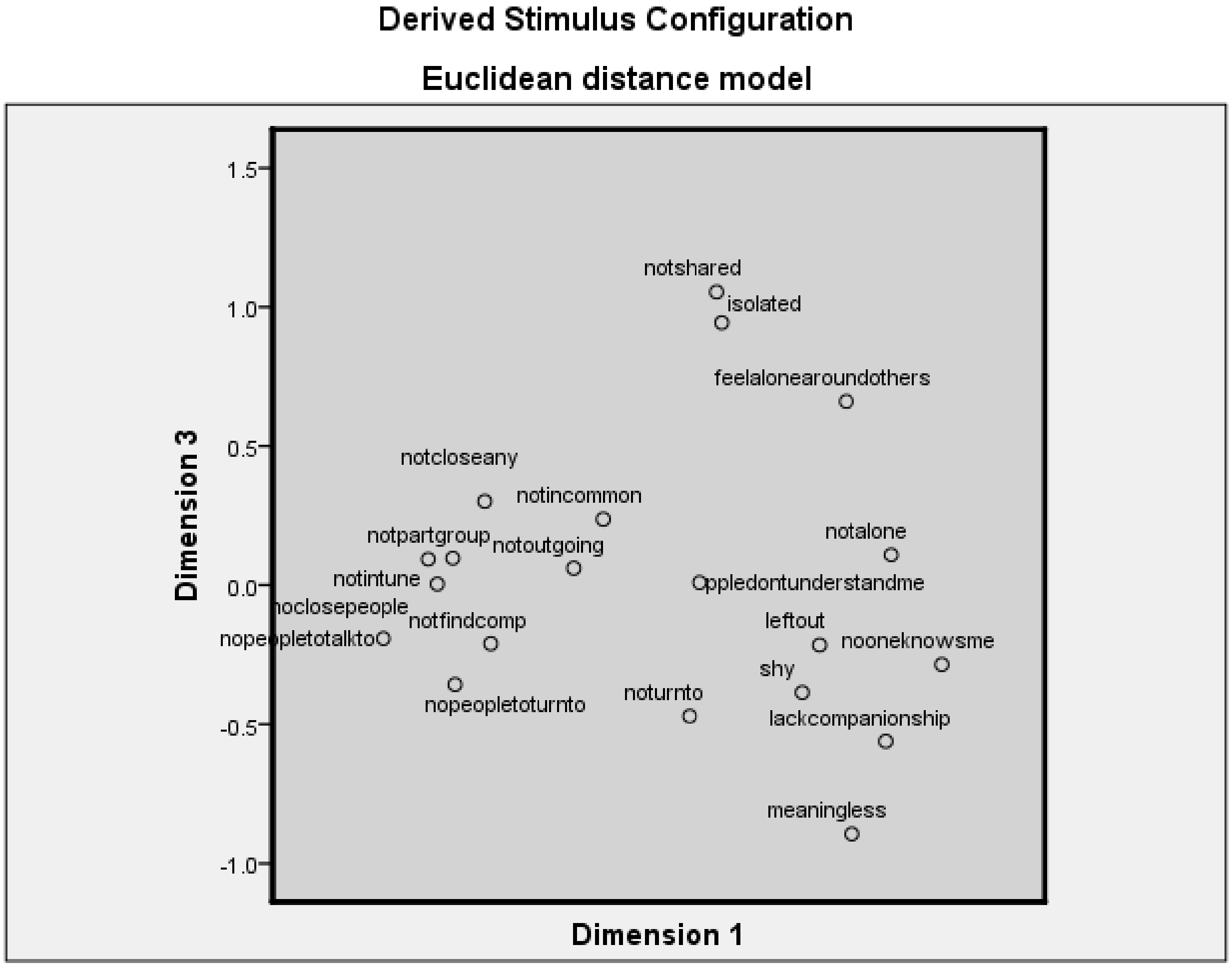
Derived Stimulus Configuration
Euclidean distance model
Dimension 3
Dimension 1
1.5
1.0
0.5
0.0
-0.5
-1.0
notshared
isolated
feelalonearoundothers
notcloseany
notincommon
notpartgroup
notoutgoing
notalone
notintune
ppledontunderstandme
noclosepeople
notfindcomp
leftout
nopeopletotalkto
nooneknowsme
shy
noturnto
nopeopletoturnto
lackcompanionship
meaningless

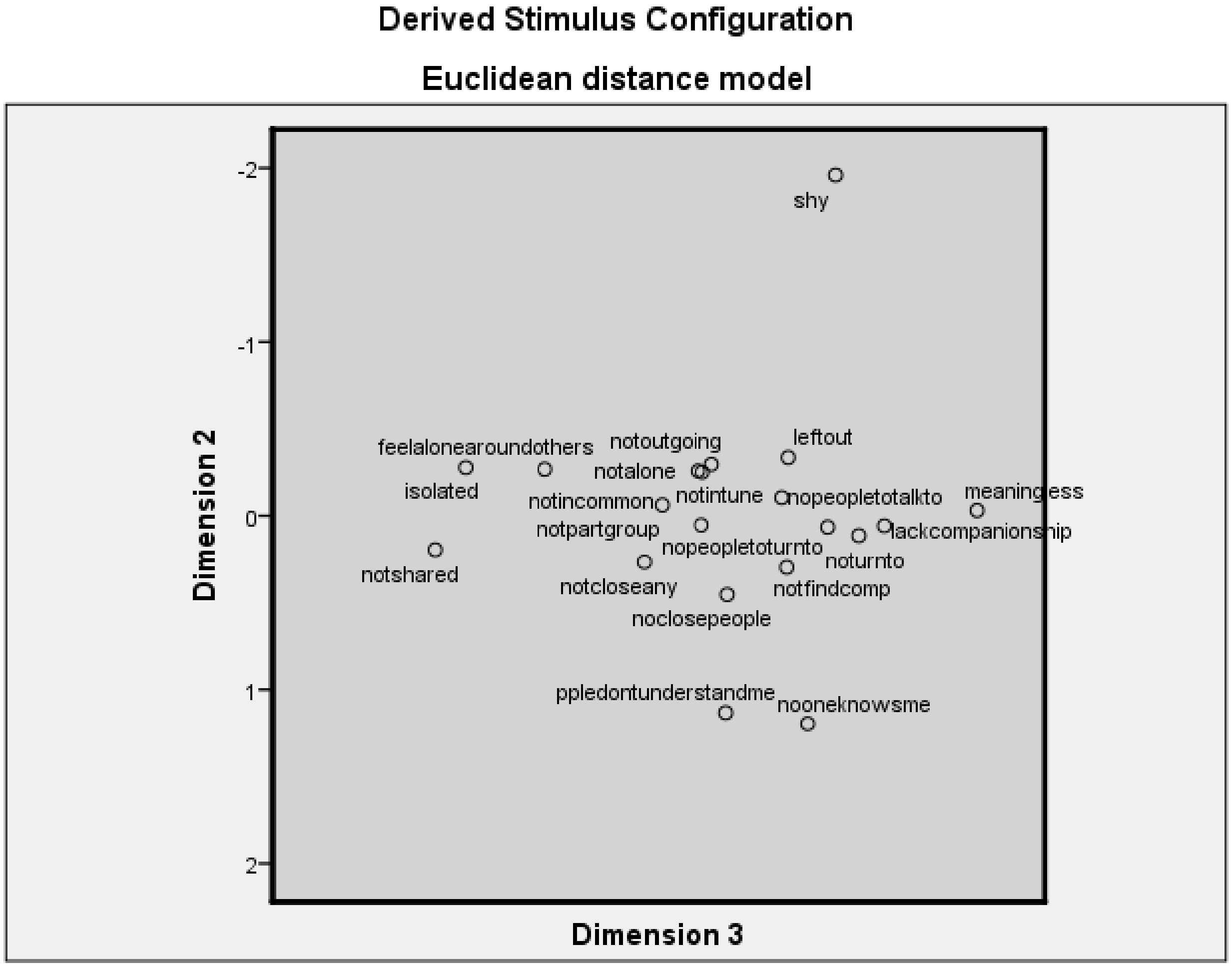

Derived Stimulus Configuration
Euclidean distance model
Dimension 2
Dimension 3
-2
-1
0
1
2
shy
feelalonearoundothers
notoutgoing
leftout
notalone
isolated
notincommon
notintune
nopeopletotalkto
meaningless
notpartgroup
lackcompanionship
nopeopletoturnto
noturnto
notshared
notcloseany
notfindcomp
noclosepeople
ppledontunderstandme
nooneknowsme

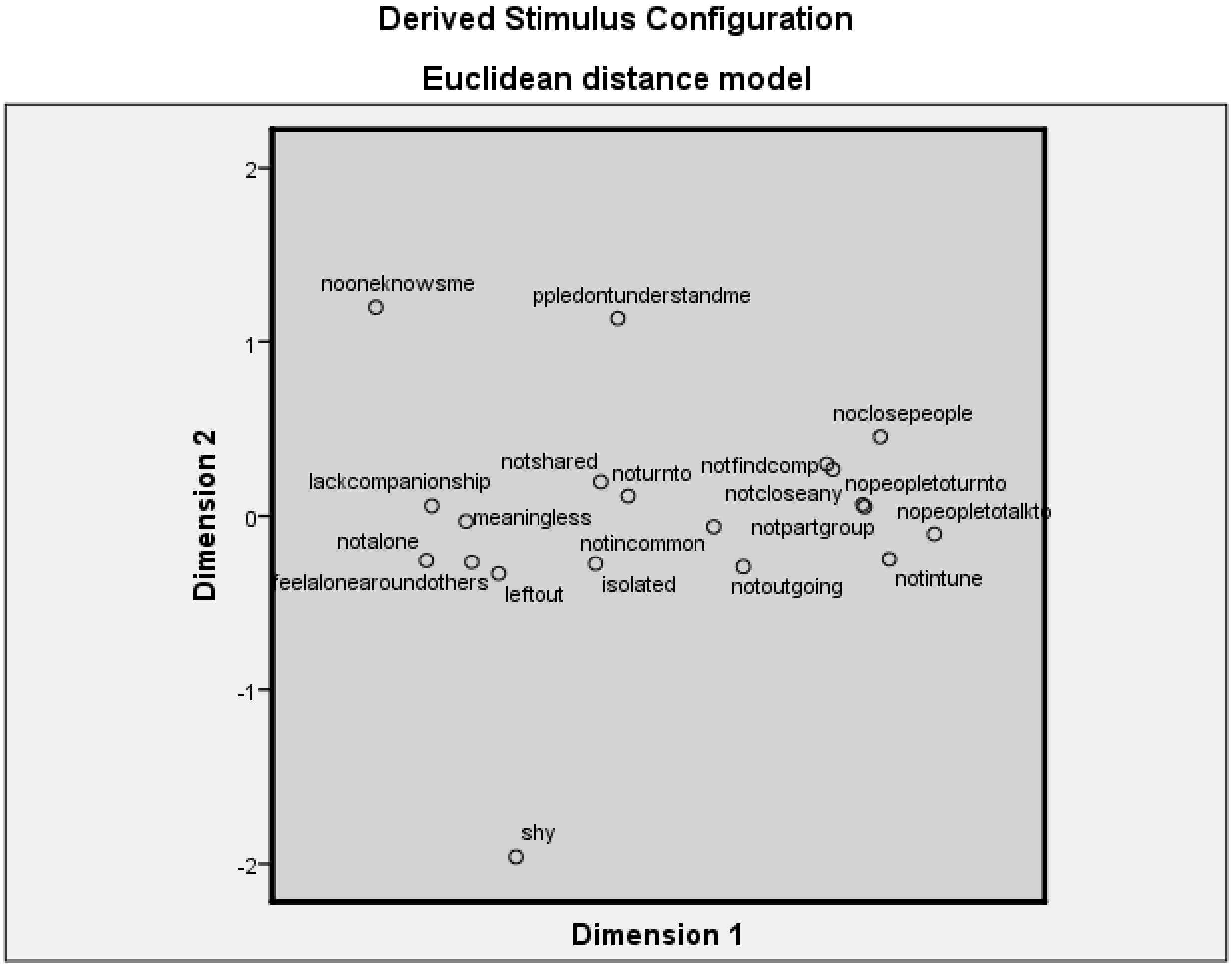

Derived Stimulus Configuration
Euclidean distance model
Dimension 2
Dimension 1
2
1
0
-1
-2
nooneknowsme
ppledontunderstandme
noclosepeople
notshared
noturnto
notfindcomp
lackcompanionship
notcloseany
nopeopletoturnto
meaningless
notpartgroup
nopeopletotalkto
notalone
notincommon
feelalonearoundothers
leftout
isolated
notoutgoing
notintune
shy

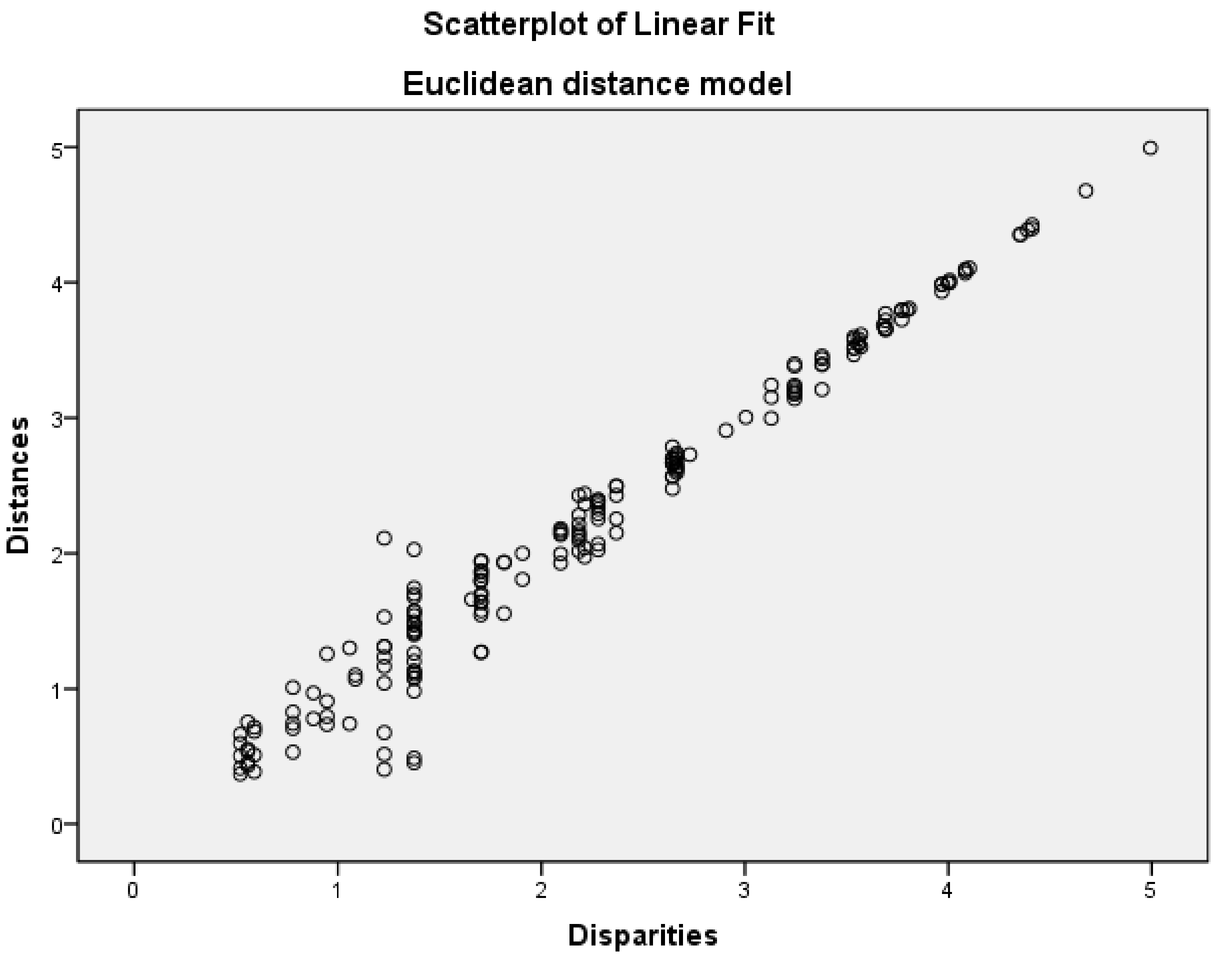
Scatterplot of Linear Fit
Euclidean distance model
Distances
Disparities
0
1
2
3
4
5

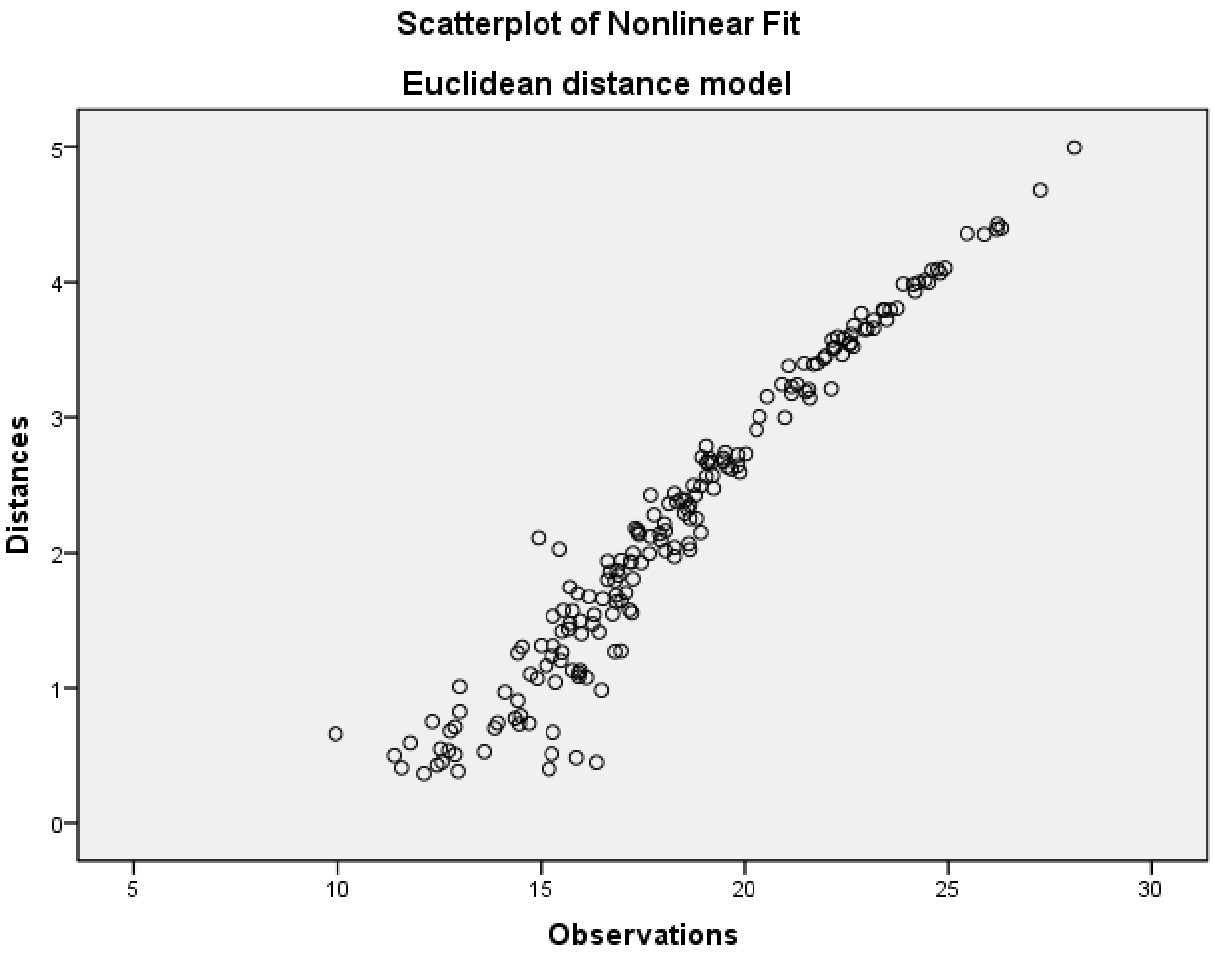

Scatterplot of Nonlinear Fit
Euclidean distance model
Distances
Observations
0
1
2
3
4
5
5
10
15
20
25
30

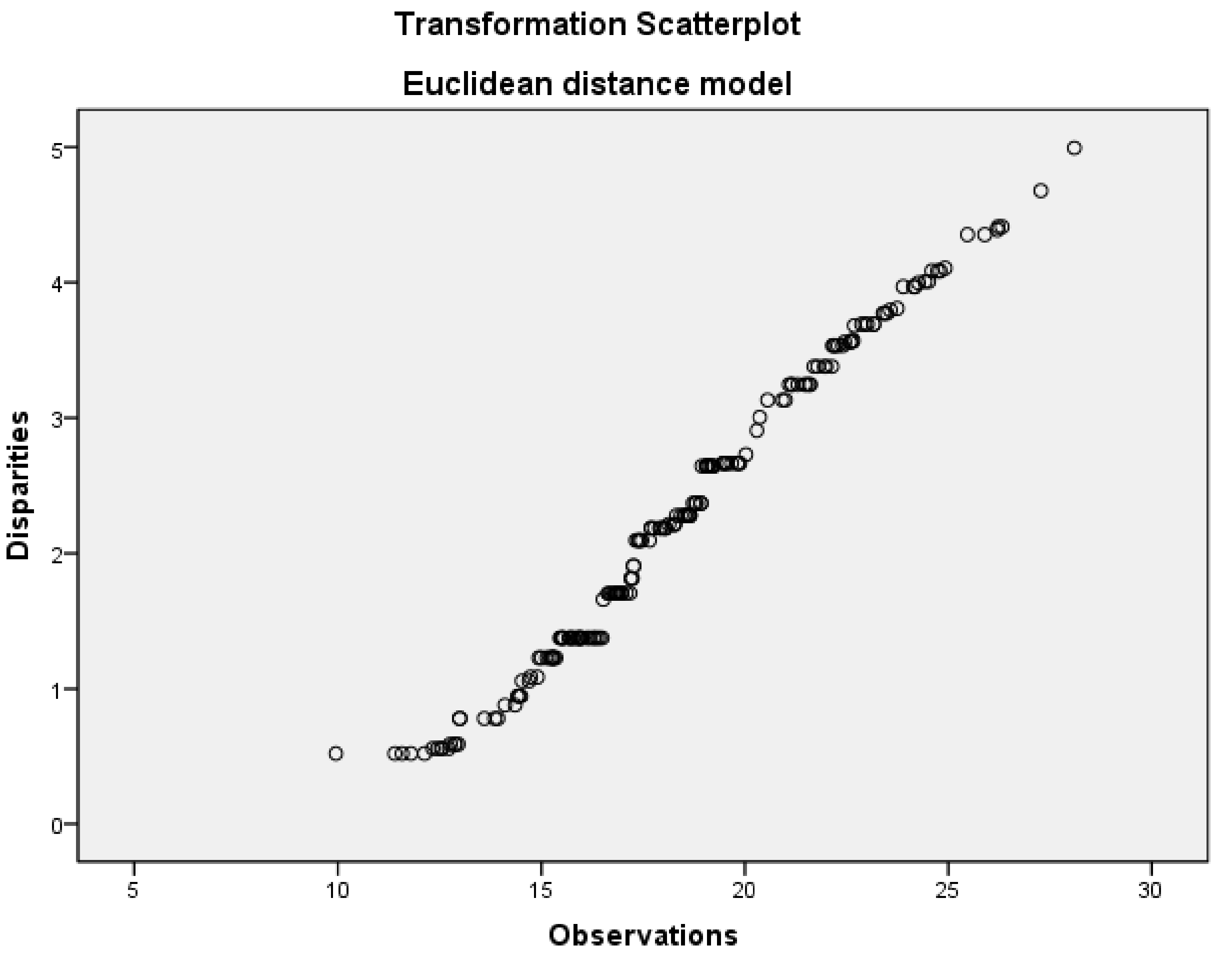
Transformation Scatterplot
Euclidean distance model
Disparities
Observations
0
1
2
3
4
5
5
10
15
20
25
30

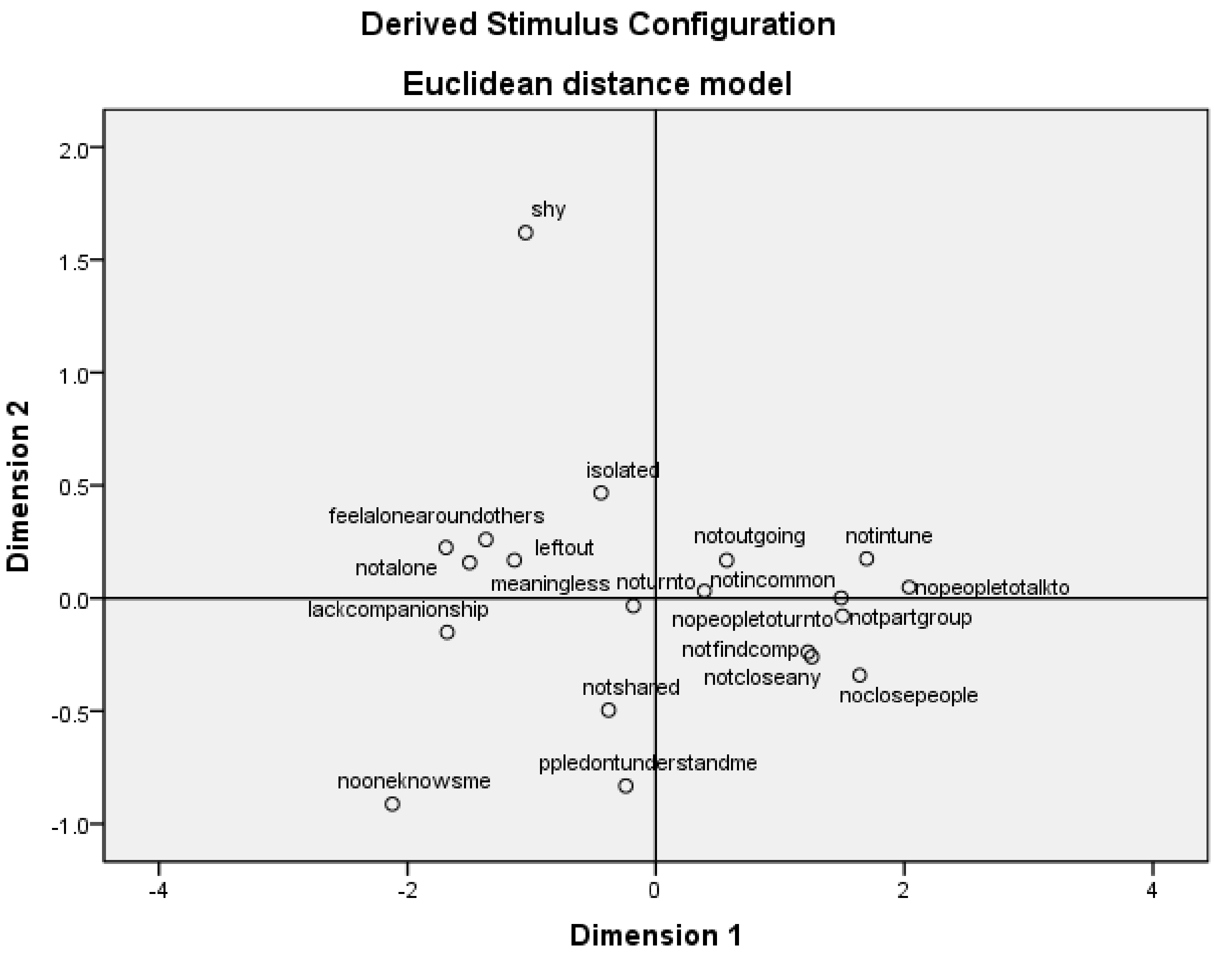

Derived Stimulus Configuration
Euclidean distance model
shy
isolated
feelalonearoundothers
leftout
notalone
meaningless
noturnto
notincommon
notoutgoing
notintune
nopeopletotalkto
lackcompanionship
nopeopletoturnto
notpartgroup
notfindcomp
notcloseany
noclosepeople
notshared
ppledontunderstandme
nooneknowsme
Dimension 1
Dimension 2
2.0
1.5
1.0
0.5
0.0
-0.5
-1.0
-4
-2
0
2
4

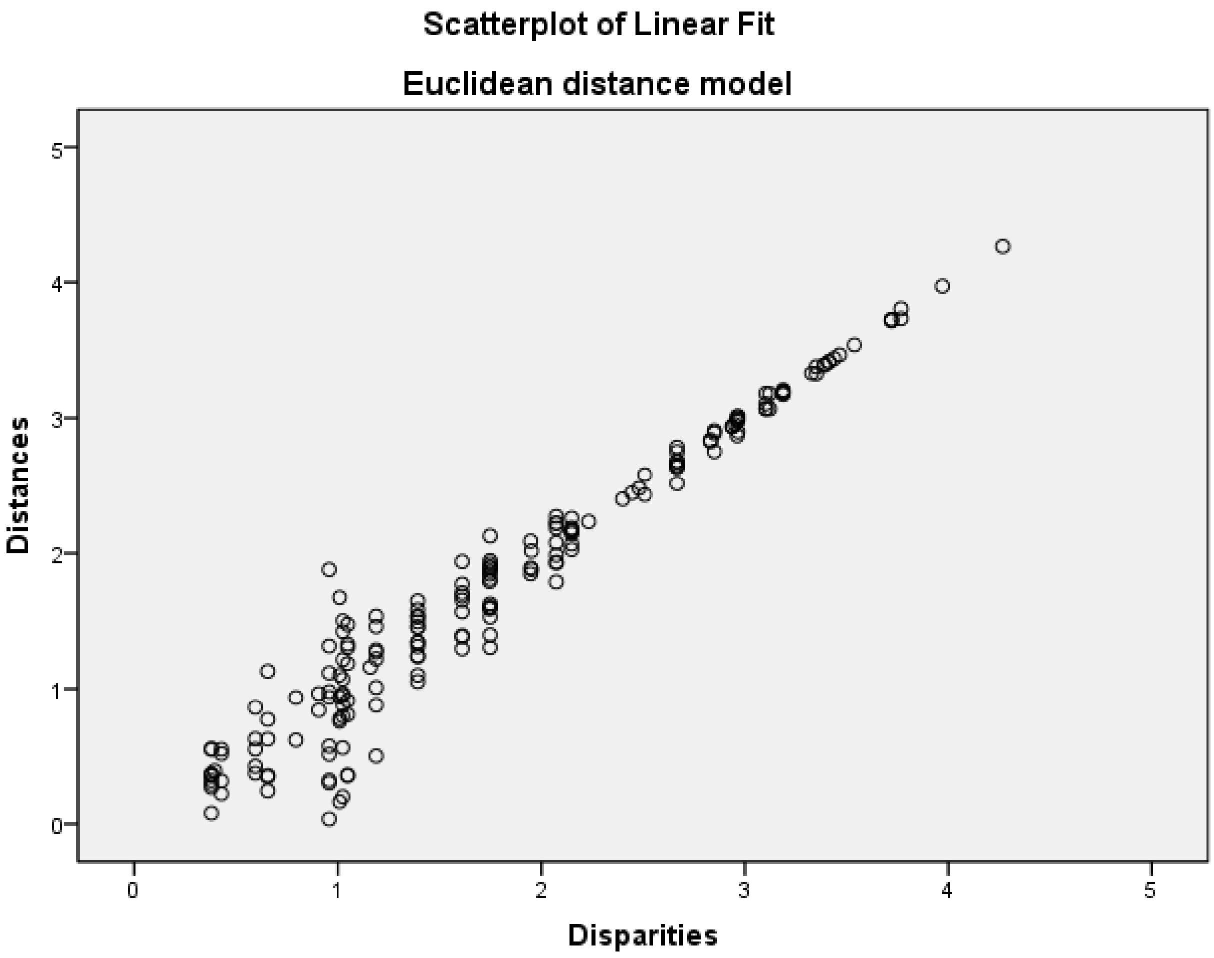

Scatterplot of Linear Fit
Euclidean distance model
Distances
Disparities
0
1
2
3
4
5

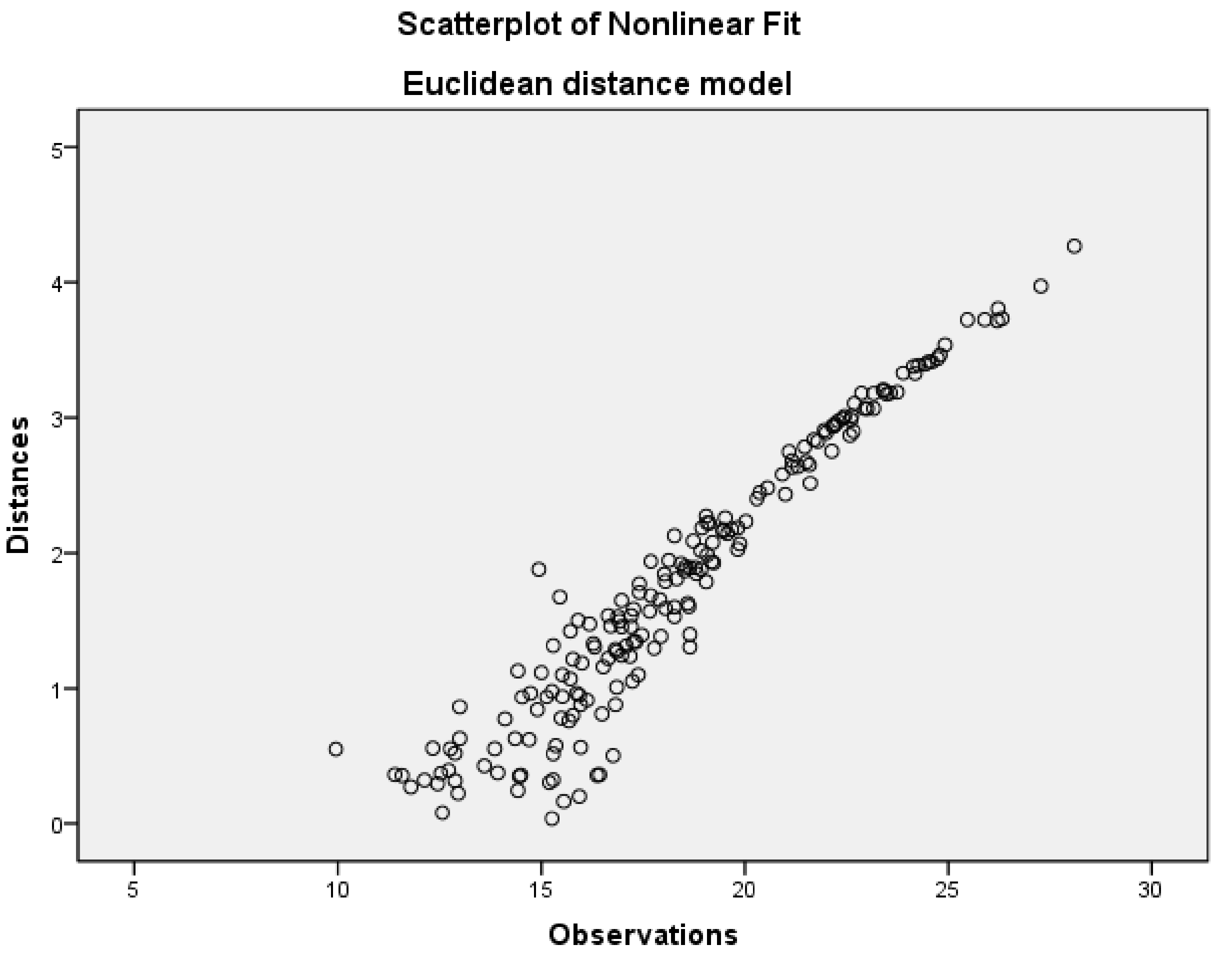

Scatterplot of Nonlinear Fit
Euclidean distance model
Distances
0
1
2
3
4
5
Observations
5
10
15
20
25
30

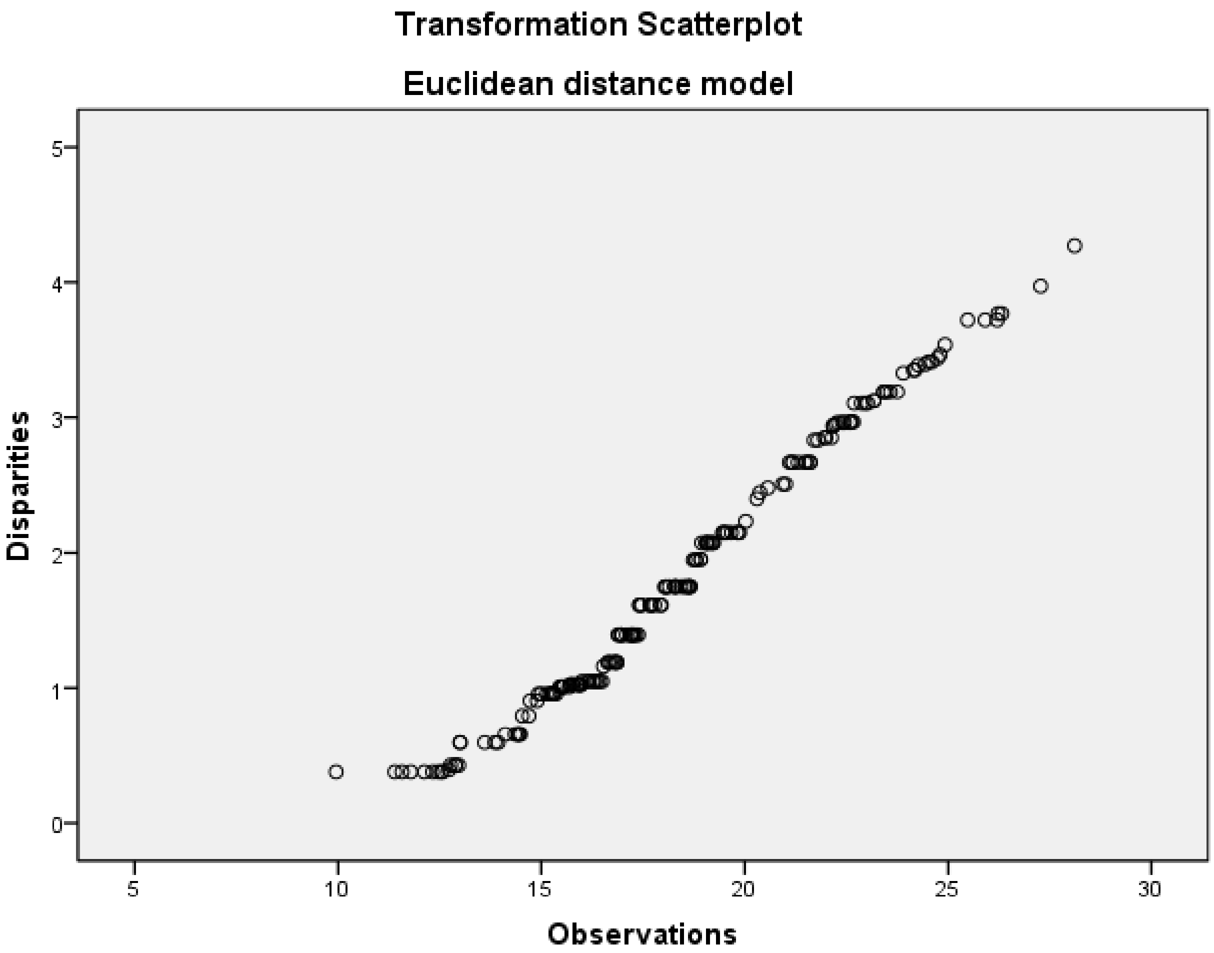

Transformation Scatterplot
Euclidean distance model
Disparities
Observations
0
1
2
3
4
5
5
10
15
20
25
30

Alscal Procedure Options

Data Options-

```
Number of Rows (Observations/Matrix).   20
Number of Columns (Variables) . . .   20
Number of Matrices   . . . . . . .    1
Measurement Level . . . . . . . .    Ordinal
Data Matrix Shape . . . . . . . .    Symmetric
Type  . . . . . . . . . . . . . .    Dissimilarity
Approach to Ties  . . . . . . . .    Leave Tied
Conditionality . . . . . . . . .    Matrix
Data Cutoff at . . . . . . . . .     .000000
```

Model Options-

```
Model . . . . . . . . . . . . . .    Euclid
Maximum Dimensionality  . . . . .    3
Minimum Dimensionality  . . . . .    2
Negative Weights  . . . . . . . .    Not Permitted
```

Output Options-

```
Job Option Header . . . . . . . .    Printed
Data Matrices  . . . . . . . . .    Printed
Configurations and Transformations  .    Plotted
Output Dataset . . . . . . . . .    Not Created
Initial Stimulus Coordinates  . . .    Computed
```

Algorithmic Options-

```
Maximum Iterations   . . . . . . .      30
Convergence Criterion   . . . . . .     .00100
Minimum S-stress  . . . . . . . .     .00500
Missing Data Estimated by  . . . .    Ulbounds
Tiestore . . . . . . . . . . . .  190
```

Raw (unscaled) Data for Subject 1

	1	2	3	4	5	6	7	8	9	10
1	.000									
2	24.434	.000								
3	18.655	15.906	.000							

4	24.269	13.928	16.882	.000						
5	12.124	23.580	17.692	23.409	.000					
6	15.297	19.209	15.362	18.735	15.000	.000				
7	14.697	22.159	17.205	22.605	15.199	16.125	.000			
8	18.947	17.321	16.763	16.912	19.235	14.526	17.916	.000		
9	14.422	19.053	15.492	19.519	14.731	12.961	16.492	16.523	.000	
10	12.884	24.187	18.815	24.145	12.450	16.310	12.728	19.824	16.000	.000
11	21.703	14.353	15.264	13.856	21.587	16.643	21.000	16.852	17.407	21.794
12	23.431	16.371	16.279	15.937	22.450	18.788	21.471	17.776	19.875	23.173
13	27.276	16.823	18.276	15.780	26.211	21.166	24.739	19.053	22.672	26.230
14	19.468	17.944	15.969	16.852	18.921	15.524	18.028	15.875	17.234	20.025
15	13.000	21.954	15.716	22.226	12.884	15.969	15.264	18.601	14.107	13.601
16	19.824	18.055	15.780	18.055	18.547	15.716	18.628	16.432	16.823	18.138
17	22.694	18.921	18.520	17.205	23.022	19.570	22.293	19.131	19.672	23.896
18	22.956	15.297	16.643	15.297	22.000	17.407	21.517	16.971	17.692	23.173
19	11.402	25.475	19.079	26.325	12.767	16.971	14.900	20.567	16.186	12.329
20	11.790	23.495	15.460	23.749	12.570	15.524	14.457	19.183	15.133	12.530

	11	12	13	14	15	16	17	18	19	20
11	.000									
12	14.491	.000								
13	16.703	17.176	.000							
14	15.684	17.378	19.468	.000						
15	20.298	22.136	24.515	18.330	.000					
16	17.263	18.276	14.933	17.088	17.263	.000				
17	16.971	18.276	21.095	18.655	22.181	20.928	.000			
18	14.422	15.556	17.464	15.937	21.307	17.664	18.655	.000		
19	22.869	24.920	28.107	21.610	13.000	20.372	24.799	24.597	.000	
20	21.166	22.405	25.904	19.079	11.576	18.439	22.627	22.583	9.950	.000

Iteration history for the 3 dimensional solution (in squared distances)

Young's S-stress formula 1 is used.

Iteration	S-stress	Improvement
1	.10691	
2	.07453	.03238
3	.07091	.00361
4	.06965	.00126
5	.06895	.00070

Iterations stopped because
S-stress improvement is less than .001000

Stress and squared correlation (RSQ) in distances

RSQ values are the proportion of variance of the scaled data (disparities)
in the partition (row, matrix, or entire data) which
is accounted for by their corresponding distances.

Stress values are Kruskal's stress formula 1.

For matrix
Stress = .08034 RSQ = .96862

Configuration derived in 3 dimensions

Stimulus Coordinates

Stimulus Number	Stimulus Name	Dimension 1	2	3
1	notintun	1.9893	-.2504	.0936
2	lackcomp	-1.9608	.0578	-.5619
3	noturnto	-.2652	.1143	-.4703
4	notalone	-2.0074	-.2574	.1089
5	notpartg	1.7781	.0531	.0971
6	notincom	.4794	-.0618	.2378
7	notclose	1.5031	.2672	.3020
8	notshare	-.5006	.1962	1.0540
9	notoutgo	.7329	-.2950	.0605
10	noclosep	1.9116	.4543	.0036
11	leftout	-1.3865	-.3342	-.2152
12	meaningl	-1.6661	-.0305	-.8940
13	noonekno	-2.4428	1.1971	-.2850
14	isolated	-.5449	-.2764	.9440
15	notfindc	1.4513	.2978	-.2101
16	ppledont	-.3528	1.1334	.0090
17	shy	-1.2374	-1.9599	-.3852
18	feelalon	-1.6178	-.2671	.6600
19	nopeople	2.3776	-.1039	-.1920
20	nopeop_1	1.7590	.0654	-.3567

Optimally scaled data (disparities) for subject 1

	1	2	3	4	5	6	7	8	9	10
1	.000									
2	4.007	.000								
3	2.280	1.374	.000							
4	3.997	.779	1.705	.000						
5	.521	3.796	2.186	3.772	.000					
6	1.228	2.646	1.228	2.369	1.228	.000				
7	1.058	3.535	1.816	3.561	1.228	1.374	.000			
8	2.646	2.096	1.703	1.705	2.646	1.058	2.186	.000		

9	.946	2.646	1.374	2.666	1.087	.589	1.374	1.658	.000	
10	.589	3.968	2.369	3.968	.558	1.374	.558	2.666	1.374	.000
11	3.380	.879	1.228	.779	3.245	1.703	3.131	1.703	2.096	3.380
12	3.772	1.374	1.374	1.374	3.561	2.369	3.245	2.186	2.666	3.693
13	4.678	1.703	2.213	1.374	4.390	3.245	4.084	2.646	3.571	4.412
14	2.666	2.186	1.374	1.703	2.369	1.374	2.186	1.374	1.816	2.730
15	.779	3.380	1.374	3.535	.589	1.374	1.228	2.280	.879	.779
16	2.666	2.186	1.374	2.186	2.280	1.374	2.280	1.374	1.703	2.213
17	3.683	2.369	2.280	1.816	3.693	2.666	3.535	2.646	2.666	3.968
18	3.693	1.228	1.703	1.228	3.380	2.096	3.245	1.705	2.186	3.693
19	.521	4.355	2.646	4.412	.589	1.705	1.087	3.131	1.374	.558
20	.521	3.772	1.374	3.809	.558	1.374	.946	2.646	1.228	.558

	11	12	13	14	15	16	17	18	19	20
11	.000									
12	.946	.000								
13	1.703	1.705	.000							
14	1.374	2.096	2.666	.000						
15	2.907	3.380	4.007	2.280	.000					
16	1.907	2.213	1.228	1.705	1.907	.000				
17	1.705	2.213	3.245	2.280	3.535	3.131	.000			
18	.946	1.374	2.096	1.374	3.245	2.096	2.280	.000		
19	3.693	4.105	4.994	3.245	.779	3.004	4.084	4.084	.000	
20	3.245	3.535	4.355	2.646	.521	2.280	3.571	3.561	.521	.000

Iteration history for the 2 dimensional solution (in squared distances)

Young's S-stress formula 1 is used.

Iteration	S-stress	Improvement
1	.12307	
2	.09408	.02899
3	.09096	.00312
4	.09003	.00093

Iterations stopped because
S-stress improvement is less than .001000

Stress and squared correlation (RSQ) in distances

RSQ values are the proportion of variance of the scaled data (disparities)
in the partition (row, matrix, or entire data) which
is accounted for by their corresponding distances.
Stress values are Kruskal's stress formula 1.

For matrix
Stress = .11620 RSQ = .94653

Configuration derived in 2 dimensions

Stimulus Coordinates

		Dimension	
Stimulus Number	Stimulus Name	1	2
1	notintun	1.6975	.1766
2	lackcomp	-1.6805	-.1500
3	noturnto	-.1835	-.0337
4	notalone	-1.6905	.2259
5	notpartg	1.5005	-.0787
6	notincom	.3895	.0330
7	notclose	1.2545	-.2585
8	notshare	-.3814	-.4957
9	notoutgo	.5683	.1696
10	noclosep	1.6397	-.3400
11	leftout	-1.1399	.1700
12	meaningl	-1.4992	.1593
13	noonekno	-2.1219	-.9103
14	isolated	-.4424	.4672
15	notfindc	1.2247	-.2376
16	ppledont	-.2434	-.8302
17	shy	-1.0508	1.6215
18	feelalon	-1.3684	.2609
19	nopeople	2.0378	.0495
20	nopeop_1	1.4895	.0012

Optimally scaled data (disparities) for subject 1

	1	2	3	4	5	6	7	8	9	10
1	.000									
2	3.394	.000								
3	1.748	1.025	.000							
4	3.388	.595	1.392	.000						
5	.378	3.189	1.612	3.189	.000					
6	.956	2.075	.956	1.946	.956	.000				
7	.794	2.937	1.392	2.966	.956	1.049	.000			
8	2.075	1.392	1.188	1.392	2.075	.794	1.612	.000		
9	.656	2.075	1.008	2.150	.905	.426	1.049	1.160	.000	
10	.426	3.352	1.946	3.352	.378	1.049	.394	2.150	1.049	.000
11	2.832	.656	.956	.595	2.668	1.188	2.508	1.188	1.612	2.832
12	3.189	1.049	1.049	1.025	2.962	1.946	2.668	1.612	2.150	3.123
13	3.971	1.188	1.748	1.025	3.722	2.668	3.439	2.075	2.966	3.769
14	2.150	1.612	1.025	1.188	1.950	1.008	1.746	1.025	1.392	2.233
15	.595	2.850	1.025	2.952	.426	1.025	.956	1.748	.656	.595

16	2.150	1.746	1.025	1.746	1.748	1.025	1.748	1.049	1.188	1.748
17	3.105	1.950	1.748	1.392	3.105	2.150	2.962	2.075	2.150	3.330
18	3.105	.956	1.188	.956	2.850	1.612	2.668	1.392	1.612	3.123
19	.378	3.722	2.075	3.769	.426	1.392	.905	2.480	1.049	.378
20	.378	3.189	1.008	3.189	.378	1.008	.656	2.075	.956	.378

	11	12	13	14	15	16	17	18	19	20
11	.000									
12	.656	.000								
13	1.188	1.392	.000							
14	1.008	1.392	2.150	.000						
15	2.400	2.850	3.413	1.748	.000					
16	1.392	1.748	.956	1.392	1.392	.000				
17	1.392	1.748	2.668	1.748	2.938	2.508	.000			
18	.656	1.008	1.612	1.025	2.668	1.612	1.748	.000		
19	3.105	3.539	4.269	2.668	.595	2.445	3.466	3.413	.000	
20	2.668	2.962	3.722	2.075	.378	1.748	2.966	2.962	.378	.000

Abbreviated Name	Extended Name
feelalon	feelalonearoundothers
lackcomp	lackcompanionship
meaningl	meaningless
noclosep	noclosepeople
noonekno	nooneknowsme
nopeop_1	nopeopletoturnto
nopeople	nopeopletotalkto
notclose	notcloseany
notfindc	notfindcomp
notincom	notincommon
notintun	notintune
notoutgo	notoutgoing
notpartg	notpartgroup
notshare	notshared
ppledont	ppledontunderstandme
