# Supplementary material for: The Mental Representation of Social Connections: Generalizability Extended to Beijing Adults
Source: PLoS One. 2012 Sep 11;7(9):e44065. doi: 10.1371/journal.pone.0044065 (PMC3442957; doi:10.1371/journal.pone.0044065)
Supplement: Figure S4 — Multi-dimensional scaling analysis of Revised UCLA Loneliness Scale items in Beijing older adults (N = 246). (PDF) [file pone.0044065.s004.pdf]

Notes

Output Created		13-Feb-2012 16:17:13
Comments		
Input	Data	C:\Users\Louise Hawkley\Documents\lhawkley\MyPubs\Beijing Collaboration_UCLA Factor Analyses\UCLA_Beijing Older Adults.sav
	Active Dataset	DataSet2
	Filter	<none>
	Weight	<none>
	Split File	<none>
	N of Rows in Working Data File	246
Syntax		ALSCAL /MATRIX=IN('C:\Users\LOUISE~1\AppData\Local\Temp\spss376\spssalsc.tmp') /LEVEL=ORDINAL /CONDITION=MATRIX /MODEL=EUCLID /CRITERIA=CONVERGE(0.001) STRESSMIN(0.005) ITER(30) CUTOFF(0) DIMENS(2,3) /PLOT=DEFAULT ALL /PRINT=HEADER.
Resources	Processor Time	00 00:00:02.090
	Elapsed Time	00 00:00:02.043

Derived Stimulus Configuration

Euclidean distance model

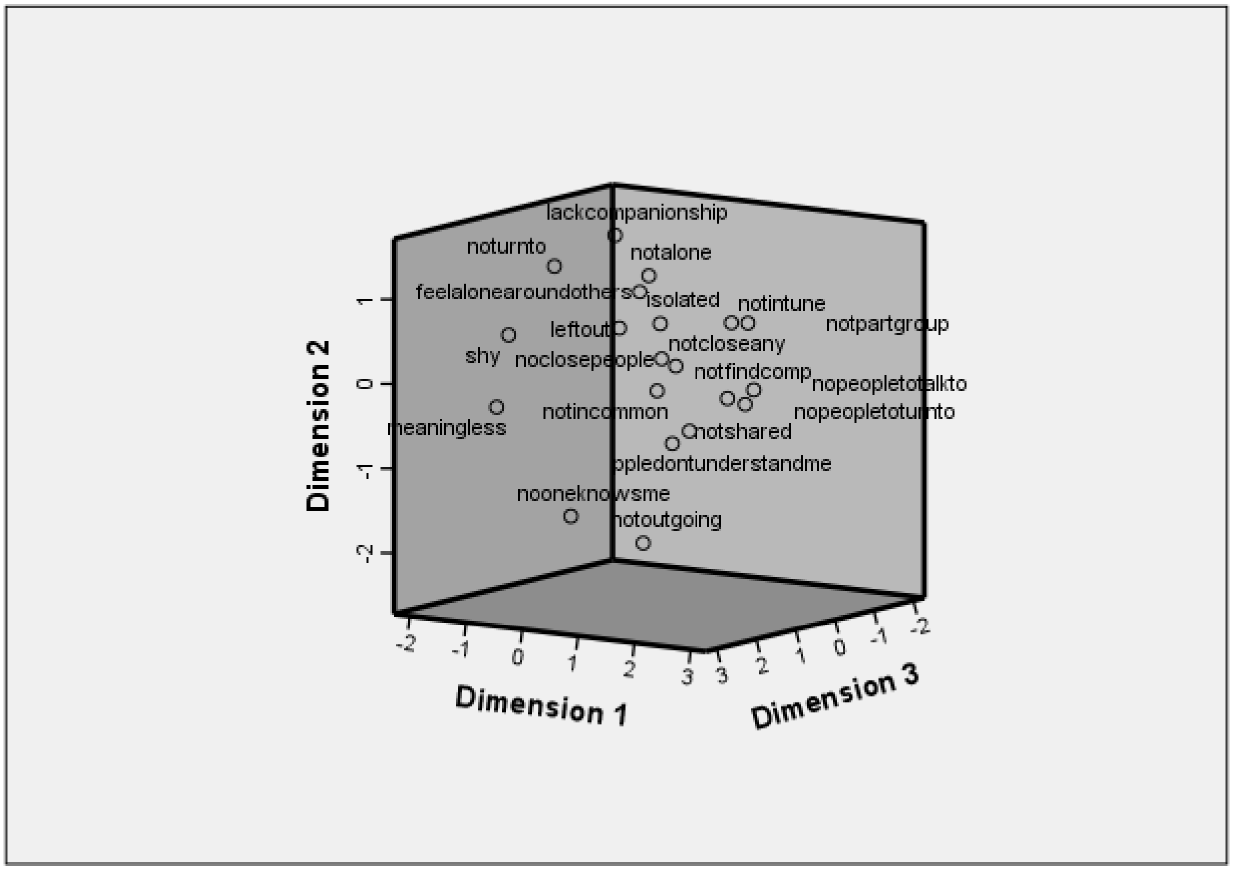

Derived Stimulus Configuration

Euclidean distance model

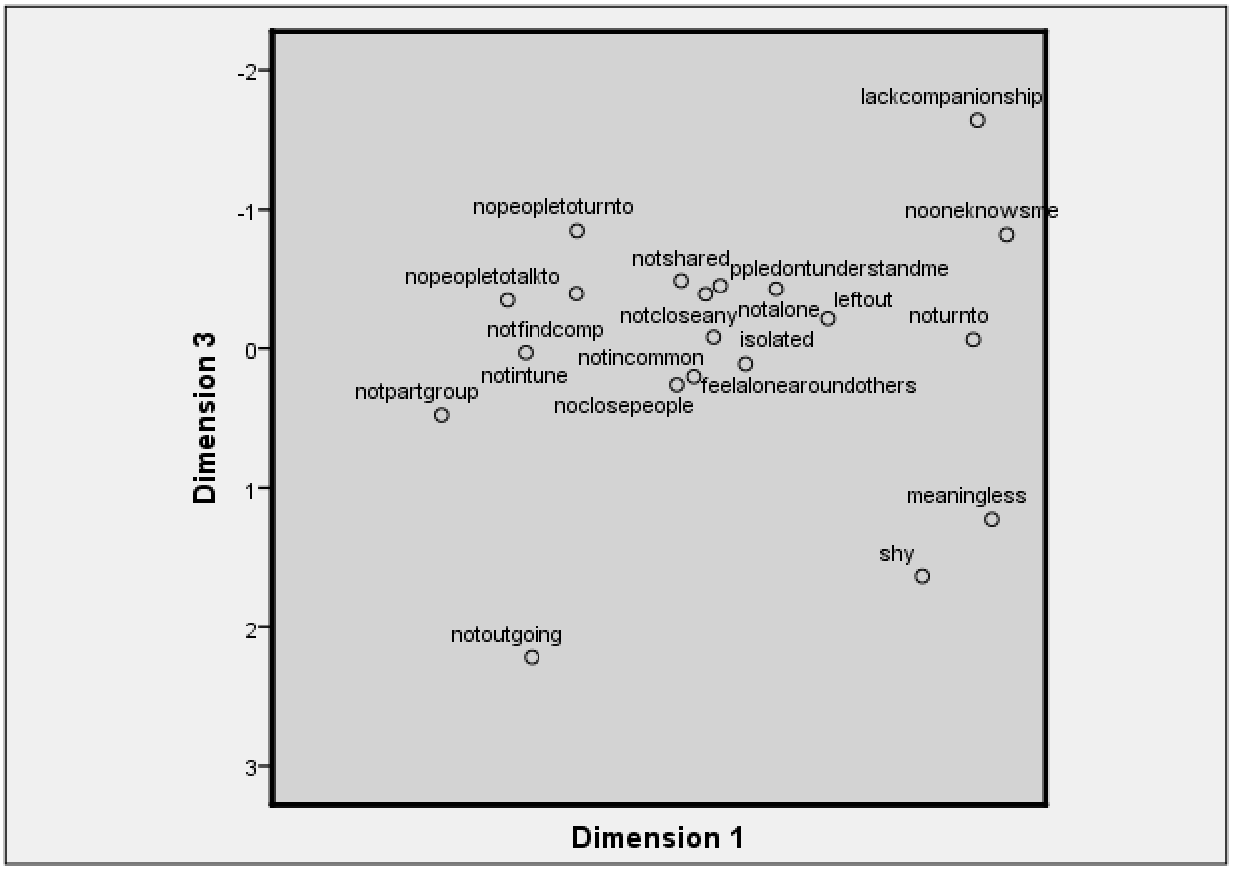

Derived Stimulus Configuration

Euclidean distance model

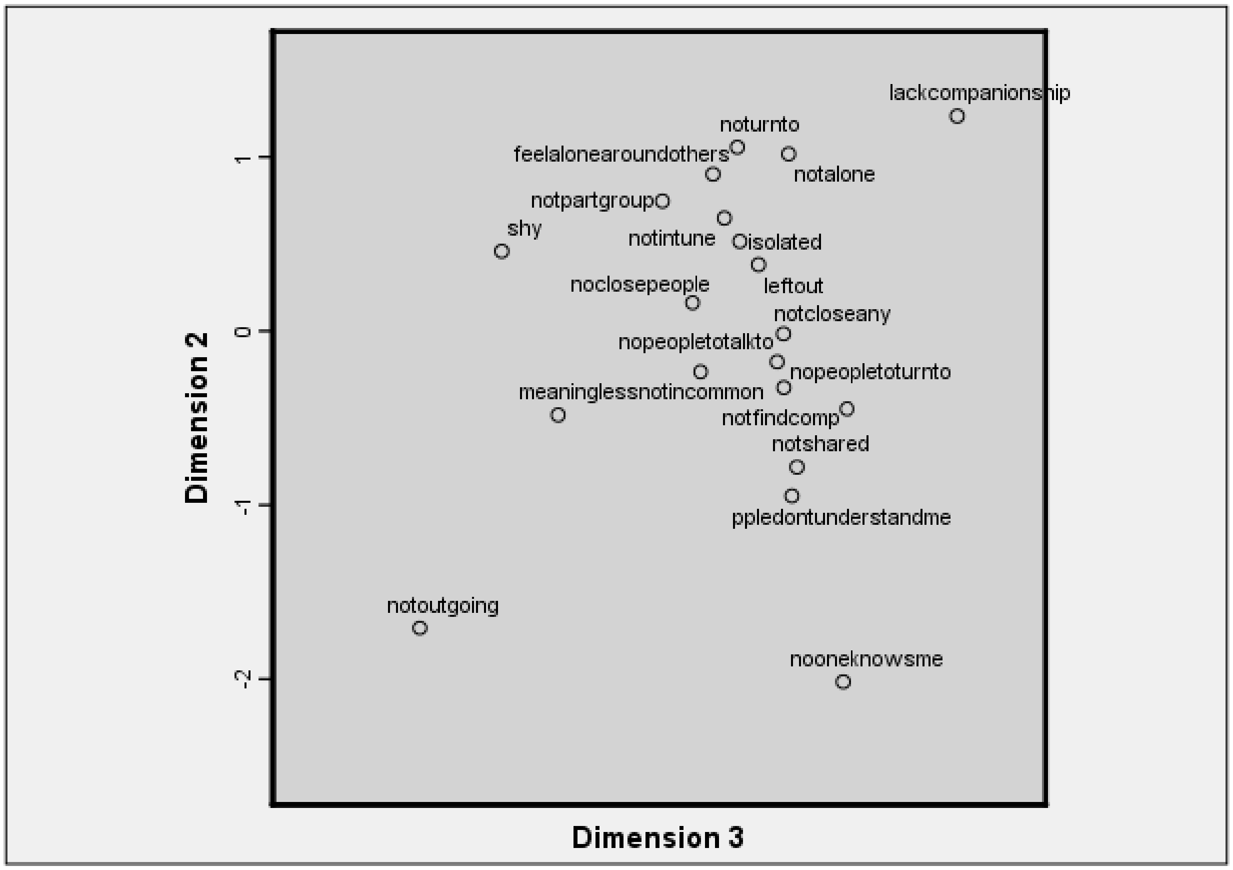

Derived Stimulus Configuration

Euclidean distance model

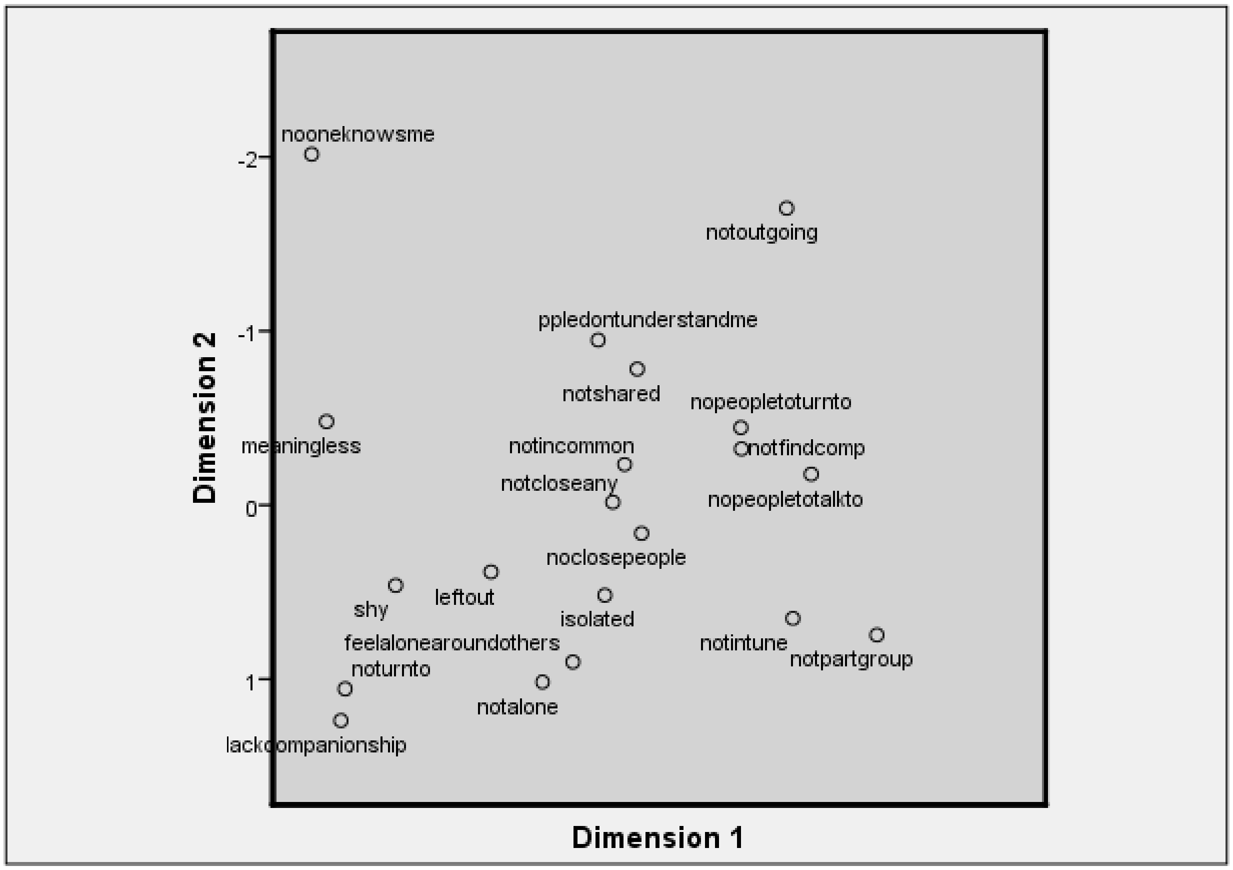

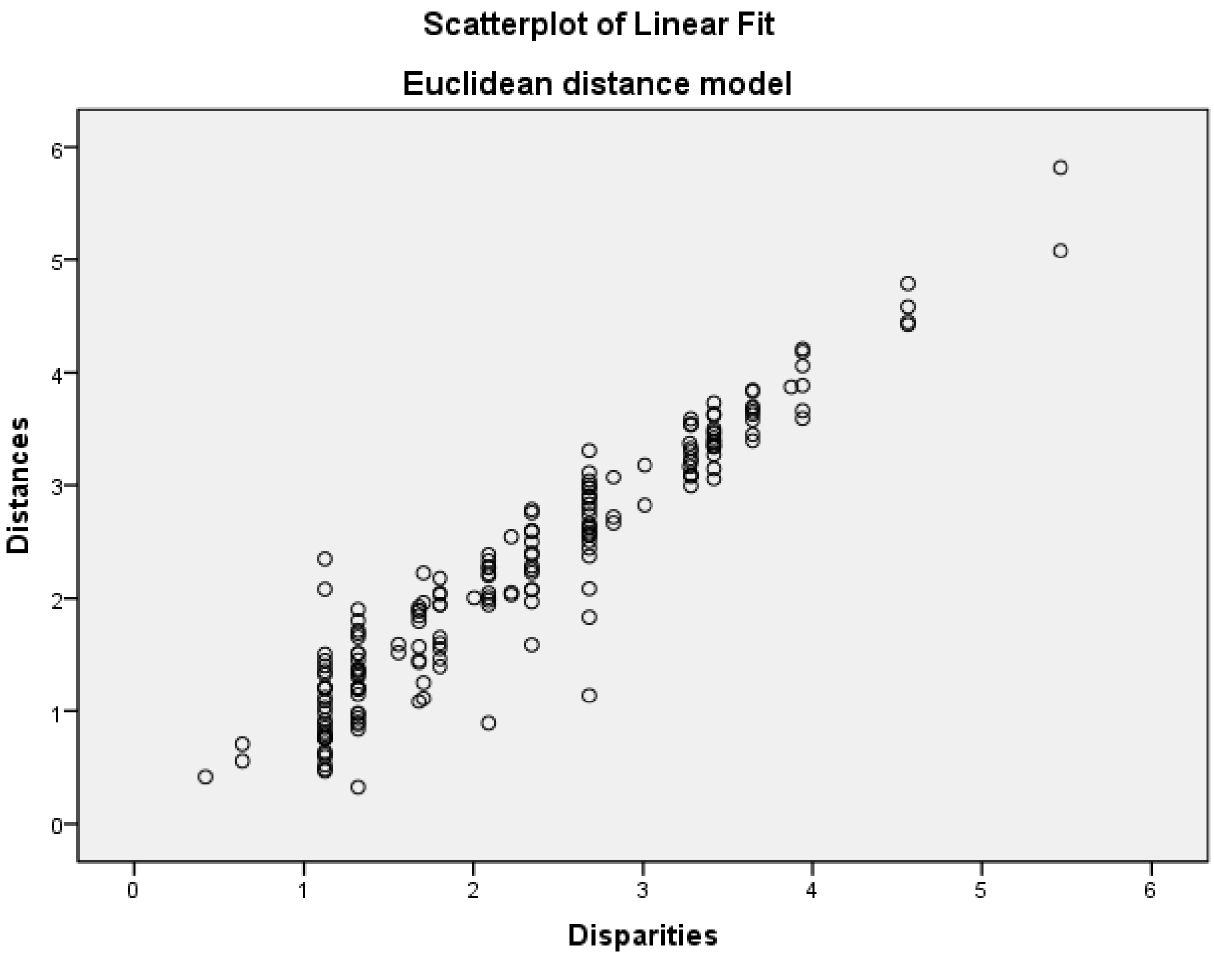

Scatterplot of Linear Fit
Euclidean distance model
Distances
Disparities
0
1
2
3
4
5
6

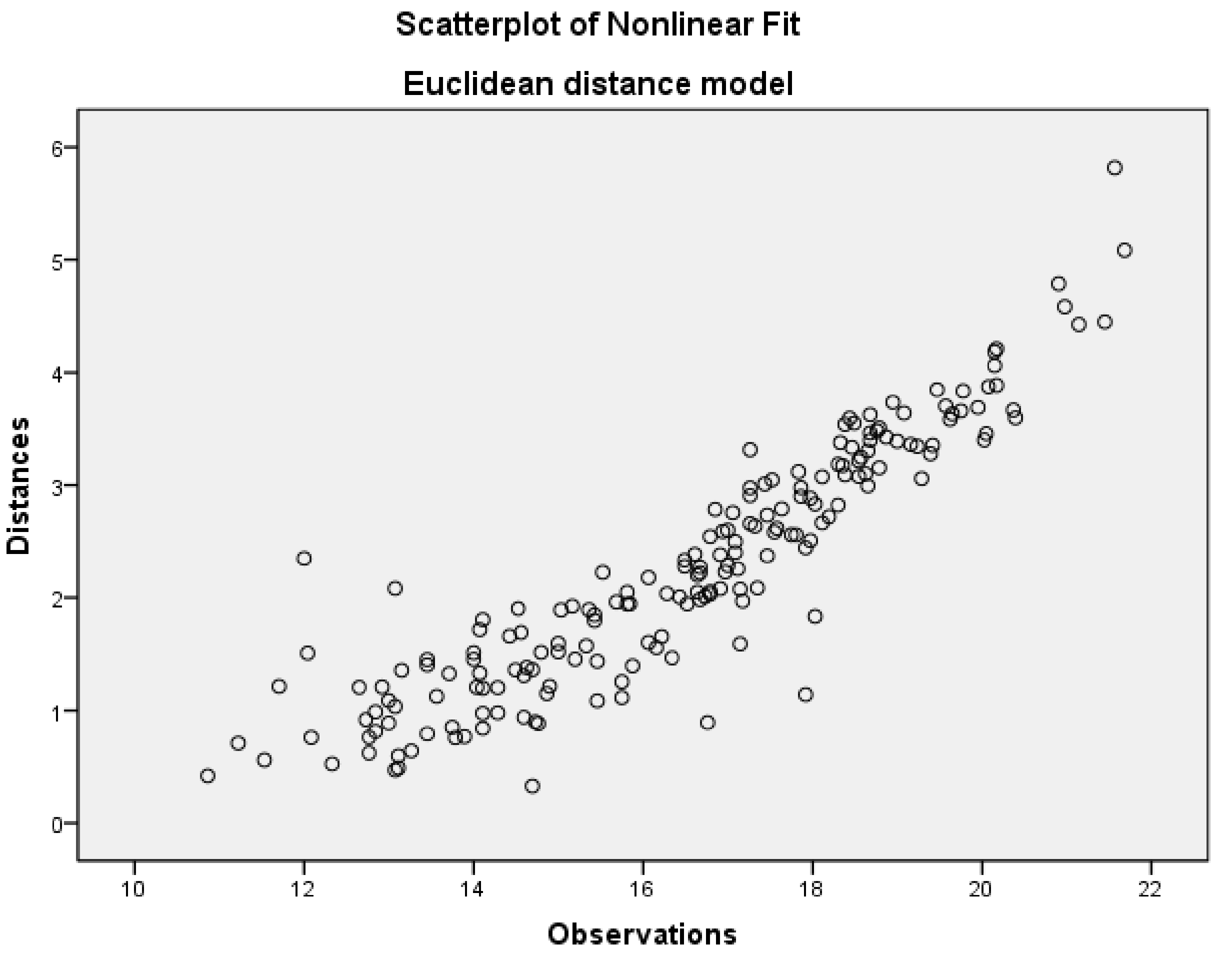
Scatterplot of Nonlinear Fit
Euclidean distance model
Distances
0
1
2
3
4
5
6
10
12
14
16
18
20
22
Observations

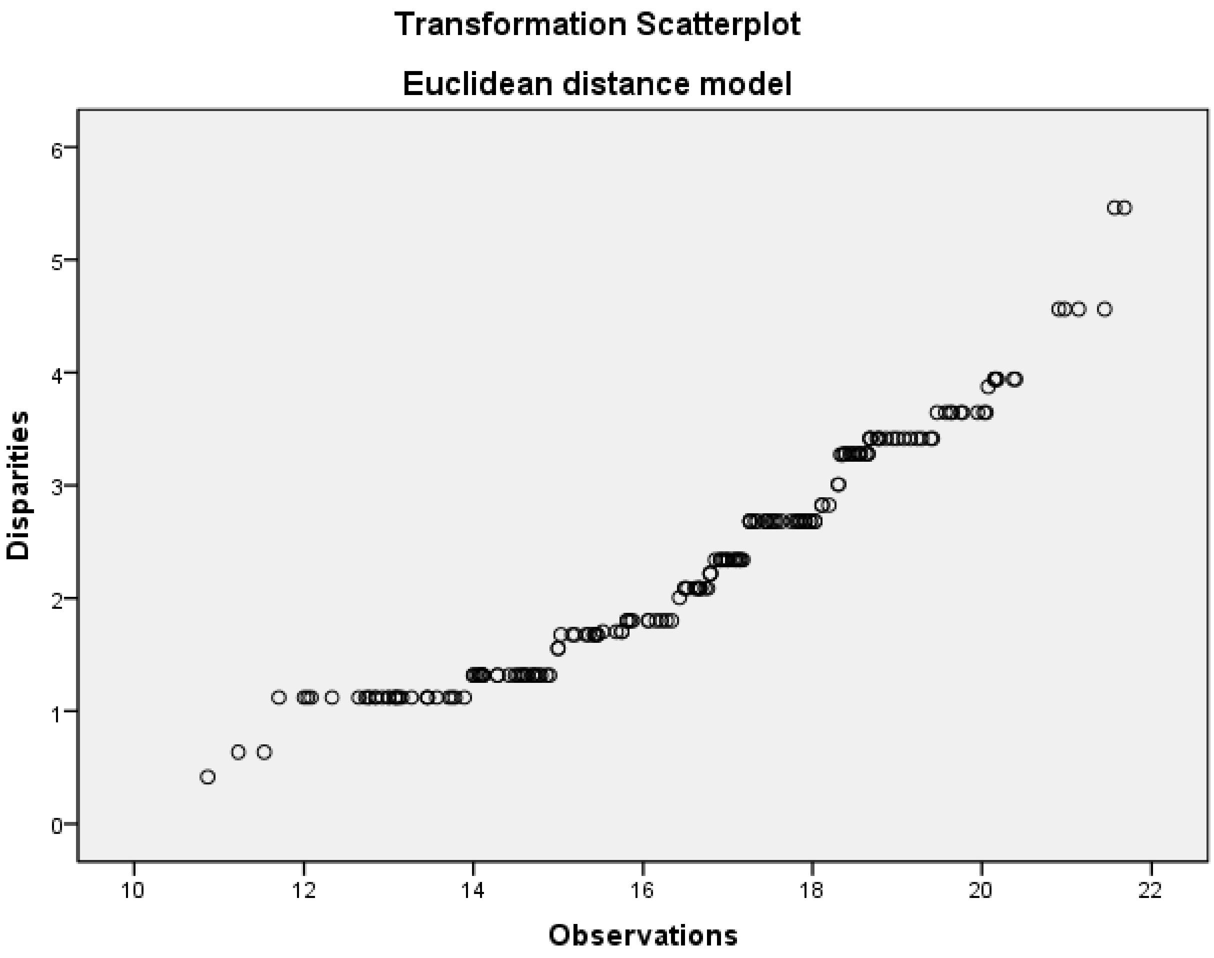

Transformation Scatterplot
Euclidean distance model
Disparities
Observations
0
1
2
3
4
5
6
10
12
14
16
18
20
22

Derived Stimulus Configuration

Euclidean distance model

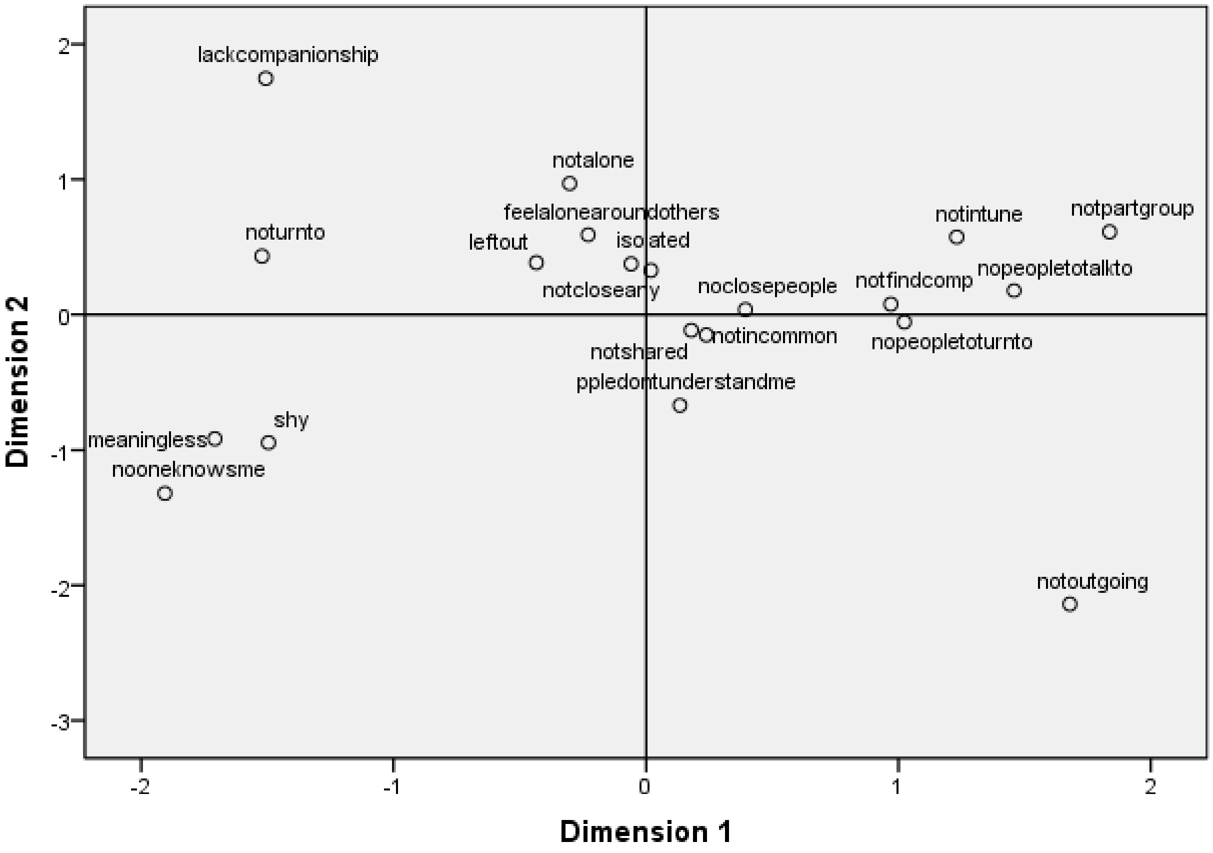

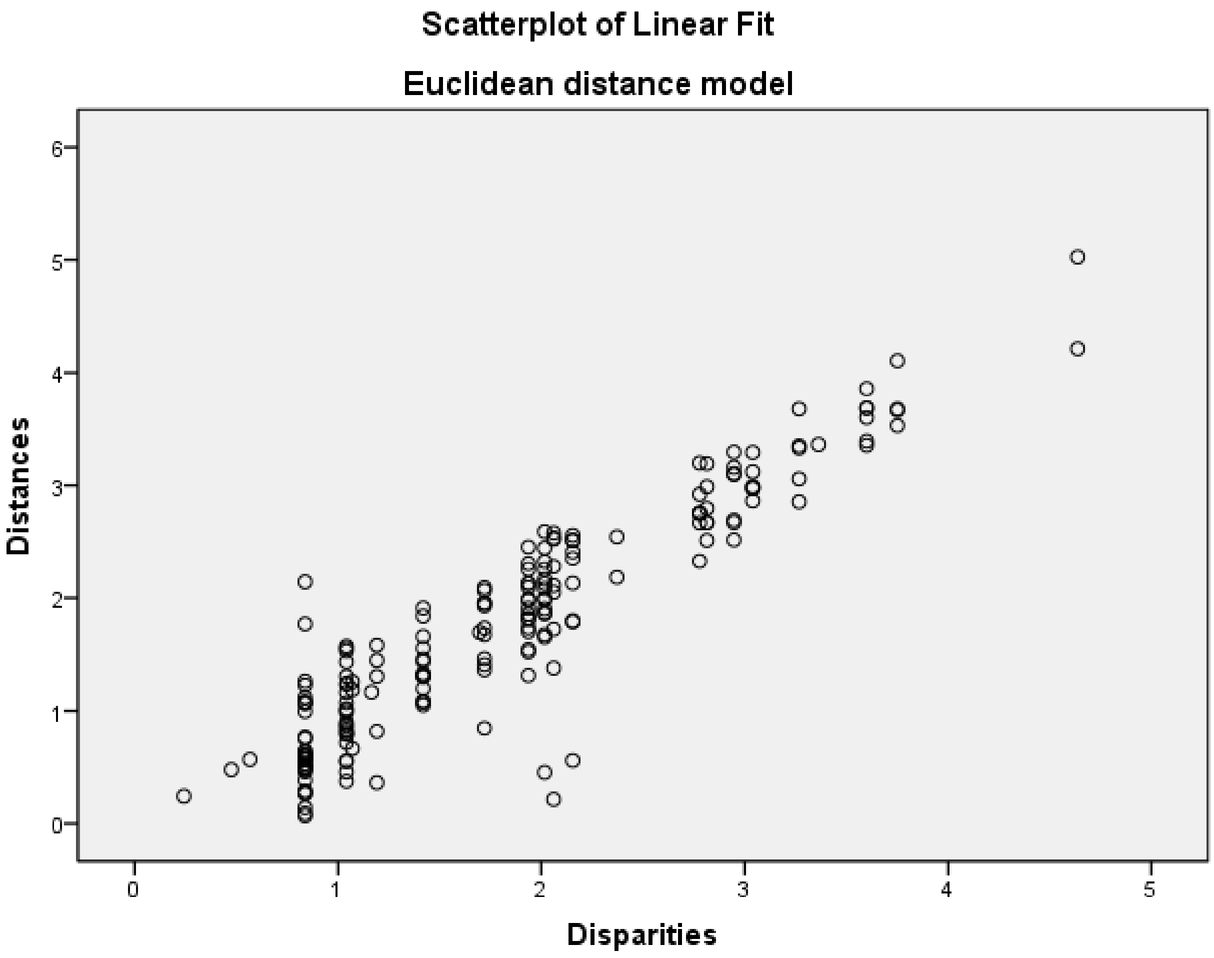
Scatterplot of Linear Fit
Euclidean distance model
Distances
Disparities
0
1
2
3
4
5
6

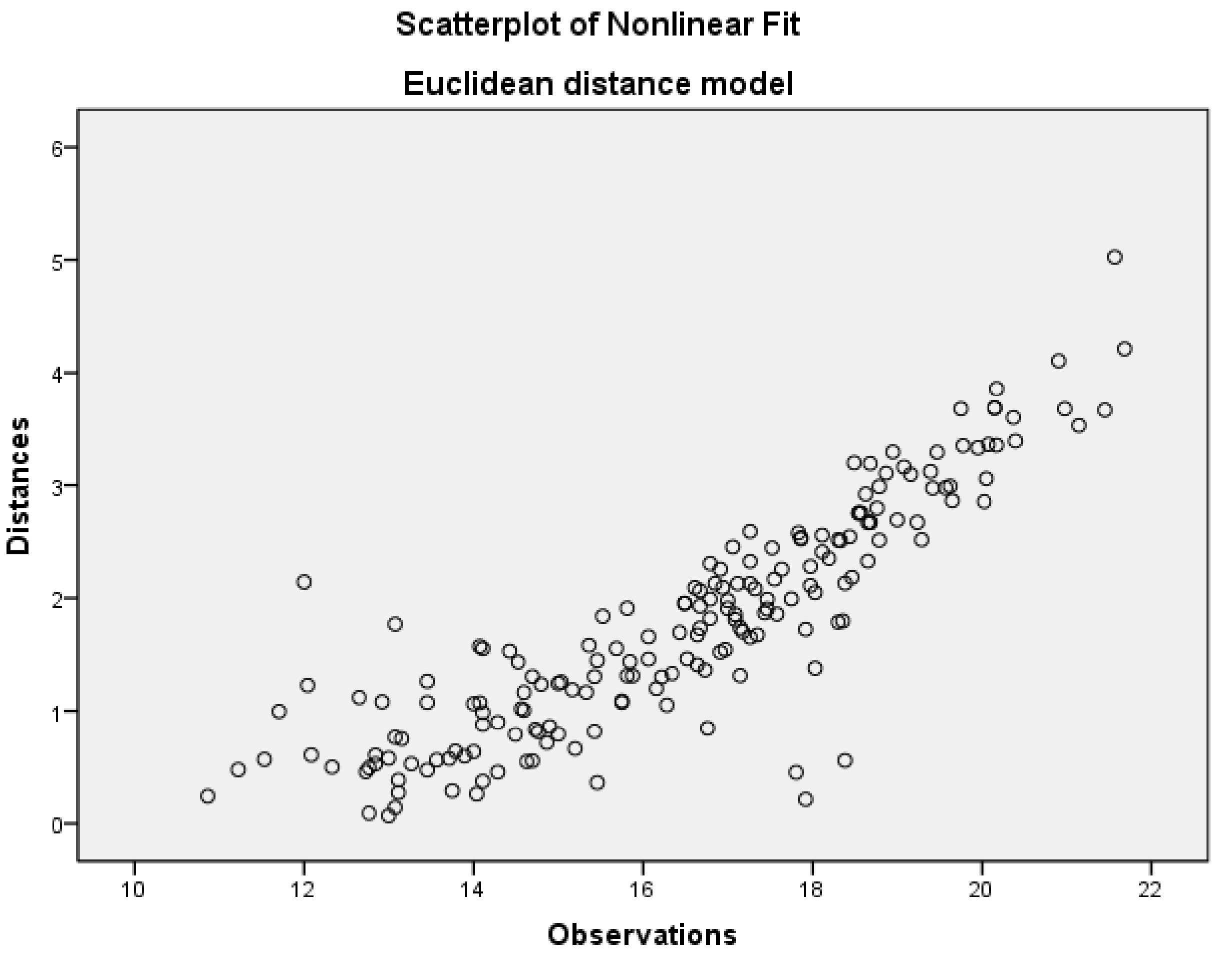
Scatterplot of Nonlinear Fit
Euclidean distance model
Distances
0
1
2
3
4
5
6
10
12
14
16
18
20
22
Observations

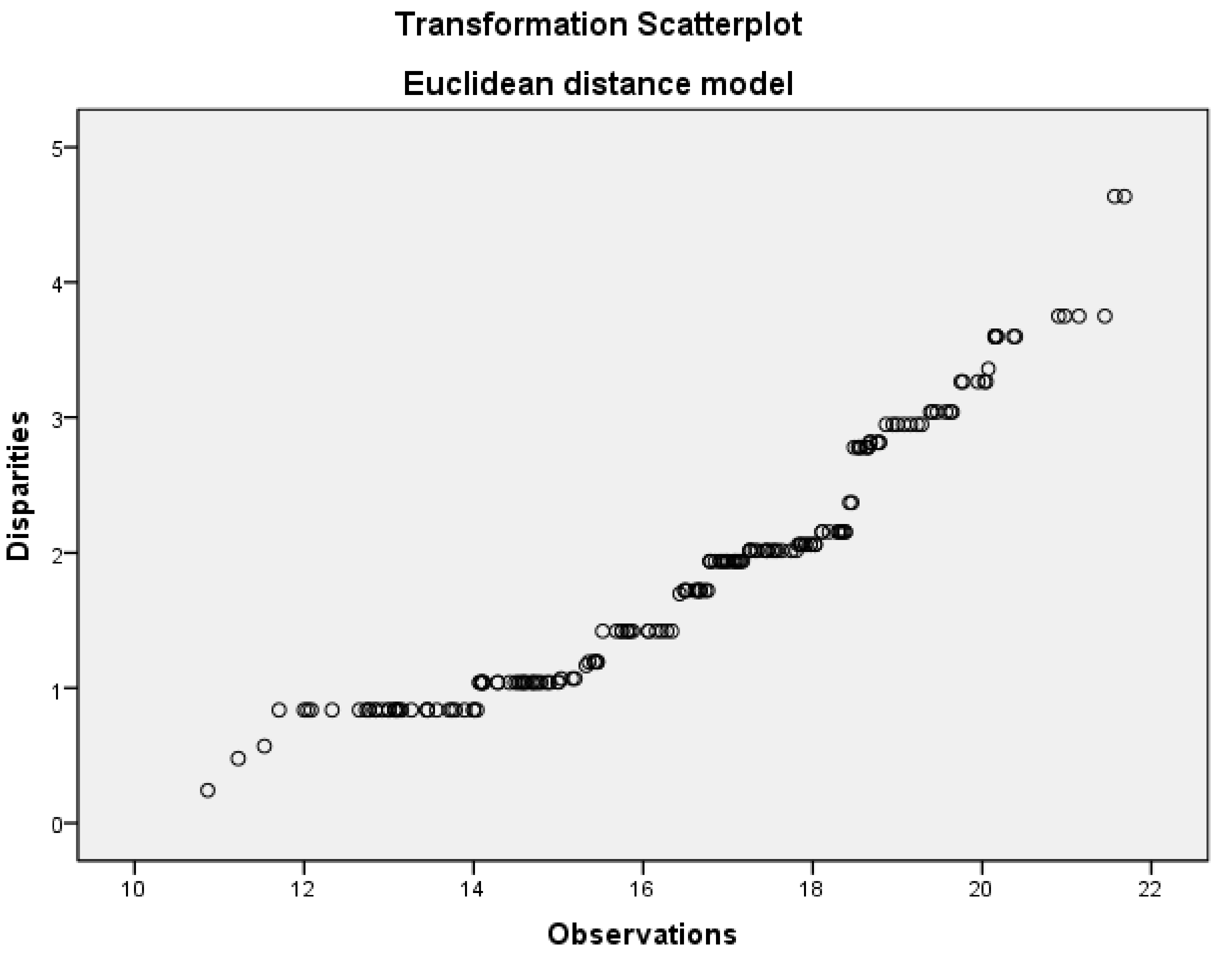

Transformation Scatterplot
Euclidean distance model
Disparities
Observations
0
1
2
3
4
5
10
12
14
16
18
20
22

```
Alscal Procedure Options



Data Options-

Number of Rows (Observations/Matrix).   20
Number of Columns (Variables) .  .  .   20
Number of Matrices   .  .  .  .  .  .    1
Measurement Level .  .  .  .  .  .  .    Ordinal
Data Matrix Shape .  .  .  .  .  .  .    Symmetric
Type  .  .  .  .  .  .  .  .  .  .  .    Dissimilarity
Approach to Ties  .  .  .  .  .  .  .    Leave Tied
Conditionality .  .  .  .  .  .  .  .    Matrix
Data Cutoff at .  .  .  .  .  .  .  .     .000000


Model Options-

Model .  .  .  .  .  .  .  .  .  .  .    Euclid
Maximum Dimensionality  .  .  .  .  .    3
Minimum Dimensionality  .  .  .  .  .    2
Negative Weights  .  .  .  .  .  .  .    Not Permitted


Output Options-

Job Option Header .  .  .  .  .  .  .    Printed
Data Matrices  .  .  .  .  .  .  .  .    Not Printed
Configurations and Transformations  .    Plotted
Output Dataset .  .  .  .  .  .  .  .    Not Created
Initial Stimulus Coordinates  .  .  .    Computed


Algorithmic Options-

Maximum Iterations   .  .  .  .  .  .       30
Convergence Criterion   .  .  .  .  .     .00100
Minimum S-stress  .  .  .  .  .  .  .     .00500
Missing Data Estimated by  .  .  .  .    Ulbounds
Tiestore .  .  .  .  .  .  .  .  .  .  190

Iteration history for the 3 dimensional solution (in squared distances)

                  Young's S-stress formula 1 is used.

                Iteration     S-stress      Improvement

                    1           .30199
                    2           .18531         .11668
```

3	.16856	.01675
4	.16374	.00482
5	.16254	.00120
6	.16211	.00042

Iterations stopped because
S-stress improvement is less than .001000

Stress and squared correlation (RSQ) in distances

RSQ values are the proportion of variance of the scaled data (disparities)
in the partition (row, matrix, or entire data) which
is accounted for by their corresponding distances.
Stress values are Kruskal's stress formula 1.

For matrix
Stress = .13276 RSQ = .90630

Configuration derived in 3 dimensions

Stimulus Coordinates

Stimulus Number	Stimulus Name	Dimension 1	2	3
1	notintun	1.4597	.6496	.0316
2	lackcomp	-1.7896	1.2373	-1.6409
3	noturnto	-1.7592	1.0560	-.0617
4	notalone	-.3392	1.0169	-.4290
5	notpartg	2.0649	.7473	.4787
6	notincom	.2497	-.2338	.2033
7	notclose	.1654	-.0164	-.3921
8	notshare	.3409	-.7820	-.4891
9	notoutgo	1.4152	-1.7058	2.2209
10	noclosep	.3718	.1621	.2617
11	leftout	-.7109	.3829	-.2130
12	meaningl	-1.8930	-.4812	1.2259
13	noonekno	-1.9991	-2.0154	-.8205
14	isolated	.1083	.5165	-.0794
15	notfindc	1.0913	-.3245	-.3948
16	ppledont	.0607	-.9478	-.4528
17	shy	-1.3939	.4602	1.6335
18	feelalon	-.1208	.9014	.1129
19	nopeople	1.5913	-.1770	-.3469
20	nopeop_1	1.0866	-.4463	-.8485

Iteration history for the 2 dimensional solution (in squared distances)

Young's S-stress formula 1 is used.

Iteration	S-stress	Improvement
1	.35777	
2	.24104	.11673
3	.21938	.02166
4	.21530	.00408
5	.21448	.00082

Iterations stopped because
S-stress improvement is less than .001000

Stress and squared correlation (RSQ) in distances

RSQ values are the proportion of variance of the scaled data (disparities)
in the partition (row, matrix, or entire data) which
is accounted for by their corresponding distances.
Stress values are Kruskal's stress formula 1.

For matrix
Stress = .19005 RSQ = .85156

Configuration derived in 2 dimensions

Stimulus Coordinates

Stimulus Number	Stimulus Name	Dimension 1	Dimension 2
1	notintun	1.2301	.5752
2	lackcomp	-1.5062	1.7478
3	noturnto	-1.5212	.4340
4	notalone	-.3024	.9689
5	notpartg	1.8357	.6131
6	notincom	.2395	-.1507
7	notclose	.0186	.3290
8	notshare	.1798	-.1159
9	notoutgo	1.6788	-2.1385
10	noclosep	.3935	.0353
11	leftout	-.4338	.3860
12	meaningl	-1.7074	-.9160
13	noonekno	-1.9064	-1.3220

14	isolated	-.0599	.3766
15	notfindc	.9700	.0764
16	ppledont	.1345	-.6692
17	shy	-1.4956	-.9449
18	feelalon	-.2294	.5915
19	nopeople	1.4585	.1785
20	nopeop_1	1.0232	-.0551

Abbreviated Name	Extended Name
feelalon	feelalonearoundothers
lackcomp	lackcompanionship
meaningl	meaningless
noclosep	noclosepeople
nooneknо	nooneknowsme
nopeop_1	nopeopletoturnto
nopeople	nopeopletotalkto
notclose	notcloseany
notfindc	notfindcomp
notincom	notincommon
notintun	notintune
notoutgo	notoutgoing
notpartg	notpartgroup
notshare	notshared
ppledont	ppledontunderstandme
