# Supplementary material for: The Mental Representation of Social Connections: Generalizability Extended to Beijing Adults
Source: PLoS One. 2012 Sep 11;7(9):e44065. doi: 10.1371/journal.pone.0044065 (PMC3442957; doi:10.1371/journal.pone.0044065)
Supplement: Table S1 — Correlation matrix of Revised UCLA Loneliness Scale items in Beijing young adults (N = 266). (DOCX) [file pone.0044065.s005.docx]

Table S1. Correlation matrix of Revised UCLA Loneliness Scale items in Beijing young adults in Study 1 (N=266).

Correlations

|  |  | ucla1 | ucla2 | ucla3 | ucla4 | ucla5 | ucla6 | ucla7 | ucla8 | ucla9 | ucla10 | ucla11 | ucla12 | ucla13 | ucla14 | ucla15 | ucla16 | ucla17 | ucla18 | ucla19 | ucla20 |
| --- | --- | --- | --- | --- | --- | --- | --- | --- | --- | --- | --- | --- | --- | --- | --- | --- | --- | --- | --- | --- | --- |
| ucla1 | Pearson Correlation | 1 |  |  |  |  |  |  |  |  |  |  |  |  |  |  |  |  |  |  |  |
|  | Sig. (2-tailed) |  |  |  |  |  |  |  |  |  |  |  |  |  |  |  |  |  |  |  |  |
| ucla2 | Pearson Correlatio­­n | -.093 | 1 |  |  |  |  |  |  |  |  |  |  |  |  |  |  |  |  |  |  |
|  | Sig. (2-tailed) | .128 |  |  |  |  |  |  |  |  |  |  |  |  |  |  |  |  |  |  |  |
| ucla3 | Pearson Correlation | -.240** | .420** | 1 |  |  |  |  |  |  |  |  |  |  |  |  |  |  |  |  |  |
|  | Sig. (2-tailed) | .000 | .000 |  |  |  |  |  |  |  |  |  |  |  |  |  |  |  |  |  |  |
| ucla4 | Pearson Correlation | -.188** | .371** | .331** | 1 |  |  |  |  |  |  |  |  |  |  |  |  |  |  |  |  |
|  | Sig. (2-tailed) | .002 | .000 | .000 |  |  |  |  |  |  |  |  |  |  |  |  |  |  |  |  |  |
| ucla5 | Pearson Correlation | .258** | -.212** | -.359** | -.292** | 1 |  |  |  |  |  |  |  |  |  |  |  |  |  |  |  |
|  | Sig. (2-tailed) | .000 | .001 | .000 | .000 |  |  |  |  |  |  |  |  |  |  |  |  |  |  |  |  |
| ucla6 | Pearson Correlation | .232** | -.124* | -.276** | -.200** | .305** | 1 |  |  |  |  |  |  |  |  |  |  |  |  |  |  |
|  | Sig. (2-tailed) | .000 | .043 | .000 | .001 | .000 |  |  |  |  |  |  |  |  |  |  |  |  |  |  |  |
| ucla7 | Pearson Correlation | -.227** | .278** | .366** | .250** | -.236** | -.224** | 1 |  |  |  |  |  |  |  |  |  |  |  |  |  |
|  | Sig. (2-tailed) | .000 | .000 | .000 | .000 | .000 | .000 |  |  |  |  |  |  |  |  |  |  |  |  |  |  |
| ucla8 | Pearson Correlation | -.191** | .248** | .204** | .292** | -.157* | -.343** | .292** | 1 |  |  |  |  |  |  |  |  |  |  |  |  |
|  | Sig. (2-tailed) | .002 | .000 | .001 | .000 | .011 | .000 | .000 |  |  |  |  |  |  |  |  |  |  |  |  |  |
| ucla9 | Pearson Correlation | .281** | -.239** | -.296** | -.193** | .280** | .347** | -.157* | -.171** | 1 |  |  |  |  |  |  |  |  |  |  |  |
|  | Sig. (2-tailed) | .000 | .000 | .000 | .002 | .000 | .000 | .010 | .005 |  |  |  |  |  |  |  |  |  |  |  |  |
| ucla10 | Pearson Correlation | .169** | -.207** | -.277** | -.266** | .338** | .190** | -.496** | -.140* | .168** | 1 |  |  |  |  |  |  |  |  |  |  |
|  | Sig. (2-tailed) | .006 | .001 | .000 | .000 | .000 | .002 | .000 | .023 | .006 |  |  |  |  |  |  |  |  |  |  |  |
| ucla11 | Pearson Correlation | -.192** | .316** | .350** | .345** | -.180** | -.222** | .170** | .149* | -.207** | -.243** | 1 |  |  |  |  |  |  |  |  |  |
|  | Sig. (2-tailed) | .002 | .000 | .000 | .000 | .003 | .000 | .005 | .015 | .001 | .000 |  |  |  |  |  |  |  |  |  |  |
| ucla12 | Pearson Correlation | -.086 | .170** | .333** | .189** | -.230** | -.087 | .248** | .150* | -.025 | -.203** | .290** | 1 |  |  |  |  |  |  |  |  |
|  | Sig. (2-tailed) | .163 | .005 | .000 | .002 | .000 | .156 | .000 | .015 | .689 | .001 | .000 |  |  |  |  |  |  |  |  |  |
| ucla13 | Pearson Correlation | -.173** | .303** | .432** | .367** | -.289** | -.257** | .315** | .333** | -.159** | -.377** | .312** | .295** | 1 |  |  |  |  |  |  |  |
|  | Sig. (2-tailed) | .005 | .000 | .000 | .000 | .000 | .000 | .000 | .000 | .009 | .000 | .000 | .000 |  |  |  |  |  |  |  |  |
| ucla14 | Pearson Correlation | -.249** | .319** | .375** | .429** | -.308** | -.360** | .359** | .369** | -.224** | -.227** | .410** | .324** | .394** | 1 |  |  |  |  |  |  |
|  | Sig. (2-tailed) | .000 | .000 | .000 | .000 | .000 | .000 | .000 | .000 | .000 | .000 | .000 | .000 | .000 |  |  |  |  |  |  |  |
| ucla15 | Pearson Correlation | .309** | -.324** | -.505** | -.325** | .384** | .189** | -.283** | -.196** | .357** | .330** | -.266** | -.161** | -.377** | -.321** | 1 |  |  |  |  |  |
|  | Sig. (2-tailed) | .000 | .000 | .000 | .000 | .000 | .002 | .000 | .001 | .000 | .000 | .000 | .009 | .000 | .000 |  |  |  |  |  |  |
| ucla16 | Pearson Correlation | .182** | -.253** | -.360** | -.254** | .343** | .312** | -.291** | -.289** | .235** | .446** | -.201** | -.185** | -.701** | -.327** | .418** | 1 |  |  |  |  |
|  | Sig. (2-tailed) | .003 | .000 | .000 | .000 | .000 | .000 | .000 | .000 | .000 | .000 | .001 | .002 | .000 | .000 | .000 |  |  |  |  |  |
| ucla17 | Pearson Correlation | -.116 | .159** | .211** | .313** | -.065 | -.075 | .119 | .133* | -.113 | -.009 | .255** | .197** | .162** | .276** | -.117 | -.040 | 1 |  |  |  |
|  | Sig. (2-tailed) | .059 | .009 | .001 | .000 | .290 | .222 | .053 | .030 | .066 | .880 | .000 | .001 | .008 | .000 | .057 | .514 |  |  |  |  |
| ucla18 | Pearson Correlation | -.177** | .343** | .329** | .329** | -.292** | -.290** | .244** | .272** | -.328** | -.197** | .361** | .311** | .330** | .463** | -.275** | -.278** | .205** | 1 |  |  |
|  | Sig. (2-tailed) | .004 | .000 | .000 | .000 | .000 | .000 | .000 | .000 | .000 | .001 | .000 | .000 | .000 | .000 | .000 | .000 | .001 |  |  |  |
| ucla19 | Pearson Correlation | .299** | -.270** | -.417** | -.158** | .282** | .219** | -.322** | -.199** | .243** | .327** | -.338** | -.162** | -.360** | -.160** | .418** | .334** | -.044 | -.206** | 1 |  |
|  | Sig. (2-tailed) | .000 | .000 | .000 | .010 | .000 | .000 | .000 | .001 | .000 | .000 | .000 | .008 | .000 | .009 | .000 | .000 | .471 | .001 |  |  |
| ucla20 | Pearson Correlation | .283** | -.286** | -.652** | -.295** | .306** | .261** | -.316** | -.195** | .249** | .319** | -.309** | -.292** | -.396** | -.317** | .509** | .388** | -.154* | -.252** | .562** | 1 |
|  | Sig. (2-tailed) | .000 | .000 | .000 | .000 | .000 | .000 | .000 | .001 | .000 | .000 | .000 | .000 | .000 | .000 | .000 | .000 | .012 | .000 | .000 |  |

**. Correlation is significant at the 0.01 level (2-tailed).

*. Correlation is significant at the 0.05 level (2-tailed).
