# Supplementary material for: The Mental Representation of Social Connections: Generalizability Extended to Beijing Adults
Source: PLoS One. 2012 Sep 11;7(9):e44065. doi: 10.1371/journal.pone.0044065 (PMC3442957; doi:10.1371/journal.pone.0044065)
Supplement: Table S2 — Correlation matrix of Revised UCLA Loneliness Scale items in Chinese middle-aged adults (N = 246). (DOCX) [file pone.0044065.s006.docx]

Table S2. Correlation matrix of Revised UCLA Loneliness Scale items in Chinese middle-aged adults in Study 2 (N=246).

Correlations

|  |  | Item 1 | Item 2 | Item 3 | Item 4 | Item 5 | Item 6 | Item 7 | Item 8 | Item 9 | Item 10 | Item 11 | Item 12 | Item 13 | Item 14 | Item 15 | Item 16 | Item 17 | Item 18 | Item 19 | Item 20 |
| --- | --- | --- | --- | --- | --- | --- | --- | --- | --- | --- | --- | --- | --- | --- | --- | --- | --- | --- | --- | --- | --- |
| Item 1 | Pearson Correlation | 1 |  |  |  |  |  |  |  |  |  |  |  |  |  |  |  |  |  |  |  |
|  | Sig. (2-tailed) |  |  |  |  |  |  |  |  |  |  |  |  |  |  |  |  |  |  |  |  |
| Item 2 | Pearson Correlatio­­n | -.212** | 1 |  |  |  |  |  |  |  |  |  |  |  |  |  |  |  |  |  |  |
|  | Sig. (2-tailed) | .001 |  |  |  |  |  |  |  |  |  |  |  |  |  |  |  |  |  |  |  |
| Item 3 | Pearson Correlation | -.254** | .413** | 1 |  |  |  |  |  |  |  |  |  |  |  |  |  |  |  |  |  |
|  | Sig. (2-tailed) | .000 | .000 |  |  |  |  |  |  |  |  |  |  |  |  |  |  |  |  |  |  |
| Item 4 | Pearson Correlation | -.308** | .600** | .437** | 1 |  |  |  |  |  |  |  |  |  |  |  |  |  |  |  |  |
|  | Sig. (2-tailed) | .000 | .000 | .000 |  |  |  |  |  |  |  |  |  |  |  |  |  |  |  |  |  |
| Item 5 | Pearson Correlation | .441** | -.077 | -.110 | -.179** | 1 |  |  |  |  |  |  |  |  |  |  |  |  |  |  |  |
|  | Sig. (2-tailed) | .000 | .228 | .086 | .005 |  |  |  |  |  |  |  |  |  |  |  |  |  |  |  |  |
| Item 6 | Pearson Correlation | .333** | -.219** | -.205** | -.280** | .373** | 1 |  |  |  |  |  |  |  |  |  |  |  |  |  |  |
|  | Sig. (2-tailed) | .000 | .001 | .001 | .000 | .000 |  |  |  |  |  |  |  |  |  |  |  |  |  |  |  |
| Item 7 | Pearson Correlation | -.302** | .354** | .320** | .416** | -.309** | -.365** | 1 |  |  |  |  |  |  |  |  |  |  |  |  |  |
|  | Sig. (2-tailed) | .000 | .000 | .000 | .000 | .000 | .000 |  |  |  |  |  |  |  |  |  |  |  |  |  |  |
| Item 8 | Pearson Correlation | -.249** | .239** | .213** | .392** | -.148* | -.392** | .471** | 1 |  |  |  |  |  |  |  |  |  |  |  |  |
|  | Sig. (2-tailed) | .000 | .000 | .001 | .000 | .020 | .000 | .000 |  |  |  |  |  |  |  |  |  |  |  |  |  |
| Item 9 | Pearson Correlation | .176** | -.054 | -.034 | -.087 | .221** | .163* | -.064 | -.087 | 1 |  |  |  |  |  |  |  |  |  |  |  |
|  | Sig. (2-tailed) | .006 | .400 | .600 | .175 | .000 | .010 | .317 | .174 |  |  |  |  |  |  |  |  |  |  |  |  |
| Item 10 | Pearson Correlation | .354** | -.271** | -.359** | -.362** | .281** | .463** | -.597** | -.338** | .165** | 1 |  |  |  |  |  |  |  |  |  |  |
|  | Sig. (2-tailed) | .000 | .000 | .000 | .000 | .000 | .000 | .000 | .000 | .009 |  |  |  |  |  |  |  |  |  |  |  |
| Item 11 | Pearson Correlation | -.296** | .430** | .447** | .550** | -.202** | -.328** | .473** | .423** | -.126* | -.452** | 1 |  |  |  |  |  |  |  |  |  |
|  | Sig. (2-tailed) | .000 | .000 | .000 | .000 | .001 | .000 | .000 | .000 | .048 | .000 |  |  |  |  |  |  |  |  |  |  |
| Item 12 | Pearson Correlation | -.335** | .286** | .413** | .339** | -.177** | -.336** | .346** | .292** | -.119 | -.408** | .404** | 1 |  |  |  |  |  |  |  |  |
|  | Sig. (2-tailed) | .000 | .000 | .000 | .000 | .005 | .000 | .000 | .000 | .063 | .000 | .000 |  |  |  |  |  |  |  |  |  |
| Item 13 | Pearson Correlation | -.328** | .328** | .325** | .414** | -.206** | -.414** | .410** | .409** | -.103 | -.415** | .415** | .370** | 1 |  |  |  |  |  |  |  |
|  | Sig. (2-tailed) | .000 | .000 | .000 | .000 | .001 | .000 | .000 | .000 | .108 | .000 | .000 | .000 |  |  |  |  |  |  |  |  |
| Item 14 | Pearson Correlation | -.241** | .348** | .393** | .498** | -.257** | -.404** | .528** | .378** | -.030 | -.551** | .505** | .413** | .439**. | 1 |  |  |  |  |  |  |
|  | Sig. (2-tailed) | .000 | .000 | .000 | .000 | .000 | .000 | .000 | .000 | .639 | .000 | .000 | .000 | .000 |  |  |  |  |  |  |  |
| Item 15 | Pearson Correlation | .258** | -.226** | -.197** | -.303** | .308** | .301** | -.378** | -.315** | .260** | .349** | -.331** | -.090 | -.417** | .371** | 1 |  |  |  |  |  |
|  | Sig. (2-tailed) | .000 | .000 | .002 | .000 | .000 | .000 | .000 | .000 | .000 | .000 | .000 | .157 | .000 | .000 |  |  |  |  |  |  |
| Item 16 | Pearson Correlation | .305** | -.215** | -.241** | -.311** | .253** | .452** | -.391** | -.392** | .136* | .502** | -.314** | -.270** | -.750** | -.471** | .555** | 1 |  |  |  |  |
|  | Sig. (2-tailed) | .000 | .001 | .000 | .000 | .000 | .000 | .000 | .000 | .033 | .000 | .000 | .000 | .000 | .000 | .000 |  |  |  |  |  |
| Item 17 | Pearson Correlation | -.225** | .305** | .322** | .427** | -.198** | -.278** | .358** | .333** | -.119 | -.352** | .371** | .352** | .344** | .435** | -.215** | -.297** | 1 |  |  |  |
|  | Sig. (2-tailed) | .000 | .000 | .000 | .000 | .002 | .000 | .000 | .000 | .063 | .000 | .000 | .000 | .000 | .000 | .001 | .000 |  |  |  |  |
| Item 18 | Pearson Correlation | -.281** | .362** | .440** | .590** | -.290** | -.393** | .378** | .383** | -.108 | -.378** | .559** | .349** | .418** | .560** | -.348** | -.365** | .429** | 1 |  |  |
|  | Sig. (2-tailed) | .000 | .000 | .000 | .000 | .000 | .000 | .000 | .000 | .092 | .000 | .000 | .000 | .000 | .000 | .000 | .000 | .000 |  |  |  |
| Item 19 | Pearson Correlation | .281** | -.135* | -.112 | -.219** | .368** | .268** | -.271** | -.221** | .168** | .287** | -.210** | -.180** | -.349** | -.344** | .469** | .488** | -.230** | -.204** | 1 |  |
|  | Sig. (2-tailed) | .000 | .035 | .079 | .001 | .000 | .000 | .000 | .000 | .008 | .000 | .001 | .005 | .000 | .000 | .000 | .000 | .000 | .001 |  |  |
| Item 20 | Pearson Correlation | .244** | -.248** | -.322** | -.217** | .243** | .256** | -.290** | -.197** | .185** | .356** | -.277** | -.163* | -.376** | -.329** | .476** | .549** | -.240** | -.294** | .519** | 1 |
|  | Sig. (2-tailed) | .000 | .000 | .000 | .001 | .000 | .000 | .000 | .002 | .004 | .000 | .000 | .011 | .000 | .000 | .000 | .000 | .000 | .000 | .000 |  |

**. Correlation is significant at the 0.01 level (2-tailed).

*. Correlation is significant at the 0.05 level (2-tailed).
